# Peripheral Nerve–Cancer Interactions in the Tumor Microenvironment: A Three-Dimensional Framework Integrating Mechanisms, Modulators, and Therapeutic Strategies

**DOI:** 10.34133/research.1221

**Published:** 2026-04-01

**Authors:** Fanglin Shao, Jie Wang, Aiqing Li, Ruicheng Wu, Jiamin Chen, Zhouting Tuo, Dilinaer Wusiman, Koo Han Yoo, Wuran Wei, Zhipeng Wang, Dengxiong Li, Qi Zhang, Yuanning Guo, Dechao Feng

**Affiliations:** ^1^ Urology & Nephrology Center, Department of Urology, Zhejiang Provincial People’s Hospital (Affiliated People’s Hospital), Hangzhou Medical College, Hangzhou 310014, Zhejiang, China.; ^2^ Department of Urology, Institute of Urology, West China Hospital, Sichuan University, Chengdu 610041, China.; ^3^ The Center of Psychosomatic Medicine, Sichuan Provincial People’s Hospital, University of Electronic Science and Technology of China, Chengdu 610072, China.; ^4^ Division of Surgery & Interventional Science, University College London, London W1W 7TS, UK.; ^5^ West China Biomedical Big Data Center, West China Hospital, Sichuan University, Chengdu 610041, China.; ^6^ Department of Urology, Tianjin Institute of Urology, The Second Hospital of Tianjin Medical University, Tianjin, China.; ^7^ Department of Comparative Pathobiology, College of Veterinary Medicine, Purdue University, West Lafayette, IN 47907, USA.; ^8^ Purdue Institute for Cancer Research, Purdue University, West Lafayette, IN 47907, USA.; ^9^ Department of Urology, Kyung Hee University, Seoul, South Korea.; ^10^ Department of Urology, Sichuan Provincial People’s Hospital, University of Electronic Science and Technology of China, Chengdu 610072, China.; ^11^ Department of Urology, The First Affiliated Hospital of Zhejiang Chinese Medical University (Zhejiang Provincial Hospital of Chinese Medicine), Hangzhou, Zhejiang Province, China.; ^12^ Rappaport Family Institute for Research in the Medical Sciences, Technion–Israel Institute of Technology, Haifa 31096, Israel.; ^13^ Department of Genetics and Developmental Biology, The Ruth and Bruce Rappaport Faculty of Medicine, Technion–Israel Institute of Technology, Haifa 31096, Israel.

## Abstract

The nervous system has emerged as a critical regulator of tumor biology. Over the past 2 decades, accumulating evidence has given rise to the rapidly expanding field of cancer neuroscience, revealing that neural circuits actively shape tumor initiation, progression, metastasis, prognosis, and therapeutic response. While the nervous system comprises both central and peripheral components, increasing attention has focused on the peripheral nervous system (PNS) as a key mediator of tumor–host interactions within the tumor microenvironment (TME). Autonomic (sympathetic, parasympathetic, and enteric) and sensory neural pathways interact dynamically with tumor cells, immune cells, glial cells, and other stromal components, influencing tumor growth, invasion, metastasis, and immune regulation through diverse cellular and molecular mechanisms. In addition, environmental and patient pathophysiological factors—including environmental stress, psychological states, pain, microbiota, aging, metabolic status, and lifestyle behaviors—can further modulate nerve–cancer interactions and reshape the tumor ecosystem. In this review, we systematically organize PNS–cancer interactions within the TME across 3 integrative dimensions: pathological phenotypes, cellular components, and modes of communication, thereby providing a conceptual framework for understanding the mechanistic landscape of cancer neuroscience. This structured perspective highlights the diverse modalities of nerve–tumor communication, identifies key modulatory factors influencing these interactions, and discusses emerging therapeutic strategies targeting nerve–cancer signaling.

## Introduction

Cancer profoundly impacts patients’ quality of life and remains the leading cause of mortality worldwide. In 2020, it was estimated that there would be approximately 10.3 million deaths related to cancer and 19.3 million new cases diagnosed. By 2040, this number is expected to rise to about 28.4 million new cases globally [1]. Current treatment methods are inadequate to address the needs of cancer patients due to their associated toxicity, adverse reactions, high costs, drug resistance, and various other limitations [2]. Despite advances in targeted therapy and immunotherapy, many patients still experience poor outcomes, highlighting the urgent need to identify novel mechanisms and therapeutic targets for cancer detection and treatment.

In recent years, the critical role of the nervous system in cancer initiation, progression, and metastasis has garnered substantial attention, offering a promising new perspective for understanding and treating malignancies [3–5]. Emerging evidence demonstrates that neural regulation within the tumor microenvironment (TME) profoundly influences tumor behavior, presenting potential opportunities for developing innovative therapeutic strategies [2].

The nervous system is anatomically divided into the central nervous system (CNS) and the peripheral nervous system (PNS), with the latter comprising the somatic, autonomic, and sensory subdivisions [6], whose coordinated actions regulate both physiological and pathological processes across organ systems. They are responsible for regulating organ growth, maintaining tissue homeostasis, sustaining overall physiological functions, as well as facilitating wound healing, defending against pathogens, and contributing to immunoregulation and regeneration throughout the life cycle [7]. Over the past 2 decades, numerous studies from multiple dimensions have revealed that the PNS plays extensive and pivotal roles across diverse tumors throughout the body, shaping every stage of tumor initiation and progression [8,9]. With the increasing depth of research, it has become evident that the mechanisms of nerve–cancer crosstalk often mirror fundamental principles of neurophysiology and neuropathology. Some interactions may be viewed as a hijacking of neural biology by cancer, whereas others represent reciprocal adaptations between cancer and the PNS within the TME or even at the organismal level [8,9]. These investigations have greatly broadened our understanding of neuroscience and opened new avenues for cancer management strategies.

Historically, perineural invasion (PNI)—defined as tumor spread and invasion along relatively mature peripheral nerves (at least those containing a perineurium) or within the nerve itself—was first reported by pathologists as early as the mid-1800s. PNI has since been recognized as a distinct form of metastasis, independent of lymphatic or vascular invasion, and has been documented across numerous solid tumors, where it correlates with aggressive phenotypes and poor patient survival [10]. With advances in biotechnology, recent years have seen substantial progress in elucidating the cellular and molecular mechanisms underlying PNI. Beyond PNI, which primarily involves larger and more mature nerves, small nerve fibers and nerve endings were also noted in several cancers since the early 1900s [11]. However, relatively few studies initially addressed their functional roles in carcinogenesis and regulation of the TME. Only in the past 2 decades has research increasingly focused on the capacity of nerves to be actively recruited into the TME via neurogenesis/axonogenesis and innervation. By releasing neurotrophic factors, neurotransmitters, neuropeptides, and other factors that directly modulate TME components, this shift has integrated functional neural studies into the cancer research paradigm, opening broader and more complex horizons and realm of nerve–cancer crosstalk [4].

It is worth emphasizing that the TME is highly heterogeneous and complex across the entire tumor, with distinct regions exhibiting markedly different phenotypic features due to variations in cellular composition and local molecular cues. Therefore, rather than examining the TME as a whole, focusing on specific microenvironmental niches has emerged as a new paradigm in cancer research, offering more precise therapeutic targets. Indeed, it has been proposed that the TME can be classified into several specialized microenvironments: the hypoxic niche, immune microenvironment, metabolic microenvironment, acidic niche, innervated niche, and mechanical microenvironment [12]. Among these, TME regions located within and adjacent to nerves comprise neurons, nerve fibers, glial cells, stromal cells, immune cells, and cancer cells, which collectively involve diverse modes of interaction mediated by molecules such as neurotrophic factors, neurotransmitters, neuropeptides, and neural adhesion molecules. Together, these components constitute a highly specialized innervated niche, encompassing both PNI niche and tumor neurogenesis/innervation niche.

The interactions, mediated by numerous cellular and signaling molecular components, influence tumor initiation, progression, prognosis, and therapeutic response across different stages of tumor development. Collectively, this body of work has established a new academic field: cancer neuroscience [4,8]. In the most recent update of the *Hallmarks of Cancer* framework, Douglas Hanahan moves beyond the earlier approach of enumerating discrete, cancer cell-intrinsic capabilities and instead reorganizes decades of accumulated knowledge into a systemic framework composed of 4 conceptually distinct yet interconnected parameter dimensions. Notably, nerve–cancer interactions are incorporated for the first time into this conceptual architecture, spanning 2 separate dimensions: “Innervation” under the category of Enabling Phenotypic Characteristics, and “Neurons and their axonal projections” within the dimension of Hallmark-Conveying Cells of the TME [13]. In the following sections, we systematically describe the diverse interactions in the innervated niche between the various components of the PNS and the cellular constituents of the TME, structured around 3 major dimensions: (a) pathological phenotypes, (b) cellular components, and (c) modes of communication, which together provide the organizational framework for the detailed review that follows.

Beyond the complex interactions between the nervous system and the TME, a variety of environmental and patient pathophysiological factors—including psychological states, environmental stressors, pain, microbiota, aging, and obesity, as well as lifestyle behaviors (such as smoking, diet, exercise, and circadian disruption)—substantially influence cancer development [14]. One plausible mechanism by which these factors affect cancer risk is through modulation of nerve–cancer interactions [5,15]. We next discuss how these extrinsic and intrinsic factors may shape nerve–cancer crosstalk. Notably, many of these influences remain to be directly demonstrated. By integrating these considerations, we aim to highlight key directions for future basic and clinical research.

In contrast to cancer neurobiology in the CNS, which is characterized by synaptic connections, neuronal electrical activity, plasticity of neural circuits, and direct electrochemical signaling between neurons and tumor cells or glial cells [16], interactions between peripheral nerves and tumors are more complex, primarily due to their emphasis on multiple forms of TME-mediated cellular crosstalk through biochemical signaling in peripheral solid tumors, such as head and neck, gastric, pancreatic, prostate, and breast cancers. These tumors account for over 95% of malignancies outside the CNS, highlighting their broader universality and clinical significance [2]. Therefore, this review focuses on the interplay among the PNS, tumor cells, and other cellular components of the TME, and how these interactions influence the development of cancer.

Finally, we outline the therapeutic implications of nerve–cancer interactions, including direct denervation, targeting nerve-derived paracrine signals such as neurotransmitters and neurotrophic factors, combining these approaches with tumor immunotherapy to modulate the tumor immune microenvironment, and addressing environmental/pathophysiological factors, for example, through psychosocial or behavioral interventions.

## Pathological Features of Peripheral Nerve–Cancer Interaction within the TME

### Dimension 1: Pathological phenotypes of nerve–cancer interactions within the TME

#### PNI—cancer mimicry of the nerve injury response within the TME

**Fig. 1.**
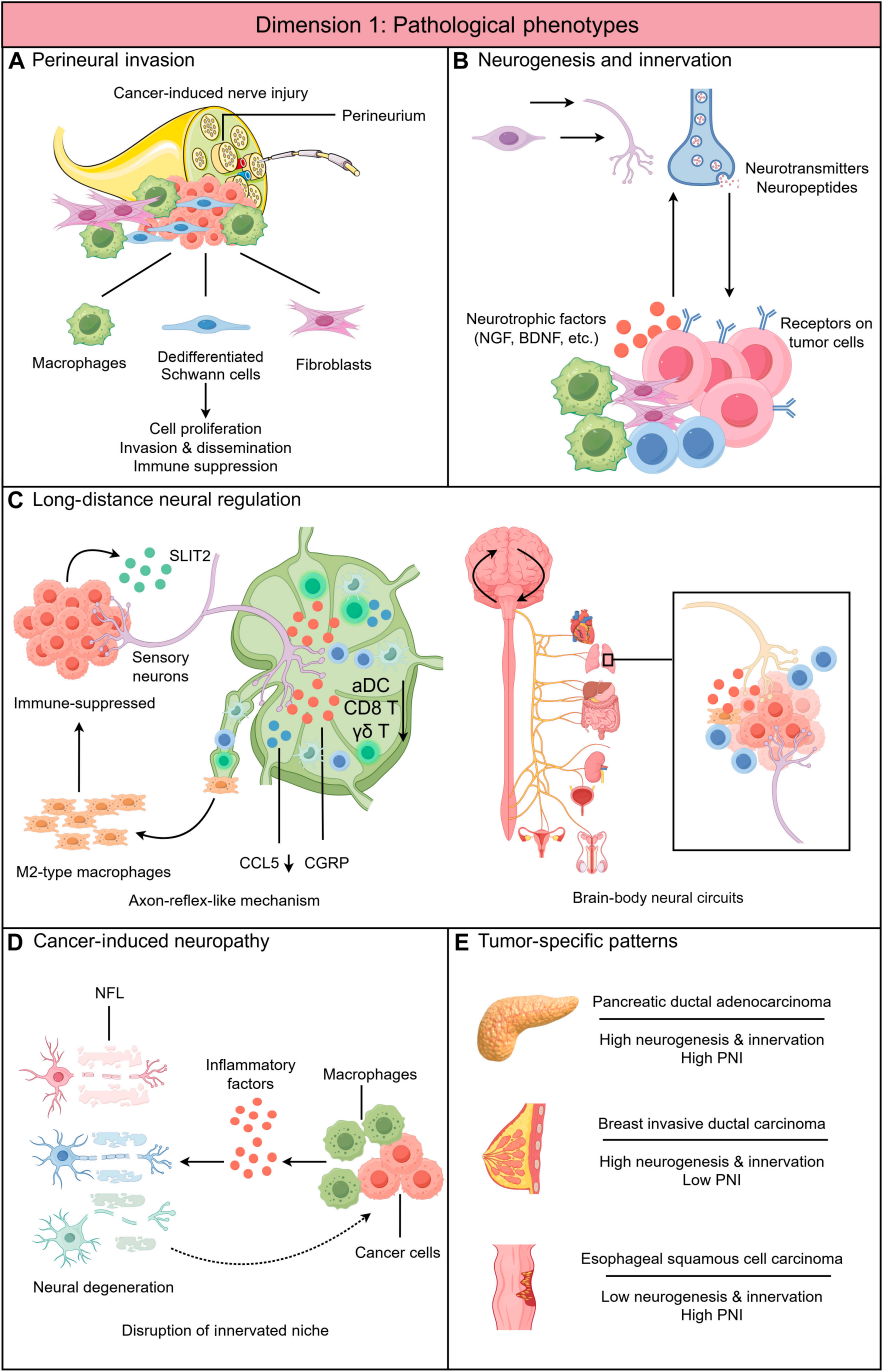
Pathological phenotypes: Dimension 1 of nerve–cancer interactions in the tumor microenvironment (TME). (A) Perineural invasion (PNI): A pathological process characterized by tumor cell migration toward and invasion along or within peripheral nerves, which are enclosed by the perineurium. With the PNI niche, Schwann cells, macrophages, and fibroblasts interact with tumor cells, which collectively facilitate tumor progression. (B) Neurogenesis and tumor innervation: In the context of malignancy, neurogenesis encompasses both neoneurogenesis—the mobilization of neural progenitor cells from the brain or the enteric nervous system (though not explicitly depicted in the figure)—and neuritogenesis/axonogenesis. This multi-stage process describes how newly formed or existing nerve fibers actively extend toward and infiltrate the tumor. This interaction, whether direct or mediated through various TME components (such as immune or stromal cells; omitted from the figure for simplicity), promotes tumor progression via the localized release of neurotransmitters and neuropeptides. (C) Long-distance neural regulation. Left panel: Tumors can exploit an axon-reflex-like mechanism, in which activation of nociceptive sensory fibers propagates to tumor-draining lymph nodes, increasing calcitonin gene-related peptide (CGRP) release and reprogramming the lymph node to orchestrate systemic and local immunosuppression TME. Right panel: Tumors can also hijack brain–body neural circuits, ultimately leading to activation of peripheral neural pathways and the release of immunosuppressive and tumor-promoting neurotransmitters within the TME. (D) Cancer-induced neuropathy: Tumor growth and associated inflammation can induce neural injury and dysfunction, leading to axonal damage, inflammatory remodeling, and imbalance of neural signaling. These alterations disrupt the physiological innervated niche and reshape the TME in ways that favor tumor progression and immune evasion. (E) Tumor-specific patterns of nerve–cancer interactions: The prevalence of different pathological phenotypes—such as PNI and tumor innervation/neurogenesis—varies substantially across cancer types, suggesting potential tumor- or organ-specific preferences in nerve–tumor interaction patterns. NGF, nerve growth factor; BDNF, brain-derived neurotrophic factor; SLIT2, slit guidance ligand 2; aDC, activated dendritic cell; CCL5, C–C motif chemokine ligand 5; NFL, neurofilament light chain.

PNI represents the earliest recognized pathological manifestation of nerve–cancer interactions. First documented by pathologists in the mid-19th century, PNI is characterized by tumor cell migration and invasion along mature peripheral nerves containing a perineurium, or infiltration into and within the nerve structure itself (Fig. 1A). This phenomenon is now recognized as a distinct metastatic route, separate from lymphatic or hematogenous dissemination, and can occur even in the absence of lymphatic or vascular invasion [10,17]. PNI can facilitate tumor spread to distant sites, often extending far beyond the margins of local invasion; in some cancers, PNI may even represent the sole route of metastatic dissemination [10]. Furthermore, PNI provides cancer cells with a protective neural niche, enabling them to survive neoadjuvant therapy across different chemotherapy regimens [18].

PNI has been observed across a broad spectrum of solid malignancies, including squamous cell carcinomas, adenocarcinomas, and basal cell carcinomas arising from multiple organs, as well as in certain leukemias, lymphomas, and sarcomas [17,19–25], where it is consistently associated with aggressive tumor behavior and diminished patient survival [9,10]. The prevalence of PNI varies substantially across tumor types, with pancreatic cancer (PC) exhibiting the highest incidence (80% to 100%), making it the most extensively studied malignancy in the context of PNI [9,26].

Advances in biotechnology over the past 3 decades have substantially enhanced our understanding of the cellular and molecular mechanisms driving PNI. Emerging evidence from cancer–nerve crosstalk studies, particularly those examining inflammatory responses and neurotrophic support mediated by monocytes/macrophages, Schwann cells (the principal glial cells of the PNS), and stromal cells (including fibroblasts and organ-specific stellate cells such as pancreatic stellate cells), suggests that cancer invasion along nerves recapitulates physiological nerve injury and regeneration processes in the innervated niche [26–30]—a coordinated response involving Schwann cell dedifferentiation and proliferation, macrophage recruitment and polarization, axonal regeneration, as well as extracellular matrix remodeling to restore nerve function following damage (Fig. 1A).

The bidirectional communication between cancer cells and neural elements—including neurons/axons, Schwann cells, endoneurial immune cells, and stromal components—occurs through multiple signaling pathways involving neurotrophins, glial cell-derived neurotrophic factor (GDNF), chemokines, cytokines, midkine family molecules, neural cell adhesion molecules, axon guidance cues, neurotransmitters, and neuropeptides [2,9,31].

Schwann cells are now recognized as the most important and active contributors to PNI rather than passive bystanders [32,33]. Multiple studies demonstrate that Schwann cells in the PNI niche acquire a dedifferentiated, nonmyelinating, or repair-like phenotype, likely triggered by nerve injury or tumor-induced neural remodeling [26,27,34]. Notably, Schwann cells can migrate toward cancer cells even before overt nerve invasion, a process mediated by tumor-derived nerve growth factor (NGF) acting on Schwann cell p75 neurotrophin receptor (p75NTR) [27].

Activated Schwann cells facilitate PNI through both paracrine and contact-dependent mechanisms. Schwann cell-derived L1 cell adhesion molecule (L1CAM) promotes cancer cell attraction and migration via mitogen-activated protein kinase (MAPK) activation and signal transducer and activator of transcription 3 (STAT3)-dependent up-regulation of metalloproteinases [35], while direct Schwann cell–cancer contact induces directional protrusions and intercalation through neural cell adhesion molecule 1 (NCAM1), driving cancer cell dispersion along nerves [26]. Schwann cells also secrete C–C motif chemokine ligand 2 (CCL2) to recruit CCR2^+^ monocytes/macrophages, which disrupt the perineurium through cathepsin B and further enhance nerve invasion [36,37].

Recent work has further revealed that nonmyelinating Schwann cells can be reprogrammed by cancer cells into tumor-activated Schwann cell tracks (TASTs), in which c-Jun-activated Schwann cells collectively organize into dynamic tracks that physically guide and mechanically promote cancer cell migration and invasion. These repair-like Schwann cell programs correlate with poor survival in pancreatic ductal adenocarcinoma (PDAC), highlighting a wound repair-mimicking mechanism hijacked by tumors [38].

Even in cancers where PNI is not a dominant determinant of outcome, such as cutaneous melanoma, Schwann cells also adopt a repair-like state resembling Wallerian degeneration, characterized by enhanced motility, extracellular matrix remodeling, neurotrophic factor secretion, and macrophage recruitment/polarization [34]. Moreover, activated Schwann cells can engage in neuroimmune crosstalk resembling reactive gliosis, contributing to cancer-associated analgesia and potentially masking disease progression [28].

Furthermore, Schwann cells exhibit marked metabolic plasticity that facilitates immune escape. In diabetes-associated PDAC, Schwann cells sense excess lactate in the diabetic TME and remodel into a METTL16^+^/CD276^+^/NECTIN2^+^ immunosuppressive phenotype, enabling them to suppress CD8^+^ T cell effector function and thereby accelerate tumor progression. Mechanistically, through METTL16 K269 lactylation-mediated stabilization of CCCTC-binding factor (CTCF), these Schwann cells up-regulate immune-evasion ligands such as CD276 and NECTIN2, creating a microenvironment that actively promotes tumor growth and confers resistance to PD-1 immunotherapy [39].

Monocytes and macrophages constitute another crucial component of the PNI niche and are actively recruited by tumor-derived chemokines, where they are subsequently polarized toward a tumor-associated (M2-like) phenotype [36,40,41]. Cancer cell-secreted colony stimulating factor 1 (CSF-1) drives macrophage chemotaxis via CSF-1 receptor signaling. In turn, macrophages promote PNI by releasing GDNF, which stimulates cancer cell migration toward nerves through Ret proto-oncogene (RET)/GDNF family receptor α1 (GFRα1) [40]. Additionally, soluble GFRα1 released from neural components (possibly by neurons, axons, Schwann cells, fibroblasts, or endoneurial macrophages) further cooperates with macrophage GDNF to enhance RET activation and facilitates PNI [42].

As a summary, these cellular and molecular interactions mirror those observed during physiological nerve injury responses in the innervated niche, involving dedifferentiated Schwann cells, macrophages, injured axons, stroma cells, and target tissues [9,43].

#### Neurogenesis and tumor innervation—Neural regulation of the TME

Beyond angiogenesis and lymphangiogenesis, accumulating evidence demonstrates that neurogenesis and innervation play pivotal roles in tumorigenesis and cancer progression [2,3]. In contrast to PNI, which primarily affects larger and more mature nerves containing a perineurium, neurogenesis and tumor innervation refer to the emergence, remodeling, and functional integration of newly formed small nerve fibers and nerve endings within tissues, thereby establishing an innervated niche in the TME (Fig. 1B). Although this phenomenon was already noted in several types of cancer in the early 1900s [11], it was not until the past 2 decades that their functional roles in carcinogenesis and the regulation of the TME began to be systematically investigated.

In the context of cancer–nerve interactions, the terminology requires clarification. Neuritogenesis, axonogenesis, and innervation collectively describe the process whereby newly formed axons or neurites actively extend into the TME and establish functional contacts with cancer, immune, and stromal cells, thereby modulating the TME and promoting tumor progression through the release of various neurotransmitters, neuropeptides, and other neuromodulatory factors [44,45] (Fig. 1B). The term neurogenesis can encompass broader meanings, referring not only to neuritogenesis and axonogenesis but also to de novo generation of neurons from neural progenitors (neoneurogenesis), with the precise definition being context-dependent [9,46,47]. In cancer neuroscience field, neoneurogenesis is more specifically used to denote the formation of new neurons within or adjacent to tumors from neural precursor populations [46]. In this review, we use the terms neurogenesis, neuritogenesis, and (tumor) innervation to collectively describe this spectrum of nerve–tumor remodeling processes.

Notably, unlike PNI, which primarily describes a pathological morphology and pattern of tumor invasion, this phenotype—neurogenesis/tumor innervation—highlights the functional regulatory role of certain specific types of nerves within the TME innervated niche (Fig. 1B). The field was pioneered in 2013 when Magnon et al. [48] provided rigorous experimental evidence demonstrating that intratumoral autonomic neurogenesis and neural signaling actively promote tumor progression. Since then, numerous laboratories have progressively identified various nerve types and subtypes that regulate tumor initiation and progression by modulating multiple cellular components within the TME.

Additionally, compared with PNI—which occurs during the invasive phase—neurogenesis and innervation participate throughout the entire course of tumor initiation and progression. Notably, they can emerge during early carcinogenesis, with evidence of their presence in precancerous lesions where they facilitate malignant transformation [9,46]. This temporal distinction underscores that neurogenesis and innervation involve mechanisms beyond cancer hijacking nerve injury responses, incorporating developmental processes such as neural-dependent embryogenesis and organogenesis.

During the stage of intraepithelial neoplasia, marked increases in innervation have been documented across multiple organs: adrenergic and nociceptive/sensory innervation in the pancreas, cholinergic innervation beneath the gastric epithelium, and adrenergic innervation in the prostate [49–52]. In certain cancer types, neurogenesis and innervation are partially driven by neurotrophic factors secreted by precancerous cells, which can directly activate oncogenic pathways or indirectly modulate TME activities through neurotransmitters and neuropeptides. These neuroactive molecules enhance angiogenesis and suppress antitumor immunity, thereby establishing positive feedback loops that promote tumorigenesis, invasion, and metastasis. Such pro-tumorigenic nerve–cancer interactions and signaling pathways are tumor type dependent and may be maintained, amplified, or modified during advanced cancer stages [44,50,52].

Remarkably, tumor recruitment of nerves extends beyond simple neuritogenesis or axonogenesis to encompass comprehensive neuronal reprogramming. A groundbreaking 2025 study employed a novel technology termed Trace-n-Seq—combining retrograde axonal tracing from tissues to their respective ganglia with single-cell isolation and transcriptomic analysis—revealing that PDAC actively remodels neurons rather than merely affecting axons. This neuronal reprogramming enhances interactions between neurons/nerves and multiple cellular components of the TME, including cancer cells, fibroblasts, and immune cells, thereby establishing and maintaining a niche conducive to cancer cell proliferation. At the neuronal subtype level, PDAC preferentially attracts neurofilament medium chain (NEFM)-positive neurons while reducing calcitonin gene-related peptide (CGRP)-positive nociceptive neurons. Transcriptomic analysis of over 5,000 neurons revealed that PC induces profound neuronal reprogramming characterized by substantial alterations in gene expression profiles related to neural development, axon guidance, and microenvironment processes, culminating in a distinctive pancreatic-cancer nerve (PCN) signature. Strikingly, even after tumor resection, neurons retain this PCN signature, potentially facilitating local recurrence [53].

Importantly, PNI and neurogenesis/innervation should not be viewed as entirely independent processes. Nerve injury and regeneration responses inherently incorporate aspects of neurogenesis and innervation; consequently, these phenomena share common mechanisms and frequently coexist in certain tumor types [10,54].

To highlight the most recent advances, it is worth noting that prior studies have mainly focused on the regulatory influence of the PNS on tumors—particularly the roles of the autonomic (sympathetic and parasympathetic) and nociceptive sensory nerves in shaping the TME. However, a critical missing link remained unresolved: Despite abundant evidence for neurotransmitter-mediated communication, it was unclear whether genuine synaptic structures exist between neurons and tumor cells, and whether such connections are functionally active.

In 2025, 3 landmark studies provided the first direct evidence of functional neuron–tumor synapses. The first, published in February, identified synapse-like functional contacts between peripheral neurons and gastric cancer cells—marking the first demonstration of synapse-like nerve–tumor connections in extracranial tumors [55]. Subsequently, 2 studies published in September extended this paradigm: one revealed, through electron microscopy, immunogold labeling, and coculture models, the presence of pseudo-synaptic junctions between sensory nerve terminals and PDAC cells capable of glutamate-mediated signaling [56]; the other, using electrophysiology, optogenetics, super-resolution microscopy, and viral tracing, identified both glutamatergic and γ-aminobutyric acid-ergic (GABAergic) synaptic structures in small cell lung cancer (SCLC), demonstrating functional activation of tumor *N*-methyl-d-aspartate (NMDA) and GABA_A receptors [57]. These discoveries not only bridge a long-standing gap in nerve–cancer crosstalk but also expand the concept of tumor innervation from mere nerve–tumor proximity to bona fide, functional nerve–tumor synaptic communication.

The mechanisms underlying neurogenesis and tumor innervation represent the most intensively investigated area in PNS cancer neuroscience research. A comprehensive discussion of these mechanisms, organized by neural subtype classification, will be presented in the section *PNS–tumor interactions within the TME*.

#### Cancer-induced neuropathy—Neural signaling disruption within the innervated niche

PNI primarily reflects the intrinsic invasive propensity of cancer cells. In this setting, neural injury is largely caused by mechanical compression or protease-mediated degradation of the myelin sheath (see the sections *PNI—Cancer mimicry of nerve injury response in the TME* and *Direct cell–cell contact*). However, direct physical invasion of nerves is not a prerequisite for tumor-associated neuropathy. Certain malignancies can induce noninvasive, degenerative neural alterations, in some respects resembling features of metabolic and inflammatory neuropathies such as diabetic neuropathy. Through the release of stress- and inflammation-associated mediators, metabolic by-products, or other tumor-derived microenvironmental signals, cancer cells can provoke axonal atrophy, reduced nerve fiber density, and impaired neural function. Such neuropathic changes disrupt the physiological balance of the innervated niche, reshaping the local tissue ecosystem in ways that favor tumor growth, immune evasion, and metastatic dissemination (Fig. 1D).

A paradigmatic example is acute myelogenous leukemia (AML) [58], in which leukemic cells do not display overt PNI but instead induce sympathetic neuropathy through alternative mechanisms, potentially involving inflammatory responses. AML infiltration leads to a marked reduction of TH^+^ sympathetic nerve fibers in the bone marrow (and spleen), accompanied by axonal fragmentation, decreased nerve density, and diminished sympathetic tone. Because sympathetic input is essential for maintaining the quiescence of the hematopoietic stem cell (HSC) niche—particularly the quiescence of Nestin^+^ mesenchymal stromal cells (MSCs)—its loss profoundly disrupts niche homeostasis. AML-induced sympathetic injury drives expansion of Nestin^+^ MSCs with bias toward osteoblastic differentiation, at the expense of NG2^+^ periarteriolar niche cells that normally support HSC maintenance. Consequently, the HSC niche collapses and becomes leukemia-biased, conferring a competitive advantage to leukemic stem cells (LSCs) and accelerating disease progression. Notably, pharmacological activation of β2-adrenergic receptors (β2-ARs) can partially restore niche homeostasis and counteract the ecological shift induced by sympathetic neuropathy.

Cancer-induced neuropathy is also common in solid tumors, although the resulting neural damage often needs to be carefully distinguished from treatment-related neurotoxicity, PNI, or metabolic and nutritional deficiencies [59]. For example, studies of cancer-associated alterations in the enteric nervous system (ENS) reveal tumor-associated neural atrophy accompanied by loss of multiple key neuronal populations, while specific neuronal subsets may paradoxically expand [60] (see the section: *Nerve–cancer interactions in the ENS*). In addition, many solid tumors—including lung and breast cancers—are associated with immune-mediated paraneoplastic neuropathies, leading to sensory or sensorimotor peripheral nerve dysfunction in the absence of direct tumor invasion [59].

Experimental models further demonstrate that tumor-induced neuropathy can arise from inflammatory mechanisms localized to the nerve microenvironment. Implanted adenocarcinoma, fibrosarcoma, and melanoma cells growing in close proximity to peripheral nerves trigger macrophage and dendritic cell (DC)-rich perineural inflammation, microlesions in the outer nerve layers, mechanical hypersensitivity, thermal hyposensitivity, and progressive loss of nerve function. Importantly, the development of hypersensitivity is independent of direct physical contact between tumor cells and nerves and does not require PNI [61].

While extensive work on neurogenesis and tumor innervation has shown that denervation can suppress tumor growth in certain contexts (see discussion of neurogenesis and tumor innervation in the last section), other studies reveal the opposite effect. Selective denervation of vagal sensory fibers, or vagotomy, has been reported to markedly enhance lung metastasis of breast carcinoma and to promote pancreatic and gastric tumor growth with liver metastasis. Some clinical studies also indicate that patients who underwent vagotomy for gastric ulcer disease exhibit an increased risk of gastric, colorectal, biliary tract, and lung cancers [62]. These apparently contradictory findings suggest that different neural inputs coexist in a delicate functional balance within the innervated niche. Tumor-driven neurogenesis of specific nerve types may disrupt this balance such that targeted denervation can restore homeostasis in some contexts, whereas nonselective or uninformed denervation may inadvertently exacerbate tumor-promoting microenvironmental changes. Cancer-induced neuropathy itself represents another mechanism by which tumors perturb this neural equilibrium.

Overall, the impact of neuropathy on tumor biology remains complex and insufficiently explored, but emerging evidence suggests that its core consequence lies in disruption of the physiological innervated niche, as exemplified by AML-associated remodeling of the HSC niche. Tumor-induced neural injury may further promote cancer progression through multiple mechanisms, including release of damage-associated molecular patterns (DAMPs) such as neurofilament light chain (NFL), up-regulation of neurotrophic factors driving compensatory nerve regeneration and angiogenesis, and facilitation of immune evasion [60,63,64]. Consistent with this concept, recent work demonstrates that peripheral nerve injury and degeneration actively remodel the tumor-innervated niche, with axonal components released from damaged nerves, including NFL, reprograming tumor-associated macrophages (TAMs) and inducing CD8^+^ T cell senescence, thereby fostering tumor growth and immune escape [65].

Finally, therapy-induced neuropathy resulting from chemotherapy or radiotherapy warrants particular attention, as its effects on the innervated niche—and its potential contributions to tumor tolerance, persistence, and recurrence—remain largely unexplored.

#### Long-distance neural regulation—Tumor hijacking of inter-organ nerve circuits

Beyond the localized nerve–tumor interactions within the TME, emerging evidence indicates that tumors can hijack long-distance neural circuits to exert systemic influence, especially through co-opting central–peripheral neural pathways to systemically reshape immunity, metabolism, and stress responses in favor of tumor progression [15,66]. Given the rapid advancement of this field, this review aims to provide an updated and expanded framework for understanding these systemic nerve–cancer interactions, broadly classified into (a) brain–body neural circuits and (b) peripheral inter-organ neural circuits.

##### Brain–body neural circuits

Recent conceptual advances propose that tumors hijack brain–body neural circuits to disrupt physiological homeostasis at the organismal level (Fig. 1C). While this review primarily focuses on PNS mechanisms, accumulating evidence demonstrates that central neural states can be translated into direct peripheral neural outputs innervating the TME or immune organs.

Psychological stress promotes tumor initiation and progression classically through the activation of the sympathetic–adrenal medulla (SAM) and hypothalamic–pituitary–adrenal (HPA) axes, primarily through endocrine mechanisms [5,15,67]. However, beyond circulating hormones, psychological states (negative and positive) or environmental stressors can remodel tumors and immune organs via direct autonomic or sensory innervation, forming definable brain–PNS–tumor circuits. These mechanisms and neural circuits are described in detail in the sections: *Psychological states* and *Environmental temperature*; here, we provide a brief overview.

• Stress can promote tumor hyperinnervation through extracellular vesicle (EV)-mediated neuron–tumor communication, in which adrenergic suppression of ALKBH5 (AlkB homolog 5, RNA demethylase) in tumor cells alters m6A-modified RNA cargo in EVs, enhancing axonogenesis in sensory and sympathetic neurons and reinforcing a stress-driven neural circuit that accelerates PDAC growth and PNI [68].
• Psychological stress further remodels lymphatic and immune niches via sympathetic innervation, increasing lymphatic vessel density, lymph flow, and lymphogenous metastasis through β-adrenergic signaling in tumor cells, lymphatic endothelium, and macrophages [69].
• Long-range polysynaptic brain–ENS circuits provide another route for stress signals to reach tumors. In colorectal cancer (CRC), stress-responsive neurons in the lateral septum (LS) project through hypothalamic and parasympathetic nuclei to activate enteric cholinergic neurons, which innervate the TME and release neurotransmitters that directly stimulate tumor growth [70].
• Conversely, positive emotional states suppress tumor progression via central inhibition of sympathetic output. Social interaction reduces anxiety and tumor growth by engaging cortico–amygdala circuits that dampen sympathetic nerve activity and norepinephrine release within the TME, thereby enhancing antitumor immunity [71].
• Reward circuitry can similarly restrain cancer via a brain–sympathetic nervous system (SNS)–immune axis. Activation of dopaminergic neurons in the ventral tegmental area (VTA) suppresses sympathetic innervation of the bone marrow, reprograms myeloid-derived suppressor cells (MDSCs), and enhances CD8^+^ T cell-mediated tumor control [72].
• Environmental stressors such as chronic cold exposure also engage brain–PNS circuits, where persistent sympathetic activation suppresses T cell metabolism and function within the TME, thereby weakening antitumor immunity; β-adrenergic blockade reverses these effects and improves immunotherapy responsiveness [73].

Importantly, brain–body communication is bidirectional. Tumors can signal back to the CNS via peripheral nerves, reshaping central neural processing. In oral cancer, tumor-derived epidermal growth factor receptor (EGFR) ligands sensitize trigeminal neurons and, through them, amplify synaptic NMDA receptor (NMDAR) activity in the brainstem. This peripheral to central signaling increases pain and accelerates morphine tolerance by boosting glutamate release, enhancing presynaptic NMDAR activity at primary afferent terminals, and strengthening postsynaptic NMDAR responses in the trigeminal nucleus caudalis, thus establishing a pathological tumor–sensory–brain loop [74].

A 2026 study further demonstrates a fully bidirectional body–brain–body circuit to promote oncogenesis. Tumor-derived neurotrophic factors drive dense ingrowth and activation of NPY2R^+^/TRPV1^+^ vagal sensory neurons, which relay tumor-associated signals to the brainstem nucleus tractus solitarius (NTS)–rostral ventrolateral medulla (RVLM) circuit. This sensory input enhances sympathetic efferent output back to the TME, where norepinephrine acting on β2-ARs in alveolar macrophages induces an immunosuppressive ARG1^+^ phenotype that restrains CD8^+^ T cell responses. Through this sensory–sympathetic axis, tumors exploit peripheral neural circuits to suppress antitumor immunity and accelerate progression [75].

Finally, tumors may also hijack neural injury–regeneration-related pathways. A seminal study by Mauffrey et al. [46] revealed that DCX^+^ neural progenitor cells originating from the brain can migrate to the TME of prostate cancer (PCa), where they differentiate into adrenergic neurons and promote tumor growth. These progenitors, normally confined to neurogenic niches in the subventricular zone, were found circulating in the bloodstream and integrating into the TME, followed by creating an innervated niche for tumor cells. The authors proposed that tumor-induced disruption of the blood–brain barrier may permit their escape from the CNS and subsequent homing to tumors, establishing a previously unrecognized neurogenic axis connecting the brain and peripheral malignancies.

##### Peripheral inter-organ neural circuits

Beyond brain-centered pathways, tumors can also exploit direct peripheral neural circuits connecting distinct organs.

A striking example is provided by Zhang et al. [76], who demonstrated that in head and neck squamous cell carcinoma models, cancer cells under immune pressure secrete SLIT2 (slit guidance ligand 2) to activate tumor-innervating nociceptive neurons. Through an axon-reflex-like mechanism, this activation propagates to sensory fibers innervating the tumor-draining lymph node, increasing CGRP release and reprogramming the lymph node, a secondary lymphoid organ, into an immunosuppressed state. Reduced C–C motif chemokine ligand 5 (CCL5) from tumor-draining lymph node promotes M2-like TAM polarization in the TME, facilitating tumor growth and reducing immune checkpoint blockade efficacy (Fig. 1C). Importantly, targeting this inter-organ neuroimmune circuit restores antitumor immunity, directly demonstrating how tumors co-opt peripheral neural pathways linking the TME and immune organs. Additional studies support this concept by detailing how lymph nodes are innervated by sensory and sympathetic fibers, and how neural regulation within these nodes can modulate antitumor immunity and promote immunosuppression, although direct experimental evidence for bone marrow and spleen is less robust [3,77]. Scheff and Saloman [78] and Xu et al. [79] further discuss how sympathetic and sensory nerve activity can drive immunosuppressive phenotypes in immune cells both locally and systemically, and how tumors may induce nerve growth and activation to reinforce these effects.

Collectively, recent studies provide direct mechanistic evidence that tumors can not only communicate with the brain or accept brain output to induce tumor innervation [15,66] but also co-opt neural circuits connecting distant immune organs to orchestrate systemic immunosuppression, thereby reinforcing the immunosuppressive TME and promoting immune escape [3,76,77,79]. However, this research area, particularly peripheral inter-organ neural circuits, remains in its infancy, and numerous critical questions warrant investigation. Future studies should explore whether tumors similarly hijack neural regulation of bone marrow, spleen, and other immune organs beyond lymph nodes. Furthermore, it remains unknown whether cancer cells can remotely manipulate (e.g., through endocrine and EVs) neural control of metabolic organs (such as the liver) or endocrine organs (such as thyroid and adrenal glands), thereby systemically reprogramming multiple physiological systems to create a TME conducive to tumorigenesis and progression. Elucidating these potential inter-organ nerve–tumor circuits may reveal novel therapeutic vulnerabilities and expand our understanding of cancer as a systemic disease that extends far beyond the primary tumor site.

#### Tumor-specific patterns of nerve–cancer interactions

The pathological phenotypes of nerve–cancer interactions exhibit remarkable heterogeneity across different tumor types, with distinct patterns of PNI and tumor innervation that carry important implications for treatment stratification and therapeutic development (Fig. 1E).

PC and PCa represent prototypical examples where both PNI and innervation are highly prevalent. PNI occurs in up to 80% to 100% of PDAC cases and in approximately 75% of PCas, correlating with aggressive tumor behavior, increased recurrence rates, and diminished survival [9,26,80,81]. In these malignancies, nerve density nearly doubles during progression from preneoplastic lesions to invasive cancer, and is significantly correlated with tumor size, lymph node metastasis, advanced pathological staging, and reduced survival time [80]. The coexistence of extensive PNI and dense innervation in these cancers reflects their origin in richly innervated organs and suggests that neural-targeting strategies may offer particularly promising therapeutic opportunities.

In contrast, breast cancer presents a distinct pattern characterized by common innervation but rare PNI. Recent studies have revealed that peripheral nerves, particularly nerve trunks, are present in approximately 75% to 85% of breast cancers—a markedly higher proportion than previously recognized—with nerve density correlating with poor differentiation, lymph node metastasis, high clinical staging, and triple-negative subtype. Sympathetic innervation predominates in breast cancer tissues, and high nerve density in breast cancer is associated with poor patient outcome [82,83]. However, PNI occurs in only 1% to 16% of breast cancer cases (often with atypical morphology) and appears 10 times less frequently than lymphovascular invasion, with conflicting evidence regarding its prognostic significance as an independent factor [84–86]. This dissociation between high innervation and low PNI rates suggests that breast cancer progression relies more heavily on functional neural regulation through neurotransmitter signaling rather than physical nerve invasion.

Esophageal squamous cell carcinoma (ESCC) exhibits yet another pattern, with PNI being common (occurring in about 30% to 50% of cases) and serving as an independent prognostic factor for disease-free survival and overall survival [87]. However, data on active innervation and neurogenesis in ESCC remain limited compared to pancreatic or breast cancers, suggesting that ESCC progression may depend more on PNI as a metastatic route than on neurotrophic support.

These tumor-specific patterns underscore that the relative contributions of PNI versus innervation/neurogenesis vary substantially across cancer types, likely reflecting differences in organ innervation density, tumor biology, and microenvironmental characteristics. Recognition of these distinct patterns is crucial for developing tailored therapeutic approaches: Denervation strategies or neurotransmitter receptor blockade may be most effective in cancers with prominent tumor innervation (such as pancreatic and breast cancers), while interventions targeting PNI mechanisms may be more appropriate for cancers like ESCC with high PNI rates. Future precision oncology approaches should incorporate assessment of neural phenotypes to optimize treatment selection and improve patient outcomes (Fig. 1E).

### Dimension 2: Cellular components and mechanistic complexity in nerve–cancer crosstalk

#### Direct and indirect interactions in nerve–cancer crosstalk

Across numerous studies, interactions between nerves and cancer cells within the complex TME involve not only these 2 components but also various immune and stromal cell types. Understanding the cellular constituents and mechanisms of this nerve–cancer crosstalk reveals the intricate nature of the TME innervated niche and provides mechanistic insights for therapeutic targeting.

**Fig. 2.**
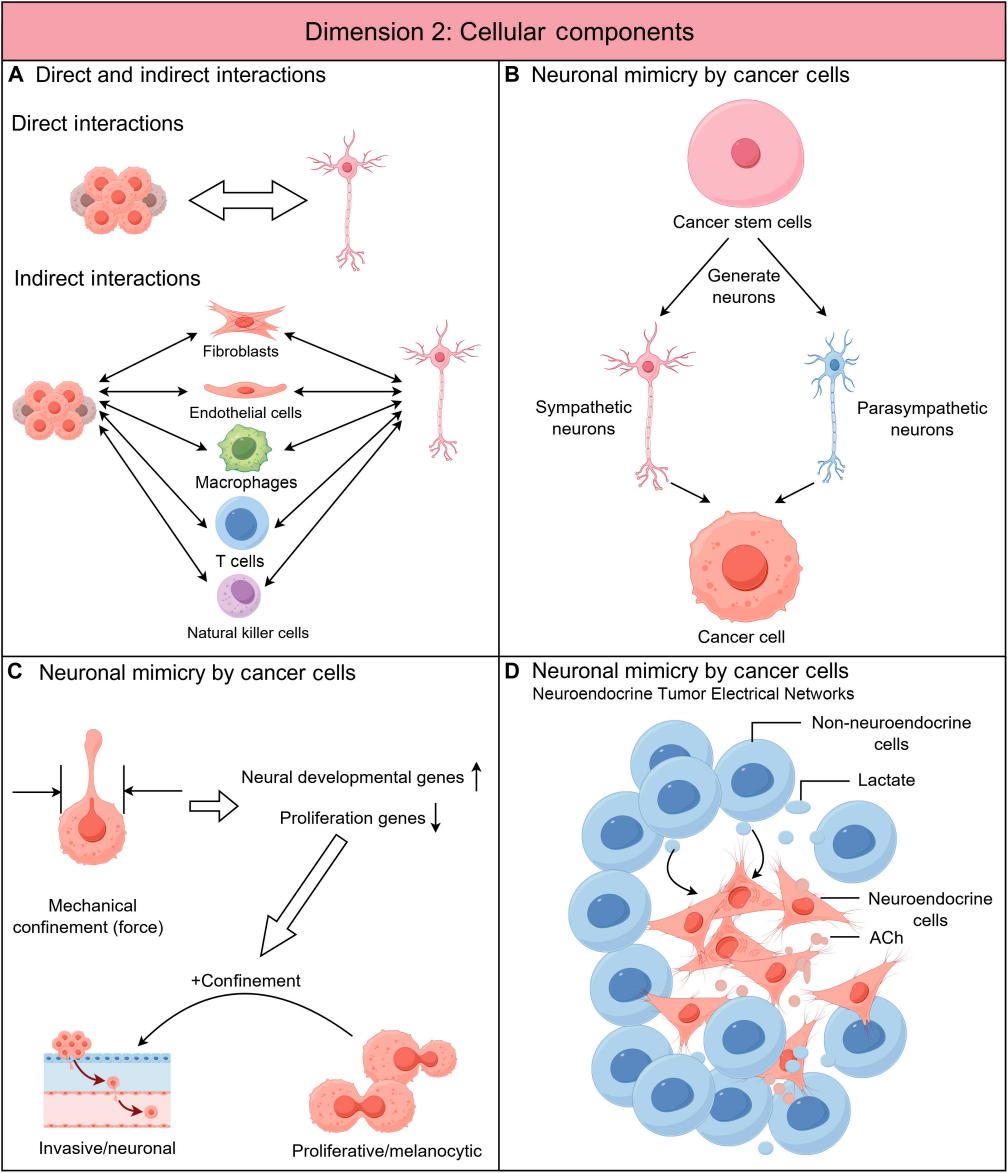
Dimension 2: Classification of nerve–cancer crosstalk based on cellular interaction mechanism. (A) Direct and indirect interactions. Nerve–cancer communication can be classified as direct or indirect depending on whether additional cell types (such as stromal, immune, or glial cells) are involved beyond nerve and tumor cells. (B) Neuronal mimicry by tumor cells. Cancer stem cells in several malignancies (e.g., gastric, colorectal, and lung adenocarcinomas) can undergo transcriptional reprogramming or transdifferentiation into sympathetic or parasympathetic-like neuronal phenotypes, thereby acquiring neural-like signaling properties and partially emulating neural functions. (C) Mechanically induced neuronal mimicry in melanoma. Mechanical forces within the melanoma TME can drive transcriptional reprogramming of tumor cells, shifting gene expression programs from a proliferative/melanocytic phenotype toward a neuronal-like and invasive state. (D) Electrically active tumor networks in small cell lung cancer arise from 2 malignant cell states. Neuroendocrine tumor cells adopt a neuron-mimic phenotype, firing spontaneous action potentials through autocrine cholinergic signaling. Their high-energy electrical activity is sustained by neighboring non-neuroendocrine tumor cells, which function as astrocyte-mimic supporters that supply lactate via a neuron–astrocyte-like metabolic coupling. This intrinsic neuro-mimetic circuit compensates for limited external innervation and drives metabolic reprogramming, intratumoral heterogeneity, and metastatic behavior. ACh, acetylcholine.

Based on whether additional cell types beyond nerves and tumor cells participate, nerve–cancer interactions can be categorized as direct (nerve–tumor only) or indirect (involving one or more intermediary cell types) (Fig. 2A). Directionality of these interactions should also be considered—that is, whether tumors act on nerves (by sending molecular signals, establishing contact, or invading neural structures) or the nerves act on tumors (by transmitting neural cues or actively innervating the tumor). In the section *PNS–tumor interactions within the TME*, we primarily focus on the nerve-to-cancer direction, summarizing the mechanisms and patterns of nerve–cancer interactions across different nervous system subtypes.

For instance, in a 2017 study on the neurogenesis/innervation phenotype, chronic stress was shown to elevate circulating systemic epinephrine, which activates β2-AR on pancreatic intraepithelial neoplasia (PanIN) and PC cells. This activation promotes tumor growth and neurotrophin secretion [e.g., NGF and brain-derived neurotrophic factor (BDNF)], which in turn stimulates sympathetic nerve sprouting into the tumor, forming a feedforward loop that amplifies local adrenergic signaling [50]. This represents a direct bidirectional interaction, where both tumor and nerves directly act on each other.

In contrast, studies by P. Frenette’s group revealed indirect interactions in innervation phenotypes. Their 2013 work demonstrated that axonogenesis of autonomic nerves within the PCa stroma plays a key role in tumor initiation and progression. Sympathetic fibers release norepinephrine that acts on β2- and β3-adrenergic receptors on stromal cells, promoting tumor cell early tumorigenesis and survival. Meanwhile, parasympathetic fibers deliver acetylcholine (ACh) that binds to muscarinic M1 receptors (M1 mAChRs) on stromal cells, enhancing tumor proliferation and dissemination to lymph nodes and distant organs [48]. A subsequent study [88] found that sympathetic nerves closely associate with tumor neovasculature during progression, with both nerve density and norepinephrine levels markedly increased in high-grade PCas. Mechanistically, endothelial β2-AR signaling reprograms endothelial metabolism toward aerobic glycolysis, triggering an angiogenic switch that fuels rapid vascularization and tumor growth—indicating that adrenergic nerves promote tumor progression indirectly through vascular endothelial cells.

In the PNI context, as most studies focus on structural invasion rather than neural regulatory function, nerve effects on tumors are generally mediated indirectly through Schwann cells, immune cells (e.g., macrophages), and cancer-associated fibroblasts (CAFs), whereas direct interactions are largely limited to tumor invasion of nerves [31,43].

A case in point is a 2025 study on PNI that demonstrated that invading cancer cells directly damage nerves and degrade the myelin sheath—representing a direct interaction. The injured neurons then activate interleukin-6 (IL-6) and type I interferon (IFN)-mediated inflammatory programs to promote repair and regeneration. During cancer progression, repeated cancer-induced nerve injury imposes chronic stress on nerves, transforming regenerative inflammation into a persistent, immunosuppressive, and exhausted TME in the PNI niche. The subsequent recruitment of chronic inflammatory immune cells by neurons constitutes an indirect interaction, further reinforcing local immune suppression and tumor immune evasion [43].

#### Neuronal mimicry by tumor cells

Certain cancer types or cancer stem cells (CSCs) can undergo transcriptional reprogramming or transdifferentiation into neuron-like phenotypes that emulate neural functions. In these contexts, although genuine neural components may be absent, large populations of tumor cells exhibiting neuronal mimicry can effectively reproduce neural regulatory functions within the TME. Consequently, blocking corresponding neuronal receptors or neurotransmitter signaling pathways presents potential therapeutic opportunities. Therefore, this review includes neuronal mimicry within the conceptual scope of cancer neuroscience, as such tumors display innervation-like phenotypes despite the absence of true nerves.

Lu et al. [89] demonstrated that CSCs derived from gastric, colorectal, and lung adenocarcinomas possess the intrinsic ability to differentiate into sympathetic- or parasympathetic-like neurons, thereby promoting cancer progression through their reciprocal interactions with tumor cells (Fig. 2B). Specifically, in xenograft mouse models, the researchers directly injected patient-derived CSCs—without any prior in vitro induction, including monoclonal CSC populations—and observed that these cells spontaneously differentiated in vivo into functional neuron-like cells of both sympathetic and parasympathetic lineages. These newly formed neuron-like cells expressed human-specific nuclear marker NuMA along with neural markers such as β3-tubulin, MAP2 (microtubule-associated protein 2), TH (tyrosine hydroxylase; a sympathetic marker), and VAChT (vesicular acetylcholine transporter; a parasympathetic marker), indicating that the TME itself has the capacity to induce neural differentiation. Furthermore, suppression of MAP2 expression markedly inhibited neuronal differentiation and slowed tumor growth, confirming that CSCs not only constitute the tumor mass but also actively remodel its neural microenvironment. This finding highlights a novel therapeutic perspective—targeting the neurogenic potential of CSCs to interfere with tumor progression.

This concept was reinforced by organoid and xenograft studies of CRC, showing that ALDH^+^ CSCs are enriched for genes governing nervous system development [90]. Functional analyses identified early growth response 2 (EGR2) as a key transcription factor linking neural differentiation and stemness maintenance via SOX2 and HOX family regulation. Targeting EGR2 impairs both CSC survival and neurogenic capacity, providing a potential strategy to eliminate CSCs and block nerve-like tumor progression.

Beyond biochemical cues, recent studies have uncovered that mechanical forces within the TME can also drive neuronal mimicry. A 2025 study revealed that mechanical confinement at tumor boundaries induces neuronal-like transcriptional programs via chromatin remodeling, switching tumor cells from a proliferative to an invasive state [91] (Fig. 2C). Interface cells exposed to compressive stress exhibited elongated nuclei and up-regulated neural-developmental genes such as SOX11, NEUROD1, NNAT, and NEFM, while proliferation markers (MITF and TYRP1) were down-regulated—indicating that physical forces can promote neurogenic reprogramming at the invasive front.

Neuroendocrine tumors provide another paradigm of neuronal mimicry, as their cell of origin already possesses partial neural traits. In 2025, a *Nature* article reported that SCLC neuroendocrine cells exhibit neuronal-like excitability, generating spontaneous action potentials and forming intratumoral electrical networks [92] (Fig. 2D). This electrical activity enhances malignancy and metastasis while reshaping metabolism toward oxidative phosphorylation dependency. Non-neuroendocrine cells metabolically support this process by secreting lactate, reminiscent of neuron–astrocyte coupling. Intriguingly, the neurotransmitter ACh—both externally derived and endogenously synthesized by SCLC cells—triggers these electrical events, suggesting a self-sustaining cholinergic loop. As external innervation diminishes during progression, tumor-intrinsic electrical signaling increases, forming a vicious cycle that drives heterogeneity and metastasis.

Neuroendocrine tumors possess intrinsic advantages that render them more prone to neuronal transdifferentiation than epithelial-derived cancers. At the molecular level, SCLC cells express multiple synaptic genes (e.g., NRXN1, NLGN1, and RELN), establishing the basis for functional connectivity with neurons. Indeed, recent studies visualized structurally complete and electrophysiologically active synapses between neurons and SCLC cells, capable of activating NMDA and GABA_A receptors [57]. In contrast, in human PDAC, neuronal axons merely form partial synapse-like junctions with cancer cells. These structures are functionally analogous yet structurally incomplete, representing a strategy by which cancer cells hijack peripheral neural signaling within their microenvironment—hence termed pseudo-synapses [56].

Further supporting the neural mimicry paradigm, a 2024 study of medullary thyroid carcinoma—a classic neuroendocrine malignancy—demonstrated that tumor cell aberrant expression and secretion of the neuropeptide CGRP disrupts DC differentiation, thereby impairing the activation of tumor-infiltrating T cells. Inhibition of the CGRP receptor [calcitonin receptor-like receptor (CALCRL)/receptor activity modifying protein 1 (RAMP1) complex] successfully reversed this effect in vitro, restoring normal DC development. Mechanistically, CGRP activates adenosine 3′,5′-monophosphate (cAMP)-related signaling and up-regulates KLF2, which together alter DC maturation and suppress T cell cytotoxicity [93].

At this point, the scope of cancer neuroscience may be further expanded. In recent years, immunological studies have uncovered that neurotransmitters are intrinsic mediators of immune signaling [94,95]. Immune cells are not merely passive recipients of neuronal input; rather, they can actively synthesize, secrete, and utilize neurotransmitters as immunomodulatory factors. This bidirectional communication constitutes a central mechanism of the neuroimmune axis [96,97]. If tumor cells or CSCs can reprogram into neuron-like cells capable of using neurotransmitter and electrophysiological signaling, they may likewise co-opt immune synapses, employing neurotransmitters to influence immune cell behavior. Exploring this direction could tightly interconnect cancer neuroscience with neuroimmunology, establishing a broader framework of tumor–nerve–immune crosstalk.

Recent findings illustrate this convergence. For instance, Best et al. [98] reported that in STK11/Lkb1-deficient KRAS-mutant lung adenocarcinoma, elevated glutamate levels within the TME correlate with enhanced CD8^+^ T cell activation and improved responsiveness to PD-1 blockade. Conversely, Xiong et al. [99] demonstrated that tumor-derived glutamate can attenuate the cytotoxic activity of neutrophils.

Collectively, these findings suggest that tumors exploit neuronal mimicry to co-opt neural functions for their own progression. In this context, inflammation and immune components—among the most critical elements of the TME—are closely intertwined with the nervous system, sharing many neurotransmitters and neuropeptides. Clearly, neural signaling and regulation play a pivotal role within the TME. Although this research field remains nascent and fragmented, it represents one of the most promising and interdisciplinary frontiers of cancer neuroscience. Future studies should aim to systematically expand this line of inquiry, thereby deepening our understanding of nerve–cancer crosstalk (Fig. 2).

### Dimension 3: Modes of communication in nerve–cancer crosstalk

#### Direct cell–cell contact

**Fig. 3.**
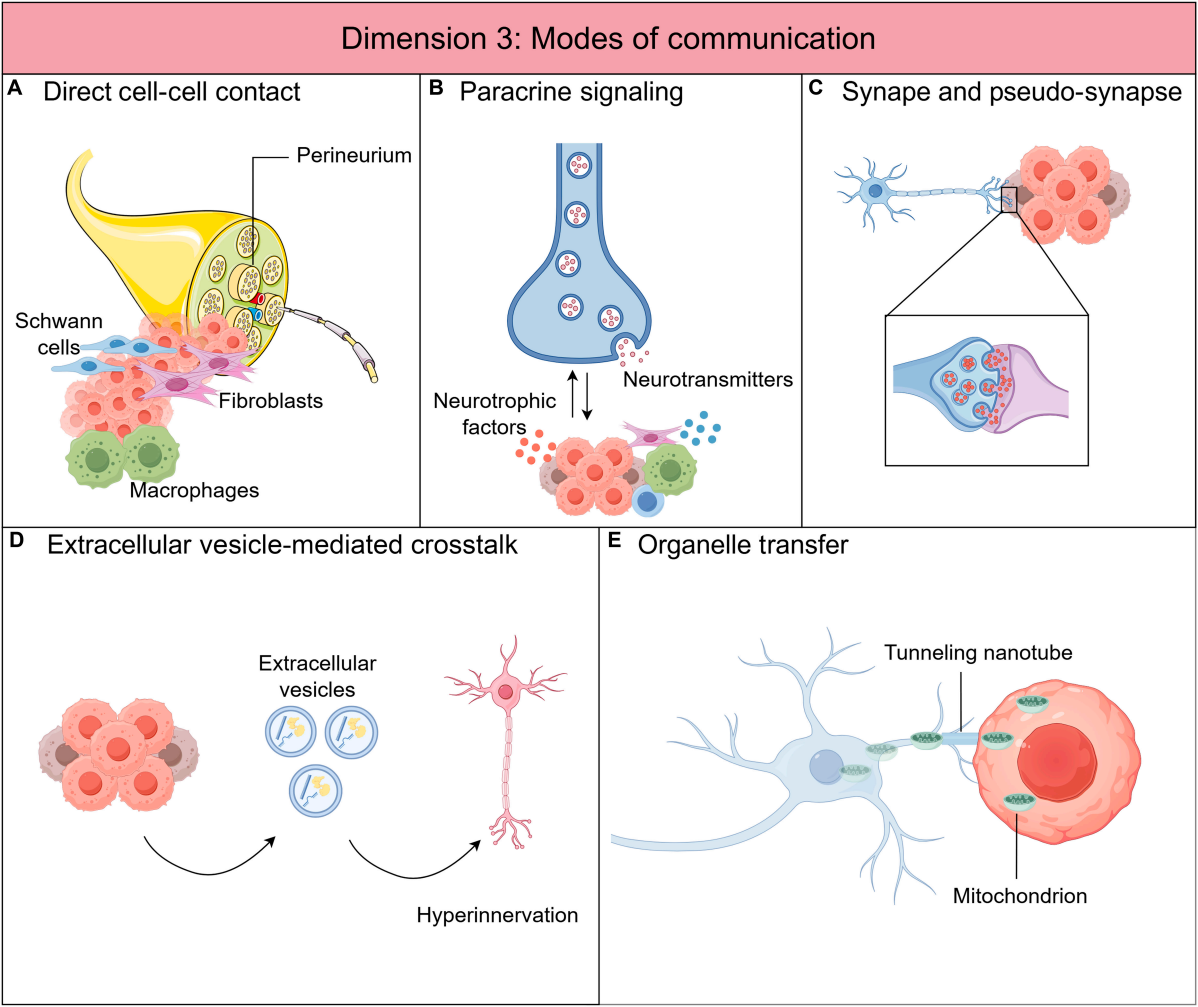
Dimension 3: Modes of communication in nerve–cancer crosstalk within the TME. (A) Direct cell–cell contact. Physical interactions occur between tumor cells, Schwann cells, and nerve fibers, enabling myelin sheath degradation and structural guidance within the TME. (B) Paracrine signaling. Paracrine ligand–receptor communication represents the most extensively studied mechanism across multiple nerve–cancer interaction phenotypes and involves diverse cellular components, including tumor cells, neurons, Schwann cells, immune cells, and stromal cells. (C) Synapse and pseudo-synapse formation. Recent studies published in 2025 have demonstrated that synaptic or synapse-like structures can form between innervating axons and tumor cells, enabling activity-dependent signaling and electrical communication. (D) Extracellular vesicle-mediated communication. In addition to secreting neurotrophic factors, tumor cells can release extracellular vesicles that carry signaling molecules capable of promoting neurogenesis and tumor innervation. (E) Organelle transfer through tunneling nanotubes (TNTs). Recent evidence indicates that neurons can directly transfer functional mitochondria to tumor cells via TNTs, thereby enhancing tumor metabolic efficiency, cellular plasticity, and metastatic potential.

Direct cell–cell contact is a hallmark of the PNI phenotype. A 2025 study meticulously detailed this process, demonstrating that cancer cells can directly degrade the myelin sheath of tumor-associated nerves, leading to nerve injury ​[43] (Fig. 3A). This direct interaction results in structural damage (evidenced by electron microscopy showing myelin debris and axonal mitochondrial abnormalities) and functional impairment (reduced electrical conduction amplitude), independent of paracrine signaling or synapse formation.

Schwann cells, the principal glial component of the peripheral nerves, are pivotal players in the direct cell–cell interaction mechanisms facilitating PNI [32] (Fig. 3A). A key mechanism involves dedifferentiated Schwann cells​ establishing direct contact with cancer cells, triggering the extension of protrusions from the latter. This invasive behavior is strictly dependent on physical contact, as soluble factors released by Schwann cells or preformed laminin tunnels were insufficient to promote equivalent invasion. Schwann cells intercalate between cancer cells, facilitating their dispersal from the primary tumor and migration toward nerves, ultimately culminating in PNI. The initiation of this process is largely attributed to Schwann cell expression of NCAM1 [26,32].

#### Paracrine signaling

Paracrine signaling represents the most extensively studied mechanism in both PNI and cancer-induced neurogenesis/innervation in the TME. As previously discussed in the context of PNI, the molecular dialogue between cancer cells and nerves (encompassing neurons/axons, Schwann cells, immune cells, and other stromal components) is mediated by a diverse array of paracrine signaling molecules. These include neurotrophins, GDNF, chemokines/cytokines, midkine family molecules, neural adhesion molecules, axon guidance molecules, and neurotransmitters/neuropeptides [9,31,32].

For instance, Schwann cells are recruited toward cancer cells, even during early neoplastic stages, primarily through the chemoattractive action of cancer-derived NGF acting on the Schwann cell receptor p75NTR [27]. Furthermore, Schwann cells promote PNI by secreting L1CAM, which not only attracts cancer cells via MAPK signaling activation but also up-regulates metalloproteinases through STAT3 activation [35].

In the context of neurogenesis/innervation, tumor cell-derived secretion of neurotrophic factors is a canonical paracrine mechanism [50]. Conversely, neurotransmitter and neuropeptide release from nerves toward cancer, immune, or stromal cells is also generally classified as paracrine unless a specialized synaptic structure is confirmed (Fig. 3B). Examples include neuronal release of neurotransmitters (e.g., norepinephrine and ACh) to modulate immune cells [76] or endothelial cells [88], a process often termed nonsynaptic neurotransmission. If evidenced by synapse-like structures, the communication is defined as synaptic or pseudo-synaptic transmission (see the next section *Synapses and pseudo-synapse formation*) [56,57].

From a neuroscientific perspective, this diffuse, nonsynaptic intercellular signaling is more precisely classified as volume transmission [including paracrine and EV-mediated communication (see the section *EV-mediated crosstalk*)], contrasting with the direct, point-to-point wiring transmission​ [e.g., synaptic communication (see the section *Synapses and pseudo-synapse formation*), gap junctions, and tunneling nanotube (TNT)-mediated communication (see the section *Organelle transfer through TNTs*)]. Volume transmission is characterized by the following: (a) Diffuse: Signal molecules diffuse from the release site to influence all receptor-bearing cells within range, without a confined anatomical pathway. (b) Low concentration: Signals are diluted in the extensive extracellular space, resulting in low effective concentrations. (c) Slow and sustained: The actions initiate slowly and persist longer, ranging from seconds to hours [100–102].

#### Synapses and pseudo-synapse formation

The most recent advances confirm the existence of genuine synaptic structures​ between neurons and cancer cells, revealing their functional activity (Fig. 3C). In gastric cancer, sensory nerves recruited into the tumor via cancer-derived NGF form synapse-like connections​ with cancer cells, establishing a bidirectional electrical circuit. Nerve-derived CGRP signaling through its receptor on cancer cells promotes tumor growth and metastasis. Disrupting this circuit with a CGRP receptor antagonist decouples the communication and suppresses tumor growth in vivo, representing the first evidence of functional synaptic communication between a peripheral tumor and nerves [55].

In PDAC, pseudo-synaptic connections​ form between sensory nerve endings and cancer cells. These sites feature a selective enrichment of the NMDAR subunit GRIN2D (NMDAR2D) on cancer cells, rendering them responsive to neuron-derived glutamate and thereby fueling tumor progression. Interestingly, neuronal activity converts a subset of cocultured PDAC cells into calcium-responsive cells via these GRIN2D-dependent signaling. Disrupting this glutamate–GRIN2D signaling at these nerve–cancer pseudo-synapses significantly improves survival in vivo, highlighting its therapeutic potential [56].

Another study in SCLC, employing electrophysiology, optogenetics, and super-resolution microscopy, identified the presence of both glutamatergic and GABAergic synaptic structures [57]. These neuron–tumor synapses were functionally active, mediating neuronal activity-dependent activation of tumoral NMDA and GABA_A receptors, thereby conferring a significant proliferation advantage to the tumor.

Taken together, these works demonstrated the formation of functional nerve–cancer pseudo-synapses or synapses, enabling fast, activity-dependent, and spatially restricted nerve–tumor communication in the innervated niche. This discovery helps bridge a long-standing gap in cancer neuroscience and expands the concept of innervation from mere proximity to authentic, functional synaptic signaling.

#### EV-mediated crosstalk

Recent studies have identified EVs as a novel mechanism for neural communication, sometimes referred to as the “roamer” type of volume transmission. This mode represents a nonsynaptic, diffuse signaling pathway [100].

EVs play a critical role in Schwann cell reprogramming during PNI. EVs derived from PDAC cells, pancreatic stellate cells, other TME components, and circulating plasma collectively induce Schwann cell dedifferentiation and promote a pro-migratory phenotype. Clinically, plasma-derived EV p75NTR expression is significantly elevated in patients with PNI and correlates with reduced overall survival, serving as an independent prognostic factor and potential therapeutic target [30]. Additionally, a 2024 study revealed the role of CAF-derived exosomes in PC neural remodeling. Specifically, the exosomal PIAT complexes with the m5C reader protein YBX1, inhibiting its ubiquitin-mediated degradation. This stabilizes YBX1 and enhances its interaction with mRNAs of pro-invasion genes (e.g., EGR1, NTRK1, and SMAD7), thereby promoting PNI [103].

This EV-mediated mechanism is also co-opted in neurogenesis/innervation phenotype (Fig. 3D). For example, tumor-derived EVs carrying specific microRNA (miRNA) cargo (e.g., increased miR-21, miR-324, and decreased miR-34a) reprogram nerves and induce neuritogenesis to facilitate tumorigenesis [104]. Similarly, EVs delivering axon guidance proteins like EphrinB1 promote sensory nerve outgrowth and tumor progression [105]. A seminal 2025 study demonstrated that psychological stress-activated sympathetic nerves release norepinephrine, which acts on PC cells via β2-AR to suppress the m6A demethylase ALKBH5. Consequently, ALKBH5 deficiency leads to hypercellular m6A-modified RNAs, which are packaged into EVs and released. Nerves then uptake these EVs, and the m6A-modified RNAs act as molecular “sponges” via SND1, ultimately up-regulating neurogenic genes and promoting tumor innervation and progression [68].

#### Organelle transfer through TNTs

A recently elucidated mode of wiring transmission is the TNT-mediated communication​, which is characterized by direct cytoplasmic continuity between spatially separated cells, allowing long-range, structured intercellular signaling and information exchange [100].

A groundbreaking 2025 study provided the first evidence of this mechanism in nerve–cancer interactions, demonstrating that neurons can directly transfer functional mitochondria to cancer cells via TNTs. This transfer of organelles markedly enhances the recipient cancer cells’ metabolic efficiency, plasticity, and, consequently, their metastatic potential [106] (Fig. 3E).

## Interactions between the Nervous System and Cancer: Multiple Patterns across the PNS

### Structural and functional divisions of the PNS

The nervous system is broadly divided into the CNS and the PNS. The CNS, consisting of the brain and spinal cord, serves as the primary integrative and command center for the entire nervous system. Interactions between cancer and the CNS primarily involve crosstalk between tumor cells, neurons, and glial cells. In contrast, the PNS encompasses all neural structures outside the CNS, including cranial and spinal nerves, and functions as the communication network between the CNS and the rest of the body [107,108]. The PNS innervates diverse and complex organs and tissues throughout the body, leading to a wide array of interaction mechanisms between nerves, tumor cells, and other TME components.

Within the PNS, the somatic nervous system mediates voluntary control of skeletal muscles via motor nerves and conveys sensory information from the periphery to the CNS through sensory nerves [108].

The autonomic nervous system (ANS), another major division of the PNS, regulates involuntary physiological functions and is subdivided into the sympathetic (SNS), parasympathetic (PSNS), and enteric (ENS) nervous systems. The sympathetic and parasympathetic branches innervate most visceral organs, maintaining homeostasis through antagonistic yet coordinated actions [109]. The ANS is tightly integrated with the CNS, with central structures such as the hypothalamus and brainstem modulating autonomic output, as emphasized by the American Spinal Injury Association [110]. The SNS is responsible for mediating the fight-or-flight response, preparing the body for action during stress or increased activity. It increases heart rate and contractility, dilates bronchioles, mobilizes energy stores, and redirects blood flow from the gastrointestinal tract and skin to skeletal muscles [110,111], whereas the PSNS governs rest-and-digest functions, promoting energy conservation and recovery. It decreases heart rate, enhances gastrointestinal motility and secretion, and supports processes such as urination and defecation [110,112]. Both systems work in concert to maintain homeostasis and adapt organ function to changing physiological demands [109,110].

Beyond these systemic and organ-level physiological roles, the SNS and PSNS also modulates tissue development, regeneration, immune regulation, angiogenesis, and tissue remodeling. The SNS exerts these functions primarily through adrenergic signaling. In tissue development and regeneration, sympathetic innervation influences stem cell behavior and wound healing by regulating keratinocyte proliferation, migration, and inflammatory cell recruitment; norepinephrine and neuropeptide Y (NPY) can either suppress or promote inflammation and re-epithelialization depending on receptor subtype and local context [113,114]. Sympathetic activity also promotes angiogenesis via vasodilatory mediators and can stimulate endothelial cell proliferation, especially in the context of injury or TMEs [113,115]. In immune regulation, sympathetic fibers innervate lymphoid organs and modulate both innate and adaptive immune responses, often suppressing Th1-mediated cellular immunity and promoting Th2/humoral responses through β2-AR signaling [116–118]. Sympathetic activation can both enhance and inhibit inflammation, depending on timing, receptor subtype, and tissue microenvironment [119,120].

The PSNS supports tissue development and regeneration by maintaining progenitor cell pools and facilitating epithelial organ repair; parasympathetic stimulation has been shown to improve regeneration in organs such as salivary glands, with neurturin-mediated protection of parasympathetic nerves enhancing epithelial recovery after injury [114,121]. In immune regulation, parasympathetic activity—primarily via the cholinergic anti-inflammatory pathway—exerts consistent anti-inflammatory effects, reducing cytokine production and limiting excessive immune activation [116,119,122]. Parasympathetic signaling also contributes to angiogenesis and tissue repair by promoting vasodilation and supporting cellular migration and proliferation in the healing process [113,121].

In summary, the SNS generally drives context-dependent pro- and anti-inflammatory responses, modulates stem cell and immune cell activity, and promotes angiogenesis, while the PSNS fosters anti-inflammatory effects, supports progenitor cell maintenance, and enhances tissue regeneration and repair.

The ENS, located within the gastrointestinal tract, represents a highly specialized division of the ANS. Unlike other branches of the PNS, the ENS contains intrinsic sensory neurons, interneurons, and motor neurons organized into complex local circuits capable of autonomous reflex activity. This independence is most evident in the small intestine and colon, where the ENS can sustain peristalsis and coordinate motility, secretion, and blood flow even when extrinsic autonomic connections are severed—a property not shared by somatic or other autonomic divisions, which typically rely on central integration for reflexes and control [108,123–125]. Beyond regulating digestive functions, the ENS also communicates bidirectionally with the CNS via sympathetic, parasympathetic, and sensory pathways, forming a critical component of the gut–brain axis, while further integrating signals from the immune system, epithelium, and microbiota [124–126].

Another distinctive feature of the ENS is its latent regenerative potential. In response to injury or pathological insults, enteric glia and neural progenitors can be activated to generate new neurons (neurogenesis, or more strictly referred to as neoneurogenesis) [127–130]. This property distinguishes the ENS from the CNS and other autonomic branches, where neuronal replacement is extremely limited [111,131,132], as well as from the somatic nervous system, which primarily exhibits axonal regeneration and regrowth [133].

The sensory nervous system, a functional division of the PNS, encompasses afferent neurons and pathways that detect and transmit external and internal stimuli to the CNS. It is not a strictly independent system, but rather overlaps substantially with both the somatic nervous system and the visceral sensory components of the ANS/ENS, and is organized into several major components:

1. Special senses: vision, hearing (auditory system), balance, smell (olfactory system), and taste (gustatory system), each with dedicated sensory organs and cranial nerves [108,134].
2. Somatosensory system: detects and transmits modalities such as touch, pain (nociception), temperature (thermoception), and proprioception from the skin, muscles, and joints to the CNS via sensory nerves, whose cell bodies are located in the dorsal root and cranial sensory ganglia, enabling perception and reflexive responses [135–137].
3. Visceral sensory system: conveys information from internal organs through cranial and spinal sensory pathways, many of which overlap with those serving the ANS. Visceral sensory fibers relay signals from baroreceptors, chemoreceptors, stretch receptors, and nociceptors, playing a critical role in homeostasis and organ function [135,138,139].
4. Enteric sensory system: intrinsic sensory neurons within the ENS detect mechanical and chemical changes within the gastrointestinal tract and communicate with both the CNS and local enteric circuits [7,108,135].

Collectively, the sensory nervous system exhibits a functional and anatomical diversity spanning somatic, autonomic (including visceral and enteric), and cranial domains of PNS [108,135,138,139]. Notably, beyond classical sensory transmission, sensory nerves also actively participate in local tissue regulation and immune modulation, reminiscent of ANS functions. For instance, neuropeptides such as CGRP and substance P (SP) released from sensory endings can modulate vascular tone, inflammation, and immune cell activity, thereby contributing to neurogenic inflammation and tissue homeostasis [135,140]. Thus, sensory nerves act both as afferent conduits for environmental and physiological signals and as local effectors within organs.

In summary, while the nervous system is conventionally categorized into central and peripheral divisions, its functional complexity extends far beyond anatomy. It exerts widespread regulation at both systemic and local (innervated niche) levels, with remarkable plasticity. Importantly, tumors have evolved to adapt to and exploit these powerful neural functions, thereby promoting their initiation and progression.

### PNS–tumor interactions within the TME

To systematically organize the rapidly expanding literature on nerve–cancer crosstalk, we apply our 3-dimensional framework to examine interactions across distinct divisions of the PNS. It is important to note that classical research on PNI has primarily focused on tumor invasion and neural injury response rather than on functional distinctions among PNS divisions. Additionally, most established PNI models are based on dorsal root ganglion (DRG) neurons and their sensory nerve fibers, with limited characterization of sympathetic (SNS) or parasympathetic (PSNS) contributions. However, recent studies have begun to incorporate division-specific characterization of peripheral nerves, including emerging efforts to define subtype preferences in PNI across different tumor types and organ contexts. Nevertheless, such investigations remain limited, and a comprehensive classification of PNI according to PNS divisions is still lacking. Therefore, in order to maintain conceptual clarity, we first analyze PNI as a distinct pathological phenotype, and subsequently examine neurogenesis and tumor innervation in the context of specific neural subtypes (sensory, sympathetic, and parasympathetic). This organizational approach enables a structured alignment with dimension 1 (pathological phenotype) of our 3-dimensional framework.

For dimension 2 (cellular components), we further dissect nerve–tumor interactions into direct and indirect interaction modes. Direct interactions occur directly between neurons/nerve fibers and tumor cells. Indirect interactions involve intermediary stromal, glial, and immune components within the TME, which mediate neural influence on tumor progression. Within each subsection, we provide a detailed mechanistic analysis of nerve–tumor interactions across multiple cancer types and organ contexts. This includes diverse modes of signal transmission—such as direct physical contact, paracrine ligand–receptor interactions, synapse-like communication, EV exchange, and TNT-mediated transfer—as well as intracellular molecular mechanisms, including signal transduction networks, transcriptional regulation, and intercellular molecular crosstalk. This integrative mechanistic dissection aligns with dimension 3 (molecular pathways) of our 3-dimensional framework.

The ENS represents the most complex division of the PNS and is therefore discussed separately in the section *Nerve–cancer interactions in the ENS*. Given its semi-autonomous regulatory capacity and its designation as the “second brain”, we first introduce ENS physiology before examining its involvement in nerve–cancer crosstalk. Although direct ENS-driven tumor interactions remain relatively limited in current literature, emerging studies have described ENS-associated PNI, cancer-induced enteric neuropathy, and the unique functions of enteric glial cells (EGCs)—whose roles differ substantially from Schwann cells in other PNS divisions. Additionally, numerous studies have investigated ENS-related neurotransmitters in gastrointestinal tumors. While these reports often lack definitive neuronal attribution, they remain highly informative for understanding potential ENS-driven tumor innervation and signaling mechanisms.

Across studies that align with our 3-dimensional framework, neural function emerges as the central determinant of tumor modulation. Neurotransmitters and neuropeptides acting on a variety of receptors represent the core functional mediators of peripheral nerve–cancer crosstalk. To facilitate comparative analysis, we summarize some representative findings in Table 1, which systematically categorizes neurotransmitter/neuropeptide types, corresponding receptors, cancer types, PNS divisions, interaction types (direct versus indirect), communication modes, functional roles, signaling pathways, and overall pro- or antitumor effects.

**Table 1.** Roles of neurotransmitters and neuropeptides in peripheral nerve–cancer crosstalk in the TME

Neurotransmitter type	Corresponding receptors	Cancer type	Type of peripheral nervous system	Direct or indirect interactions	Modes of communication	Functions in cancer	Effect	Signaling pathways	PMID
ACh	nAChR	Pancreatic ductal adenocarcinoma	PSNS	Indirect interaction	Paracrine signaling	Reducing CD8^+^ T cell infiltration and favoring Th2 over Th1 differentiation	Pro-tumor effect	HDAC1-mediated suppression of CCL5	32098780
NE	β2-AR	Pancreatic ductal adenocarcinoma	SNS	Direct interaction	Paracrine signaling	Promotes tumor growth by increasing NGF secretion and sympathetic innervation, enhancing local NE accumulation	Pro-tumor effect	β2 adrenergic–NGF feedforward loop; β2-AR-dependent signaling	29249692
NE	β2-AR	Pancreatic ductal adenocarcinoma	SNS	Direct interaction	Extracellular vesicle-mediated crosstalk	Stimulates tumor innervation and progression via ALKBH5 down-regulation and m6A RNA transfer in vesicles	Pro-tumor effect	NE–β2-AR–CREB–CHD4–H3K27ac–ALKBH5, ALKBH5-mediated m6A RNA modification and extracellular vesicle transfer	40419796
NE, ACh	β2-AR, β3-AR, M1 mAChR	Prostate cancer	SNS, PSNS	Indirect interaction	Paracrine signaling	NE promotes early tumor development; ACh promotes tumor invasion and metastasis; both promote cancer indirectly by stroma cells	Pro-tumor effect	Adrenergic signaling, cholinergic signaling	23846904
NE	β2-AR	Prostate cancer	SNS	Indirect interaction	Paracrine signaling	Activates angiogenic switch, fuels tumor growth	Pro-tumor effect	Adrenergic signaling, endothelial metabolic shift (oxidative phosphorylation)	29051371
NE	β2-AR	Lung cancer	SNS	Indirect interaction	Paracrine signaling	Promotes M2 macrophage polarization, angiogenesis, and tumor growth	Pro-tumor effect	Adrenergic signaling, cAMP–PKA–CREB	31176001
ACh	M3 mAChR	Gastric cancer	PSNS	Direct interaction	Paracrine signaling	Promotes tumorigenesis	Pro-tumor effect	ACh–NGF axis, YAP signaling	27989802
ACh	M1 mAChR	Pancreatic ductal adenocarcinoma	PSNS	Direct interaction	Paracrine signaling	Suppresses tumorigenesis and cancer stemness	Anti-tumor effect	EGFR/MAPK, PI3K/AKT signaling	30185628
CGRP	CALCRL/RAMP1 complex	Gastric cancer	Sensory nervous system	Direct interaction	Paracrine signaling; Synapses and pseudo-synapse formation	Promotes tumor growth and metastasis	Pro-tumor effect	CGRP–RAMP1 axis, calcium flux, PI3K–AKT/CaMK–Rb–E2F	39972142
Glutamate	NMDAR subunit GRIN2D (NMDAR2D)	Pancreatic ductal adenocarcinoma	Sensory nervous system	Direct interaction	Synapses and pseudo-synapse formation	Promotes tumor growth and spread	Pro-tumor effect	Glutamate–GRIN2D signaling, Ca²⁺ influx–CaMK4–CREB–EZH2–E2F–Rb	41005304
Glutamate	NMDAR , GABA_A receptor	Small cell lung cancer	Sensory nervous system	Direct interaction	Synapses and pseudo-synapse formation	Promotes tumor proliferation	Pro-tumor effect	Glutamatergic signaling, Ca²⁺ influx, postsynaptic currents	40931078
CGRP	CALCRL/RAMP1 complex	Oral squamous cell carcinoma	Sensory nervous system	Direct interaction	Paracrine signaling	Promotes tumor progression	Pro-tumor effect	ERK/YAP signaling	38886342
CGRP	CALCRL/RAMP1 complex	Melanoma	Sensory nervous system	Indirect interaction	Paracrine signaling	Induces CD8^+^ T cell exhaustion, promotes tumor growth	Pro-tumor effect	CGRP–RAMP1 axis, T cell exhaustion program	36323780
Substance P	NK1R	Breast cancer	Sensory nervous system	Direct interaction	Paracrine signaling	Promotes tumor growth, invasion, and metastasis	Pro-tumor effect	SP–NK1R, ssRNA–TLR7–PI3K–AKT axis	39112700
CGRP	CALCRL/RAMP1 complex	Pancreatic ductal adenocarcinoma	Sensory nervous system	Indirect interaction	Paracrine signaling	CGRP decreases IL-15 in CAFs, thereby suppressing NK cell infiltration and cytotoxic function; Promotes tumor progression and cancer pain	Pro-tumor effect	CGRP–CAF–NK cell axis	40122998
CGRP	CALCRL/RAMP1 complex	Oral mucosa carcinomas	Sensory nervous system	Direct interaction	Paracrine signaling	Induces cytoprotective autophagy; Promotes cancer cell survival in nutrient-poor environments	Pro-tumor effect	ROS–c-Jun–NGF–TrkA; CGRP–Rap1–mTOR axis	36395769
CGRP	CALCRL/RAMP1 complex	Oral squamous cell carcinoma	Sensory nervous system	Indirect interaction	Paracrine signaling	Promotes tumor growth by modulating tumor-infiltrating lymphocytes	Pro-tumor effect	CGRP–RAMP1 immune inhibition	35388989
CGRP	CALCRL/RAMP1 complex	Head and neck squamous cell carcinoma	Sensory nervous system	Indirect interaction	Paracrine signaling	Promotes immune suppression and tumor growth	Pro-tumor effect	ATF4–SLIT2–CGRP axis	41138728
GABA	GABA_A receptor	Colorectal cancer	Brain-body axis, PSNS, ENS	Direct interaction	Paracrine signaling	Sustaining tumor growth	Pro-tumor effect	LS–LH–SPN–ENS axis; GABA-GABA_A receptor signaling, Ca²⁺ influx–TSPAN1	40841473
5-HT	HTR1B/1D/1F	Colorectal cancer	ENS	Direct interaction	Paracrine signaling	Initiating colorectal cancer self-renewal and promoting tumorigenesis and metastasis	Pro-tumor effect	Wnt/β-catenin signaling	35550066

SNS, sympathetic; PSNS, parasympathetic; ENS, enteric nervous systems; ACh, acetylcholine; NE, norepinephrine; nAChR, nicotinic acetylcholine receptor; mAChR, muscarinic acetylcholine receptor; CGRP, calcitonin gene-related peptide; AR, adrenergic receptor; CALCRL, calcitonin receptor-like receptor; RAMP1, receptor activity-modifying protein 1; NMDAR, N-methyl-D-aspartate receptor; NK1R, neurokinin 1 receptor; HTR, 5-hydroxytryptamine receptor. Direct or indirect interaction types: Direct interaction refers to signaling between nerves and tumor cells without intermediary cell types. Indirect interaction indicates the involvement of stromal, glial, or immune intermediaries.

#### PNI associated with different nervous systems of PNS

Research on PNI has traditionally centered on tumor infiltration, nerve sheath and axonal damage, and the associated injury-induced inflammatory responses [31,43]. Within this framework, the functional roles or subtypes of nerves have rarely been the primary focus. However, insights from studies on neurogenesis and tumor-associated innervation now make it clear that nerves within the TME exert substantial biological influences. Despite this, how different nerve types involved in PNI—sensory, sympathetic, and parasympathetic fibers—differentiate in their contributions to cancer progression and patient prognosis has remained insufficiently defined.

Recent work has revealed that PNI exhibits striking neural selectivity, likely tied to tumor-induced remodeling of organ- and tissue-specific innervation. A landmark study by Perez-Pacheco et al. [141] provided the first systematic characterization of nerve-type composition in oral cancer. Their findings demonstrated that oral squamous cell carcinoma (OSCC) is dominated by sensory and mixed sensory–sympathetic fibers within the tumor core, and this neural profile strongly correlated with PNI occurrence. Conversely, mixed sympathetic–parasympathetic fibers were less abundant in the tumor mass and were negatively associated with PNI. Complementary mouse studies further identified an expansion of Calca^+^ peptidergic nociceptors, highlighting a specific sensory neuron subtype that likely contributes to worse clinical outcomes.

PNI studies frequently employ in vitro DRG–tumor coculture models, which, despite their limited ability to recapitulate in vivo nerve-type specificity, have yielded important functional insights into sensory nerve–tumor interactions. For instance, NGF, which is highly expressed in PC, activates TrkA signaling to increase miR-21-5p loading into tumor-derived exosomes. These exosomes induce a Warburg-like metabolic shift and up-regulate nociceptive TRPV1 protein expression in DRG neurons, ultimately enhancing the neural invasive capacity of PC cells [142].

In PDAC, pancreatic tumor cells infiltrate surrounding parasympathetic fibers, triggering ACh release from nerve terminals and elevating ACh levels within the TME. Increased ACh suppresses CCL5 expression through HDAC1 activation, thereby reducing CD8^+^ T cell recruitment into tumors; in parallel, ACh directly inhibits CD8^+^ T cell IFN-γ production and promotes Th2 differentiation, lowering the Th1/Th2 ratio. Together, these effects generate a profoundly immunosuppressive microenvironment that accelerates tumor progression [143]. This study highlights that PNI and tumor-associated innervation are intimately interconnected rather than independent phenomena.

Taken together, these findings underscore the need for future research to delineate the nerve-type specificity of PNI across different tumor contexts and to further dissect the functional roles of each nerve subtype. Such efforts will not only clarify the mechanistic basis of PNI but also illuminate its relationship with tumor neurogenesis and innervation—ultimately enabling a more integrated understanding of cancer neuroscience as a whole.

#### Neurogenesis and tumor innervation of the SNS

##### Direct interactions

In human salivary gland adenoid cystic carcinoma, it has been identified for the first time that there is a unique pattern of small-caliber, unorganized sympathetic nerve distribution within the TME, a characteristic not previously reported in other salivary gland tumors. Further research demonstrates that sympathetic axonogenesis plays a pivotal role in the TME of adenoid cystic carcinoma, directly driving cancer cell proliferation and tumor progression. This aberrant neurogenic process is significantly associated with the poor prognosis of patients, suggesting that the generation of sympathetic nerve axons may be a critical mechanism underlying the invasiveness and treatment resistance of adenoid cystic carcinoma [144].

Sympathetic nerves activate the β2-AR on the surface of SCLC cells through the release of norepinephrine. The activation of β2-AR subsequently triggers the β2-AR–protein kinase A (PKA)–cAMP-response element-binding protein (CREB) signaling pathway, thereby promoting the proliferation of SCLC cells both in vitro and in vivo. Research indicates that the growth of SCLC can be markedly inhibited through direct ablation of the sympathetic nerve fibers, or by inhibition of the β2-AR–PKA–CREB axis with genetic or pharmaceutical approaches, highlighting a new potential target for the treatment of this highly malignant cancer [145].

Additionally, the sympathetic nerves are enriched in Epstein–Barr virus-positive diffuse large B cell lymphoma (EBV^+^ DLBCL) and activate the β2-AR of cancer cells. Transcriptome analysis reveals the up-regulation of axonogenesis-related genes and pathways in EBV^+^ DLBCL tumors. The β2-AR activation further initiates downstream signaling pathways that promote the cancer cell proliferation and tumor growth of EBV^+^ DLBCL, which are attenuated by selective β2-AR blockers [146].

In PDAC, norepinephrine released by the sympathetic nerve activates the β2-AR on cancer cells, facilitating the cancer development and the secretion of neurotrophin NGF, which further recruits more sympathetic innervation and stimulates the release of additional norepinephrine, ultimately accelerating tumor progression and establishing a positive feedback loop. Inhibition of the β2-AR or Trk (neurotrophin receptor) can disrupt this circuit, improve gemcitabine’s therapeutic effect, and prolong survival [50].

In another study, it was demonstrated that nociception and psychological stress drives PDAC progression by activating the SNS, stimulating tumor sympathetic innervation, and leading to the release of norepinephrine. Mechanistically, norepinephrine signaling subsequently inhibits the expression of RNA demethylase alkB homolog 5 (Alkbh5) in cancer cells, resulting in an aberrant N6-methyladenosine (m6A) modification of RNAs. These modified RNAs are then taken up by nerves in the TME in the form of EVs, which promote nerve growth and ultimately accelerate the progression of PC [68].

These studies collectively demonstrate that the sympathetic nerve can directly act on cancer cells by releasing neurotransmitters such as norepinephrine, manipulating the latter to promote tumor growth and progression.

##### Indirect interactions

Departing from direct interaction models, pioneering research by P. Frenette’s group identified the tumor stroma as a critical mediator of neural influence on PCa. Specifically, sympathetic nerve-derived norepinephrine targets β2- and β3-ARs on stromal components to facilitate early-stage tumorigenesis and cell survival. In parallel, parasympathetic fibers release ACh, which interacts with stromal M1 mAChRs to accelerate tumor cell proliferation and orchestrate metastatic spread to lymph nodes and distant organs [48]. Their subsequent study [88] found that sympathetic nerves closely associate with tumor neovasculature during progression, with both nerve density and norepinephrine levels markedly increased in high-grade PCas. Mechanistically, endothelial β2-AR signaling reprograms endothelial metabolism toward aerobic glycolysis, triggering an angiogenic switch that fuels rapid vascularization and tumor growth—indicating that adrenergic nerves promote tumor progression indirectly through vascular endothelial cells.

Sympathetic nerves can also indirectly affect cancer progression by manipulating tumor immune microenvironment. In PDAC, sympathetic nerves influence the TME through their axonal branches, inhibiting the accumulation of CD163^+^ macrophages within the tumor. This inhibition of M2 macrophage infiltration effectively suppresses the malignant progression of tumors [147]. Additionally, sympathetic nerves play a crucial role in regulating the function of CD8^+^ T cells via β1-ARs, directly inducing a state of exhaustion in T cells [148]. The underlying mechanisms involve catecholamines released by sympathetic nerves binding to β1-ARs on T cell surfaces, which inhibits cell proliferation and the production of their cytokines, thereby diminishing the antitumor immune response. Furthermore, exhausted CD8^+^ T cells tend to accumulate around sympathetic nerves in a β1-AR-dependent manner, further exacerbating their functional inhibition.

Norepinephrine can also induce the secretion of NGF from CAFs through the activation of their β2-AR. NGF subsequently enhances sympathetic nerve innervation and norepinephrine accumulation within the innervated niche, thereby establishing a self-reinforcing positive feedback loop. As a result, norepinephrine mediates the activation of YES-associated protein (YAP) via the α2A-AR and its downstream Gi protein signaling pathway, thus accelerating the growth of CRC. Meanwhile, NGF from CAFs also enhances CRC cell growth via the phosphatidylinositol 3-kinase (PI3K)/AKT pathway [149]. In a murine lung cancer model, catecholamines released under the control of the sympathetic nervous system promoted tumor neovascularization by driving TAMs toward an M2 phenotype via adrenergic signaling; chemical depletion of catecholamines or β-adrenergic blockade reduced M2 TAM function, attenuated angiogenesis, and restrained tumor growth while also shifting the immune microenvironment away from an immunosuppressive state [150].

These studies collectively elucidate the intricate indirect SNS–cancer interactions by manipulating the TME components in the innervated niche by releasing neurotransmitters such as norepinephrine, thus promoting tumor growth and progression, which reveal new potential targets for cancer treatment.

#### Neurogenesis and tumor innervation of the PSNS

##### Direct interactions

In 2014, Zhao et al. [151] discovered that vagus innervation promotes gastric tumorigenesis through an M3 mAChR-mediated Wnt signaling pathway. However, vagus denervation not only reduces tumor incidence and progression but also enhances the therapeutic effects of systemic chemotherapy and prolonged survival, suggesting that denervation may represent a feasible strategy for controlling gastric cancer. Subsequently, in 2017, Renz and colleagues [52] (the same group) found that cholinergic stimulation with ACh from Dclk1^+^ tuft cells and parasympathetic nerves induces the expression of NGF in gastric epithelial cells, which further enhances innervation and tumorigenesis, thereby establishing a positive feedback loop. Blocking the NGF/Trk signaling pathway or knocking out Dclk1^+^ cells markedly inhibits carcinogenesis and tumor growth in a M3 mAChR-dependent manner, in part via YAP signaling suppression, thus indicating that cholinergic signaling plays a crucial role in the onset of gastric cancer.

However, cholinergic stimulation and signaling inhibit the tumorigenesis, growth, progression, and liver metastasis of PDAC, as well as significantly extended survival. Specifically, enhanced cholinergic signaling effectively obstructs tumor occurrence and progression partly by directly activating PDAC M1 mAChR, which inhibits the MAPK/EGFR and PI3K/AKT signaling pathways, leading to a suppression of the CSC compartment [152], highlighting the divergent functional outcomes of cholinergic signaling mediated by distinct receptor subtypes.

##### Indirect interactions

Extending the above work from Renz et al. [152], they also demonstrated that enhanced cholinergic signaling decreases CD11b^+^ myeloid cells and macrophages in the TME and lowers systemic and splenic tumor necrosis factor-α (TNF-α) levels, indicating that cholinergic pathways may indirectly restrain PDAC progression by modulating both the local TME and systemic immune responses.

The vagus innervation can also inhibit the expansion of MDSCs (expanded in the spleen during carcinogenesis) through the CXCR4 signaling pathway by regulating the release of the anti-inflammatory peptide TFF2 from memory T cells. Splenic denervation can disrupt this anti-inflammatory neural reflex arc, resulting in abnormal expansion of MDSCs and suppression of CD8^+^ T cells, and thereby promoting the progression of CRC [153].

These findings suggest that the PSNS not only directly regulates tumor cell behavior through cholinergic signaling but also indirectly influences tumor progression by modulating immune cells and inflammatory responses within the TME innervated niche. Consequently, targeting the parasympathetic nerve and cholinergic signaling pathway may represent an effective strategy for inhibiting tumor growth and enhancing patient outcomes.

#### Nerve–cancer interactions in the ENS

As briefly introduced above, the ENS stands as a distinct and highly autonomous branch of the PNS. Its complexity, characterized by a vast repertoire of neurons, glial cells, and neurotransmitters, markedly exceeds that of other PNS divisions. This intrinsic complexity, coupled with the challenges in distinguishing neuronal from non-neuronal sources of signaling molecules, may explain why specific ENS–cancer cell communication has not been fully characterized in CRC or other gastrointestinal malignancies to date. Nevertheless, ENS neuroimmunology has advanced rapidly in recent years. In these contexts, enteric neurotransmitters and neuropeptides also function as immunomodulators—acting as pro- or anti-inflammatory signals that influence cytokine release, intestinal permeability, and both immune and neuronal behavior [154,155]. These emerging insights suggest that ENS–tumor interactions represent a critical area for discovery.

The mammalian digestive tract receives dual innervation: (a) extrinsic pathways, including sympathetic, parasympathetic (vagal and pelvic) efferents, and visceral afferents (vagal and spinal/DRG) with cell bodies outside the gut wall, as well as (b) intrinsic innervation via the ENS—a semi-autonomous network embedded within the intestinal wall [126,156]. Extrinsic fibers modulate ENS function by synapsing onto ENS neurons (e.g., vagal and sympathetic inputs to the myenteric plexus) or directly targeting tissues such as blood vessels and sphincters (e.g., sympathetic efferents), establishing bidirectional CNS–ENS communication essential for homeostasis, motility, and secretion [156,157].

The ENS contains approximately 500 million neurons—exceeding any other PNS division—organized into interconnected plexuses [158]: (a) myenteric plexus (Auerbach’s): located between longitudinal and circular muscle layers throughout the gastrointestinal tract, primarily regulating peristalsis and motility; (b) submucosal plexus: predominantly in the small and large intestines, supporting secretion, blood flow, immunity, and epithelial functions [60,126,159–161]. In humans, it comprises inner (Meissner’s), intermediate, and outer (Schabadasch’s) layers [159].

Functional neuronal subtypes include excitatory and inhibitory motor neurons, secretomotor neurons, vasodilator neurons, as well as intrinsic primary afferent neurons (IPANs) that detect mechanical and chemical stimuli to initiate local reflexes [156,162]. These neurons are interconnected by interneurons, forming complex reflex circuits that coordinate motility, secretion, and vascular regulation. Importantly, these circuits are also directionally organized, with IPANs providing afferent input to ascending (orally directed) and descending (anally directed) interneurons, which in turn activate appropriate efferent motor and secretomotor pathways. Notably, co-transmission is a common feature of enteric neurons, as individual neurons can synthesize and release multiple neurotransmitters or neuropeptides; for example, inhibitory motor neurons can be nitrergic (NO), VIPergic (VIP), and purinergic (ATP). This multi-transmitter capability substantially increases the functional and experimental complexity of ENS signaling networks.

Beyond neurons, the ENS uniquely contains EGCs, which are distributed not only within the myenteric and submucosal plexuses but also throughout the lamina propria and muscularis propria [156,163,164]. Unlike other PNS divisions, the ENS is composed predominantly of unmyelinated fibers, and EGCs provide specialized support functions distinct from those of Schwann cells. The remarkable transcriptomic heterogeneity of both enteric neurons and EGCs adds another layer of complexity, making it challenging to dissect nerve–tumor crosstalk at cellular resolution [156,165].

Therefore, investigating the neuronal roles and functions on intestinal TME requires comprehensive examination of contributions of extrinsic pathways, the diverse and plasticity of neuronal subtypes (and neurotransmitters/neuropeptides) within the complex intrinsic ENS, and EGCs—including their respective innervation patterns, neural circuits, and the anatomical locations and functional roles of the involved ganglia.

Notably, the complexity of the ENS makes ENS nerve–cancer interactions fundamentally distinct from those involving sympathetic, parasympathetic, or sensory nerves. Moreover, ENS-like intrinsic neural structures are not restricted to the gut wall: Intrapancreatic ganglia and ganglionated plexuses in the gallbladder and biliary system share structural and neurochemical features with the ENS, although with more limited autonomy [124,166]. These ganglia also receive direct neural inputs from enteric circuits and are therefore sometimes regarded as peripheral extensions of the gut wall ENS [124,167,168]. This raises the possibility that intrinsic neural circuits may also modulate tumor biology in pancreatic and hepatobiliary tissues, although this area remains underexplored.

##### Impact of cancer on the ENS

Structural plasticity with neuropathy of the ENS is a hallmark of colorectal carcinogenesis, characterized by extensive alterations within submucosal and myenteric plexuses at the tumor’s invasive front [169]. Microscopic and ultrastructural analyses reveal a predominant pattern of neuronal depletion: Myenteric plexuses adjacent to the invasive zone are significantly smaller with fewer neurons expressing key neuropeptides—including vasoactive intestinal peptide (VIP), pituitary adenylate cyclase-activating polypeptide (PACAP), NPY, somatostatin (SST), SP, and CGRP—compared to distal sites [60,170–174]. This atrophy, accompanied by extracellular matrix expansion and sporadic neuronal apoptosis, appears to result from physical displacement by infiltrating cancer cells [173,174]. While plexus area generally decreases with advancing tumor grade, neural components outside plexuses may paradoxically increase in higher-grade tumors [175].

In contrast, specific neuronal subsets expand near tumors: Galanin (GAL)-containing neurons in the myenteric plexus and cocaine and amphetamine-regulated transcript peptide (CART)-expressing neurons in the inner and outer submucosal plexuses can increase significantly at the tumor margin [176,177], suggesting selective neurochemical responses to the TME. Compellingly, Chagas disease—which causes severe ENS degeneration and megacolon—provides a clinical counter-argument: despite chronic constipation (a CRC risk factor), Chagas patients exhibit markedly lower CRC risk, with zero neoplasia cases found in a 50-year study of 894 megacolon resections [90,91]. This “Chagas paradox” suggests that profound intrinsic denervation may deprive tumors of essential neural trophic support, thereby preventing oncogenesis [178–180].

##### PNI of ENS

PNI can be one of the causes to induce ENS atrophy, and it occurs in approximately 20% of CRC patients and serves as a prognostic marker for tumor aggressiveness and survival [181,182]. PNI correlates with poorly differentiated tumors, tumor budding, T stage, regional lymph node metastasis, and lymphovascular invasion [181]. It also informs clinical decisions regarding adjuvant chemotherapy in CRC [183]. Notably, the incidence of PNI is higher in rectal cancer than in colon cancer, likely attributable to the rectum’s denser extrinsic innervation and/or region-specific patterns of innervation [181,182,184].

The molecular mechanisms underlying PNI in the intestine remain poorly understood and debated. A critical unresolved question is whether intestinal PNI occurs primarily via extrinsic nerve bundles or involves the ENS itself. While some propose that PNI arises through extrinsic nerve bundles from the PNS [185], others demonstrate physical interactions between enteric neurons and CRC cells. However, the specific mechanisms underlying ENS-associated PNI require dedicated investigation, as the ENS differs fundamentally from other PNS divisions in structure and composition—comprising unmyelinated fibers supported by EGCs rather than myelinated fibers with Schwann cells (a key player in PNI as discussed in the section *PNI—Cancer mimicry of nerve injury response in the TME*). Current studies reveal that enteric neurons express N-cadherin and L1CAM, which mediate CRC cell adhesion and migration. In vitro experiments by Duchalais et al. [186] demonstrated that human primary colonic tumor epithelial cells (TECs) and CRC cell lines preferentially adhere to ENS structures with greater affinity than to mesenchymal cells, involving direct interactions with enteric neurons. Blocking N-cadherin and L1CAM reduced—but did not completely abolish—TEC migration along ENS structures, indicating that enteric neuronal networks guide tumor cell migration, while additional unidentified factors contribute to this process. Further investigation is warranted to fully elucidate the molecular basis of ENS–tumor interactions in PNI.

##### Direct interactions: ENS innervation of tumor

Recent studies have begun to define the direct functional innervation of tumors by the ENS. Li et al. [70] discovered that CRC cells exploit a brain–gut polysynaptic circuit in which GABAergic neurons in the LS relay through the lateral hypothalamus (LH) and the sacral parasympathetic nucleus (SPN) to activate enteric cholinergic neurons. These enteric neurons project fibers into the TME and release GABA, stimulating ε subunit-containing GABA_A receptors on tumor cells to promote growth. Chronic stress further amplifies activity along this LS–LH–SPN–ENS axis, accelerating tumor progression [70].

Zhu et al. [187] demonstrated that enteric serotonergic neurons produce 5-hydroxytryptamine (5-HT, or serotonin), which initiates CRC stem cell self-renewal and tumorigenesis via activation of HTR1B/1D/1F receptors and Wnt/β-catenin signaling. This process is fueled by the CRC-enriched microbial metabolite isovalerate (IVA), which up-regulates neuronal 5-HT production by suppressing the NuRD complex on the Tph2 promoter. Conversely, pharmacological blockade of 5-HT signaling exhibits marked therapeutic efficacy in inhibiting CSC self-renewal [187]. Notably, while the vast majority of intestinal 5-HT is of mucosal origin, produced by enterochromaffin (EC) cells via tryptophan hydroxylase 1 (TPH1), the neuronal 5-HT discussed here represents only a minor fraction and originates from ENS serotonergic neurons—classically described as a small population of descending myenteric interneurons—via TPH2 [188]. Furthermore, distinct 5-HT receptor subtypes mediate fundamentally different biological outcomes depending on cellular context, ranging from presynaptic neuromodulatory mechanisms that are aberrantly hijacked by tumor cells to drive stemness programs, to circuit-level relay signaling that activates downstream enteric pathways under physiological conditions. In the context of mucosal proliferation and repair—processes that may, under certain conditions, be linked to carcinogenesis—neuronal 5-HT acts as a neuromodulator, activating 5-HT_2A_ (HTR2A) receptors on cholinergic neurons within the submucosal plexus, which subsequently drive muscarinic signaling to modulate epithelial proliferation and mucosal growth [189].

In addition to classical neurotransmitters and neuropeptides, recent evidence reveals that ENS neurons secrete other neuronal factors capable of promoting tumorigenesis. Vaes et al. [190] demonstrated that loss of enteric neuronal N-Myc downstream-regulated gene 4 (Ndrg4) promotes CRC through increased release of extracellular matrix proteins Nidogen-1 and Fibulin-2, which enhance CRC cell proliferation and migration.

##### Potential direct and indirect interactions: ENS signaling molecules and their impact on tumor and the TME

Although direct studies of functional ENS tumor innervation remain limited (described above), extensive research on ENS-related signaling mediators (encompassing classical neurotransmitters, neuropeptides, and neurotrophic factors)—acting either directly on cancer cells or indirectly via other TME cell types—provides critical insights into potential ENS–cancer interactions [60,156,191–193]. Since the ENS origin of many of these signaling mediators cannot be directly confirmed in the studies reviewed, we discuss their potential tumor-modulatory roles (with pro-tumorigenic and antitumorigenic effects indicated in red and blue, respectively) in Supplementary File 1, organized by physiological functional categories: (a) excitatory motor and secretomotor signaling, (b) inhibitory motor signaling, (c) diffuse neuromodulatory and sensory-associated signaling, (d) the endogenous opioid system, and (e) neurotrophic factors.

Across these categories, the reviewed molecules display divergent and often context-dependent effects on gastrointestinal tumorigenesis (Supplementary File 1). Here is a brief summary:

Among classical excitatory transmitters, ACh and SP predominantly exhibit pro-tumorigenic effects in CRC, promoting proliferation, invasion, angiogenesis, and immunosuppressive remodeling of the TME. Serotonin (5-HT), while traditionally viewed as pro-tumorigenic through receptor-mediated signaling, serotonylation, and immune modulation, demonstrates marked context dependency, with both tumor-promoting and tumor-restraining effects depending on concentration, anatomical compartment (systemic versus intratumoral), cellular source, and receptor subtype engagement.

Within inhibitory motor pathways, VIP, PACAP, nitric oxide (NO), GABA, and purinergic ATP signaling display heterogeneous effects. VIP and ATP signaling are largely associated with tumor growth, immune modulation, and metastasis, whereas PACAP and GABA show dual or context-dependent roles. NO signaling is particularly complex, with inducible nitric oxide synthase (iNOS)-derived NO strongly linked to cancer stemness and progression, while the specific contribution of ENS-derived nNOS^+^ neurons remains unresolved.

Among diffuse neuromodulators and sensory-associated peptides, CGRP, SST, dopamine (DA), and gastrin-releasing peptide (GRP) predominantly exhibit tumor-suppressive properties in most reported settings. In contrast, neuromedin U (NMU) is associated with tumor progression, metastasis, and therapy resistance. NPY and GAL demonstrate bidirectional effects depending on experimental context and disease stage. Evidence for peptide histidine isoleucine (PHI) remains limited but suggests potential pro-proliferative effects in experimental models.

The endogenous opioid system, particularly methionine-enkephalin (Met-ENK), exhibits dual and source-dependent functions, with antitumor immune remodeling and growth-inhibitory effects observed in some contexts, yet tumor-derived opioid signaling contributing to immune evasion in others.

Finally, neurotrophic factors including NGF and BDNF are predominantly associated with tumor growth, neural expansion, metastasis, and resistance to apoptosis, although their cellular origin within the TME—whether neuronal, epithelial, glial, or immune—remains to be precisely defined.

It should be noted upfront that, although these molecules are likely produced at least in part by the ENS, many of the studies discussed do not explicitly characterize the cellular source of the signaling molecule in question, or instead involve molecules derived from non-ENS components of the gut—such as neuroendocrine cells, tumor cells, or immune cells. Nevertheless, the phenotypic and mechanistic insights from these studies remain highly informative for defining future directions in ENS–cancer interaction research. When interpreting these findings, it is essential to distinguish neuronal sources (ENS versus extrinsic innervation) from non-neuronal sources (neuroendocrine cells, tumor cells, immune cells, etc.), as many signaling molecules can be produced by multiple cell types.

##### EGCs interact with cancer

Glial cells and neurons are inseparable in development, physiological function, and pathological processes—as exemplified by the critical role of Schwann cells in PNI and of glial cells in glioma. However, knowledge regarding the direct effects of EGCs on gastrointestinal carcinogenesis remains limited.

Initial pathological evidence comes from histological studies showing EGC infiltration into human CRC tumors, identified by S100B and glial fibrillary acidic protein (GFAP) staining [194]. Analysis of colonic biopsies across healthy subjects, ulcerative colitis patients, and CRC patients further revealed that EGC-derived S100B is progressively up-regulated toward malignancy, correlating with activation of the receptor for advanced glycation end-products (RAGE)–p38 MAPK–nuclear factor κB (NF-κB) pathway and a pro-inflammatory, pro-angiogenic, and anti-apoptotic microenvironment [195].

The functional pro-tumorigenic role of EGCs was directly demonstrated by Valès et al. [194], who showed that EGCs preactivated by tumor-derived soluble factors stimulate CSC expansion and tumorigenicity via paracrine prostaglandin E2 (PGE2)–prostaglandin E receptor 4 (EP4)–EGFR–extracellular signal-regulated kinase 1/2 (ERK1/2) signaling, with IL-1 from TECs identified as a critical upstream activator. This bidirectional crosstalk was confirmed in vivo, where co-injection of CRC cells with EGCs yielded significantly larger tumors. Complementarily, Yuan et al. [196] demonstrated that depletion of GFAP^+^ EGCs profoundly reduced tumor burden at early premalignant stages in both the AOM/DSS (azoxymethane/dextran sodium sulfate, chemically induced colitis-associated CRC) and Apc*^Min/+^* (familial adenomatous polyposis) models, with no effect on established tumors—suggesting that EGCs specifically promote tumor initiation rather than progression. Notably, this protective effect was independent of modulation of antitumor immunity or intestinal inflammation, pointing to alternative pro-tumorigenic mechanisms.

Beyond direct tumor interactions, van Baarle et al. [197] revealed that EGCs also reshape the TME indirectly through neuroimmune crosstalk: Tumor-infiltrating monocytes drive a reactive EGC phenotype via IL-1R signaling, whereupon EGCs reciprocally promote monocyte differentiation toward pro-tumorigenic SPP1^+^ TAMs via IL-6, with EGC abundance correlating with worse clinical outcomes.

These findings are, however, complicated by contradictory histological observations. Based on GFAP immunohistochemistry, EGC density was found to be highest in well-differentiated CRC tumors and progressively decreased in moderately to poorly differentiated tumors [156,198]. Further analysis revealed that in moderately to poorly differentiated tumors, higher GFAP^+^ EGC density was significantly associated with reduced tumor cell proliferation and decreased intratumoral leukocyte infiltration, whereas no such correlations were observed in well-differentiated tumors [198], suggesting that EGC–tumor relationships may be grade-dependent.

Given the well-established importance of glial cells in non-ENS nerve–cancer interactions, the still limited and sometimes contradictory evidence on EGC–tumor crosstalk warrants further investigation. Key questions include how EGCs interact directly with tumor cells, how they cooperate with enteric neurons, and how they indirectly modulate the TME. The molecular mediators released by EGCs and the signals that activate and recruit them to the tumor site also remain to be systematically explored [156].

Beyond the cellular and molecular components of the ENS discussed above, increasing attention has been directed toward the nerve–microbiota–immune axis as a higher-order framework for understanding ENS–cancer crosstalk. Within this axis, gut microbiota and their metabolites can directly modulate ENS activity, as exemplified by microbiota-derived IVA, which up-regulates serotonin production in enteric serotonergic neurons and promotes CRC stem cell self-renewal and tumorigenesis [187]. Conversely, the ENS also regulates the gut microbiota and indirectly influence tumor progression. Through neurotransmitters and neuropeptides, the ENS shapes intestinal motility, barrier integrity, and the luminal microenvironment, thereby influencing microbial composition and function. Notably, gut-innervating TRPV1^+^ nociceptors can also directly shape the intestinal microbiota by sustaining protective Gram^+^ Clostridium species and restraining inflammatory taxa through the neuropeptide SP, thereby maintaining a microbial ecosystem that limits inflammation and promotes tissue protection during intestinal injury [199]. Gut microbiota and their metabolites can directly act on tumor cells or modulate both mucosal and systemic immunity to affect cancer progression [200].

Together, these interconnected pathways underscore the neuro–microbiota–immune axis as a dynamic, bidirectional, and multilevel regulatory network in gastrointestinal cancer. Viewing ENS–cancer interactions through this integrated lens may help reconcile context-dependent effects of neural signaling and identify new opportunities for therapeutic intervention.

#### Neurogenesis and tumor innervation of sensory nerves

##### Direct interactions

In breast cancer, the enhanced innervation of sensory nerves is driven by axon guidance molecule SLIT2 produced by tumor vasculature. Breast cancer cells then induce spontaneous calcium activity in sensory neurons and promote the release of the neuropeptide SP, which directly interacts with the neurokinin 1 receptor (NK1R, or tachykinin receptor 1, TACR1) on cancer cells, leading to the death of some cancer cells and the subsequent release of single-stranded RNA. This RNA activates neighboring tumoral Toll-like receptor 7 (TLR7) to initiate a prometastatic gene expression program (including PI3K-AKT signaling) noncanonically, thereby accelerating tumor invasion, metastasis, and reducing survival outcomes. Clinical data indicate that patients with elevated primary tumor sensory innervation and SP levels demonstrate a greater propensity for lymph node metastasis. Aprepitan, a TACR1 antagonist, has been shown to effectively inhibit the growth and metastasis of breast cancer, offering a novel therapeutic approach for clinical treatment [201].

Functional intratumoral neuronal circuits have been demonstrated in multiple peripheral solid tumors, including head-and-neck squamous cell carcinoma (HNSCC) and high-grade serous ovarian carcinoma (HGSOC), where they promote disease progression [202]. Particularly, these tumors exhibit markedly enhanced evoked electrical activity on microelectrode array recordings—malignant tissues display far stronger evoked responses than benign or normal counterparts, and the magnitude of electrical activity correlates with the density of neural infiltration. This activity is largely driven by TRPV1^+^ nociceptor neurons, whose intratumoral innervation enables the release of the neuropeptide SP; in turn, SP directly engages its receptor NK1R on tumor cells to promote proliferation, migration, and ERK pathway activation, thereby accelerating tumor growth and metastasis. Ablation of TRPV1 neurons or pharmacologic blockade of NK1R attenuates tumor progression, highlighting the nerve–cancer electro-chemical signaling axis as a potential therapeutic target.

As mentioned in the section *Synapses and pseudo-synapse formation*, sensory nerves are now confirmed to directly form synapses or pseudo-synapses with cancer cells. In gastric cancer, sensory nerves infiltrate the TME due to the tumor cell overexpression of NGF, establishing synaptic connections with tumor cells and forming bidirectional electrical circuits. These sensory nerves facilitate the growth and metastasis of gastric cancer by releasing CGRP and binding to its up-regulated receptor complex CALCRL/RAMP1 on gastric cancer cells, thereby activating the PI3K–AKT and CaMK pathways that converge on Rb–E2F signaling. The administration of CGRP receptor antagonists can disrupt the neuron–tumor communication loop, thereby inhibiting tumor growth and prolonging survival in mouse models [55]. In PDAC, pseudo-synaptic connections form between sensory nerve endings and cancer cells. These connections exhibit a selective enrichment of the NMDAR subunit GRIN2D on cancer cells, which renders them responsive to neuron-derived glutamate, thereby inducing tumor progression. Notably, neuronal activity converts a subset of PDAC cells into calcium-responsive cells through these GRIN2D signaling. Disruption of the glutamate–GRIN2D signaling at these nerve–cancer pseudo-synapses markedly enhances survival in vivo, underscoring its therapeutic potential [56].

In clinical samples of OSCC and mouse xenograft models, the infiltration of nociceptive nerve endings is significantly associated with poor prognosis and enhanced tumor progression in patients. A 2024 study demonstrated that the excessive accumulation of adenosine driven by CD73 up-regulation in the TME is closely linked to activation of the adenosine A_2A_ receptor (A2AR) on trigeminal ganglion neurons. Further experiments revealed that overstimulation of adenosine A2AR receptor in trigeminal nerves markedly induced the release of CGRP, which could be effectively suppressed by the selective A2AR receptor inhibitor SCH58261. CGRP directly acts on CGRP receptors and promotes tumor growth by activating the ERK and YAP signaling pathways, while the CGRP receptor antagonist rimegepant and A2AR receptor antagonist istradefylline (clinically available) can block this tumor-promoting effect [203]. In the low-glucose TME of OSCC, cancer cells can co-opt nociceptive nerves to adapt to nutrient-poor conditions. Specifically, reactive oxygen species (ROS)-driven c-Jun activation induces tumor NGF secretion, which programs nociceptors to release CGRP. Neuron-derived CGRP in turn promotes cytoprotective autophagy in cancer cells via Rap1-mediated disruption of the mTOR–Raptor complex. Notably, glycolysis- or angiogenesis-targeted nutrient-starvation therapies exacerbate this neuro–tumor feedback loop, whereas blockade of CGRP signaling enhances therapeutic efficacy [204].

In p53 mutant OSCC, tumor-associated trigeminal sensory nerves undergo functional reprogramming and adrenergic transdifferentiation, accompanied by enhanced neuritogenesis, in response to cancer-derived EV-delivered miRNAs (characterized by increased miR-21 and miR-324 and loss of miR-34a). This reprogramming results in the formation of newly generated adrenergic nerve fibers from sensory neurons. These transdifferentiated nerves, in turn, promote tumor growth via adrenergic signaling. Consistently, tumor progression is inhibited by sensory denervation or pharmacological blockade of adrenergic receptors, but not by chemical sympathectomy of preexisting adrenergic nerves, demonstrating that sensory-to-adrenergic phenotypic reprogramming or transdifferentiation is a critical driver of tumor progression [104]. Besides transferring miRNAs, cancerous EVs from head and neck squamous cell carcinoma have also been shown to deliver axon guidance proteins like EphrinB1 to sensory nerves, promoting sensory innervation and neuritogenesis, which facilitates tumorigenesis [105]. These important findings deepen our understanding of the specific modes of sensory nerve–cancer crosstalk through EVs and highlights potential therapeutic targets for anticancer intervention.

##### Indirect interactions

In melanoma, tissue-resident nociceptor neurons develop pain hypersensitivity, undergo transcriptomic reprogramming, and exhibit enhanced neurite outgrowth in response to melanoma-derived secretory leukocyte protease inhibitor (SLPI). This dense nociceptor innervation exerts immunomodulatory effects in the TME by releasing high levels of immunomodulatory neuropeptides, such as CGRP, which induce the exhaustion of CD8^+^ T cell exhaustion—characterized by up-regulation of immune checkpoint receptors including PD-1, LAG3, and TIM3—through activation of RAMP1. Consequently, nociceptor-derived CGRP weakens antitumor immune responses and promotes an immunosuppressive environment. Importantly, the antitumor immunity can be restored by genetic ablation or pharmacological silencing of nociceptor neurons, as well as blockade of the CGRP–RAMP1 axis [205]. Consistently, another melanoma study demonstrated that afferent sensory nerves contribute to tumor progression by inhibiting effective antitumor immune responses within the TME. This neural-mediated immunosuppression is associated with impaired high endothelial venule maturation and defective tertiary lymphoid structure formation. Sensory denervation enhances leukocyte recruitment, reduces the accumulation of immunosuppressive lymphoid and myeloid populations, and promotes effector T cell activation within the TME [206].

Similarly, in OSCC, sensory nerves release the CGRP to promote tumor growth and suppress intratumoral lymphocyte infiltration, whereas in CGRP-deficient mice, tumors grow markedly slower and show increased CD4^+^, CD8^+^ T cell, and natural killer (NK) cell infiltration. CD4^+^ T cells within the TME express substantially higher levels of Ramp1 than those in lymph nodes or normal tongue tissue, indicating that the CGRP–RAMP1 neuro-immune axis enables tumors to evade immune surveillance [207].

Another study employs both melanoma and HNSCC to demonstrate that tumors actively recruit and reprogram TRPV1^+^ nociceptor neurons to establish an immunosuppressive microenvironment through EVs. Genetic ablation of these neurons, or tumor cells engineered to lack small EV (sEV) release, markedly impairs tumor growth. Patient-derived sEVs enhance capsaicin sensitivity in DRG neurons, indicating heightened nociceptor reactivity upon tumor exposure. Once infiltrated into tumors, nociceptors undergo profound transcriptional reprogramming characterized by injury-associated gene signatures and increased production of neuromodulators. SP released from nociceptors stimulates IL-6 secretion by tumor cells, while tumor-derived IL-6 and sEV cargo further amplify neuronal activation, forming a feed-forward loop. These neuronal and tumor-derived factors cooperatively drive the expansion, recruitment, and suppressive function of MDSCs and promote exhaustion programs in CD8^+^ T cells, positioning nociceptor neurons as key orchestrators of tumor-induced immunosuppression [208].

In the TME of PDAC, nociceptor nerves interact with CAFs through CGRP and NGF to inhibit the expression of IL-15 of CAFs, which suppresses the infiltration and cytotoxic functions of NK cells, thereby promoting the progression of PC and contributing to cancer-related pain. Antagonizing RAMP1 to counteract the effects of CGRP may represent a therapeutic strategy that not only inhibits the progression of PC but also alleviates patient pain [209].

## Environmental and Patient Pathophysiological Factors Shaping Nerve–Cancer Interactions

Environmental and patient pathophysiological factors, including psychological states, environmental stressors, pain, microbiota, aging, and obesity, as well as lifestyle behaviors (such as smoking, diet, exercise, and circadian disruption), profoundly influence cancer risk [14].

### Psychological states

Psychological stress is an inevitable aspect of modern life. Research indicates that adversity, social isolation, despair, and various stressors are closely associated with poor prognosis, increased symptoms burden, early metastasis, and reduced life expectancy in cancer patients. Psychological stress classically activates 2 major systemic stress pathways: the SAM and HPA axis, resulting in the systemic secretion of epinephrine, norepinephrine, and glucocorticoids, which are well-established promoters of cancer progression [5,15,67,210]. Importantly, both axes primarily exert their effects through circulating hormones rather than direct neural innervation of the TME. The SAM axis involves preganglionic sympathetic fibers releasing ACh onto chromaffin cells in the adrenal medulla, whereas the HPA axis is a purely endocrine cascade without peripheral neural projections to the TME [210]. Therefore, although these systemic mechanisms are biologically important, HPA-mediated endocrine signaling falls outside the primary scope of this review, which focuses on direct peripheral neural mechanisms.

Beyond these classical endocrine pathways, accumulating evidence indicates that psychological stress can remodel the TME and peripheral immune organs through direct peripheral neural circuits, particularly via sympathetic innervation [5,15]. Rather than acting solely through circulating hormones, these nerve–tumor and nerve–immune interactions reshape immune composition, stromal dynamics, and local neurotransmitter availability, ultimately altering the TME. Such peripheral neural mechanisms constitute the central focus of this section.

Chronic neuropsychological stress, induced through restraint, chronic unpredictable stress, or nociception, activates central stress circuits that drive strong output from the SNS, leading to the increase of norepinephrine release within the pancreatic TME, rather than relying on adrenal hormones [68]. Local adrenergic signaling suppresses the RNA demethylase ALKBH5 in PDAC cells through an β2-AR–CREB–CHD4 epigenetic mechanism, resulting in widespread m6A hypermethylation of tumor RNAs. These m6A-modified transcripts are selectively packaged into EVs and transferred to sensory and sympathetic neurons, where they function as miRNA sponges that enhance axonogenesis-related gene expression. The resulting tumor hyperinnervation increases local norepinephrine and neuropeptide release, amplifies ERK signaling, accelerates tumor growth, and promotes PNI. This work establishes psychological stress as a driver of PDAC progression through a defined sympathetic neural circuit and identifies ALKBH5-dependent EV–neuron communication as a central mechanism linking stress to tumor innervation and malignancy [68].

Chronic psychosocial stress also promotes tumor dissemination through sympathetic remodeling of the lymphatic niche. Repeated inescapable restraint activates central stress pathways that sustain sympathetic output to tumor-associated lymphatic vessels and draining lymph nodes [69]. Local norepinephrine release engages β-adrenergic receptors (β-ARs) on tumor cells, lymphatic endothelial cells, and TAMs. Through this peripheral neural circuit, stress signaling increases intratumoral lymphatic vessel density, dilates collecting lymphatic vessels, and accelerates lymph flow. These structural and functional alterations enhance vascular endothelial growth factor C (VEGFC)-dependent lymphangiogenesis and cyclooxygenase 2 (COX2)–PGE2-mediated inflammatory signaling, creating efficient conduits for tumor cell escape and markedly increasing lymphogenous metastasis. Pharmacologic β-AR-blockade interrupts this sympathetic–lymphatic axis and prevents stress-induced lymphatic remodeling, highlighting peripheral SNS signaling as a modifiable driver of metastatic spread [69].

In addition to sympathetic pathways, recent work has revealed long-range polysynaptic circuits linking central stress processing to enteric neural regulation of tumors. Li et al. [70] identified a brain–gut circuit in CRC in which stress-responsive GABAergic neurons in the LS project polysynaptically through the LH and the SPN to enteric cholinergic neurons, forming an LS–LH–SPN–ENS axis. These enteric neurons extend fibers into the TME and release GABA, which activates ε-subunit-containing GABA_A receptors on CRC cells, promoting sustained tumor growth. Chronic restraint stress potentiates neural activity along this septo-enteric circuit, amplifying neurotransmitter release and exacerbating tumor progression [70]. This study provides a mechanistic example of how central emotional processing can be translated into peripheral neural signals that directly modulate tumor biology.

Anxiety, a form of emotional stress, has been shown to accelerate cancer progression, whereas social interaction alleviates anxiety and inhibit tumor growth [71]. Social interaction activates a cortico-amygdala excitatory pathway in which glutamatergic neurons in the anterior cingulate cortex (ACC) stimulate glutamatergic neurons in the basolateral amygdala (BLA). This recruits GABAergic neurons in the central amygdala lateral subdivision (CeL), which inhibit corticotropin-releasing hormone (CRH)-expressing neurons in the central medial amygdala (CeM), the principal output hub driving sympathetic outflow. Suppression of CeM output reduces sympathetic nerve activity and norepinephrine release within the TME, thereby enhancing the antitumor immune response and slowing the progression of breast cancer [71].

Beyond the attenuation of sympathetic tone through social interaction, other positive psychological states exert similar tumor-suppressive effects via defined neural–immune pathways. Ben-Shaanan et al. [72] demonstrated that activation of dopaminergic neurons in the VTA, a central node of the brain’s reward circuitry, suppresses sympathetic output to the bone marrow. Reduced noradrenergic signaling diminishes TH^+^ sympathetic innervation and reprograms MDSCs toward a less immunosuppressive phenotype. Upon infiltration into tumors, these reprogrammed MDSCs permit stronger CD8^+^ T cell activation and cytotoxicity, ultimately restraining tumor growth. Thus, positive emotional states can modulate cancer progression through a VTA–SNS–bone marrow neural–immune axis, reinforcing the concept that psychological states influence tumor biology via peripheral neural circuits [72].

### Environmental temperature

Environmental temperature is a fundamental physiological variable that strongly influences peripheral neural activity and immune homeostasis. Deviations from thermoneutrality engage sympathetic neural circuits, reshape metabolic and immune programs, and thereby modulate tumor–immune interactions within the TME.

Mice housed at standard laboratory temperatures (22 to 23 °C) experience chronic cold stress that continuously activates the SNS and drives norepinephrine release from sympathetic fibers innervating the TME. This persistent β-AR signaling disrupts the metabolic programming required for effective T cell activation, suppressing T cell receptor (TCR) signaling, reducing GLUT-1 expression, and impairing both glycolysis and oxidative phosphorylation. As a result, CD8^+^ T cells accumulate features of exhaustion, including elevated inhibitory receptors and diminished cytokine production, while progenitor exhausted subsets decline. These changes weaken antitumor immunity and limit responsiveness to checkpoint blockade. Blocking β-AR signaling reverses these effects by restoring T cell metabolism, enhancing effector cytokine production, increasing CD28 expression, and expanding progenitor exhausted T cells, thereby strengthening antitumor immune responses and providing a rationale for combining β-blockers with immunotherapy [73].

### Pain

Pain is one of the most common complications associated with tumors. In cancer patients undergoing treatment, more than one-third experience severe pain [211]. Research has shown that pain can suppress immune responses and promote tumor growth. However, the relationship among pain, tumors, and immunity is highly complex [212]. As systematically reviewed in the section *Neurogenesis and tumor innervation of sensory nerves*, sensory nerve innervation—particularly nociceptive neurons and their neuropeptides—engages in direct interactions with tumor cells and the TME. A 2022 study led by S. Talbot demonstrated that melanoma cells actively recruit and sensitize TRPV1^+^ nociceptor neurons by secreting factors such as SLPI, thereby inducing axonal sprouting and enhanced neuropeptide release including CGRP. This sensitized neuronal state produces pronounced pain behaviors in the host, such as thermal hypersensitivity, and the intensity of pain correlates with the degree of CD8^+^ T cell exhaustion within the TME. CGRP released from activated nociceptors acts through the CALCRL/RAMP1 receptor on CD8^+^ T cells, directly suppressing their effector function and promoting their transition into a PD-1^+^LAG3^+^TIM3^+^ exhausted phenotype, thereby weakening antitumor immunity. Importantly, blocking nociceptor signaling markedly reduces both pain hypersensitivity and tumor growth, indicating that the pain pathway itself is functionally coupled to tumor progression [205].

In another study on PDAC, CAFs in PDAC release NGF, which stimulates TRPV1^+^ nociceptor neurons to produce and secrete CGRP, driving both neuronal sensitization and the development of visceral cancer pain. Activated nociceptors, in turn, signal back to CAFs through CGRP–RAMP1, suppressing IL-15 production and thereby reducing the infiltration and cytotoxicity of NK cells, creating an immune-suppressed microenvironment that accelerates tumor progression. This bidirectional loop not only amplifies nociceptive signaling—manifesting as mechanical allodynia, spontaneous pain, and impaired mobility measured through von Frey, conditioned place aversion, and open-field tests—but also tightly couples pain severity with tumor aggressiveness. Disrupting this nociceptor pathway, either by ablating TRPV1^+^ neurons or blocking CGRP signaling, simultaneously reduces tumor burden and alleviates cancer-induced pain, underscoring pain as an integral mechanistic component of tumor–nerve–immune crosstalk in PDAC [209].

Collectively, these findings reframe tumor-induced pain not merely as a symptom but as an active mechanism through which tumors exploit the neuro-immune axis to evade immune surveillance.

Pain and psychological stress are closely intertwined experiences in cancer patients and frequently co-occur with circadian rhythm disruption, such as sleep fragmentation and insomnia [213,214], whereas stress can lower pain thresholds through central sensitization and autonomic activation [215]. Thus, cancer-associated pain comprises both a sensory–nociceptive component and an affective–emotional dimension, often accompanied by circadian rhythm disturbances, each capable of engaging peripheral neural and immune pathways within the TME.

### Microbiota

The microbiota markedly influences the pathogenesis and treatment outcomes of human malignant tumors [216], with polymorphic microbiomes recognized as a hallmark feature of cancer [217]. Increasing evidence conceptualizes tumor regulation within a complex neuro–microbiota–immune network, in which gut microbiota and their metabolites can directly modulate ENS activity and thereby acting on the immune microenvironment or tumor cells. Additionally, the ENS, in turn, shapes microbial composition and function through neural signaling. Subsequently, microbiota-derived metabolites may influence cancer progression either directly or indirectly via mucosal and systemic immune modulation [200].

A representative example of this microbiota-centered regulation is the IVA–5-HT–CRC stemness axis. Studies have shown that specific bacterial communities are enriched in CRC patients and mouse models, characterized by elevated production of short-chain fatty acid-related metabolites. Fecal microbiota transplantation (FMT) experiments further demonstrated that CRC-associated microbiota are sufficient to induce Tph2 up-regulation in healthy recipient mice, indicating a direct capacity of the microbiota to regulate enteric neuronal metabolic programs. Among CRC-associated metabolites, IVA was identified as the key factor capable of markedly inducing Tph2 expression. IVA acts directly on myenteric plexus neurons, relieving NuRD complex-mediated repression of the Tph2 promoter, thereby enhancing enteric serotonin (5-HT) synthesis, which subsequently promotes CRC stem cell self-renewal and tumor progression [187].

### Aging and obesity: Potential impacts

Aging is a major risk factor for cancer and is associated with the accumulation of chronic genetic damage, epigenetic remodeling, alterations in the tissue microenvironment, and progressive dysfunction of both innate and adaptive immunity [218]. Although direct evidence linking aging-driven PNS alterations to cancer progression remains limited, substantial data demonstrate that aging profoundly remodels peripheral neural structure and function, with many features overlapping known nerve–tumor interaction phenotypes.

Clinical evidence supports an association between aging and autonomic dysfunction in cancer. A recent study in patients with gastrointestinal tumors demonstrates that advanced age is associated with severe autonomic impairment, particularly sympathetic–parasympathetic imbalance. Multivariate analyses further linked autonomic dysfunction to aggressive tumor features, including increased tumor size and vascular invasion [219].

At the structural level, aging leads to a reduction in peripheral nerve fibers and glial density, accompanied by functional abnormalities [220], implying changes that may alter neural innervation and neurotransmitter release within the TME. At the cellular level, aging induces marked alterations in neurons and glial cells. Human postmortem studies demonstrate an age-dependent accumulation of senescent neurons in DRG [221]. In aged rodents, DRG and peripheral nerves exhibit shifts in Schwann cell composition, activation of neurodegeneration-associated pathways, and altered metabolic programs. Notably, aging and injury drive neuronal senescence characterized by pro-inflammatory signaling (e.g., IL-6), increased excitability, and nociceptor-like phenotypes, closely resembling cancer-associated nociceptive sensory nerve activation [221,222]. Schwann cells exhibit partial dedifferentiation and spontaneous activation of repair-associated programs [223], phenotypes reminiscent of pro-tumorigenic nerve remodeling observed in PNI. Aging of the PNS is further marked by chronic inflammation (inflammaging). Even in the absence of injury, aged nerves show increased macrophage infiltration and elevated inflammatory chemokines such as CCL2 and CCL11. Following injury, aged nerves display delayed immune activation followed by sustained inflammation and impaired repair capacity [223].

These neural alterations may synergize with immunosenescence, which reshapes the TME by favoring immunosuppressive cell recruitment and therapy resistance. The pro-inflammatory phenotype of aged peripheral nerves may cooperate with systemic immune aging to create a tumor-permissive niche [224,225]. Finally, aging compromises peripheral nerve maintenance and regeneration, potentially altering tumor-induced axonogenesis and the dynamic remodeling of tumor innervation [223].

Obesity may promote tumor development by reshaping both the PNS and immune homeostasis. Extensive evidence supports that obesity induces sympathetic overdrive accompanied by reduced parasympathetic activity [226–228], which can be partially reversed by weight loss [226,229]. Obesity also modifies the expression and sensitivity of peripheral neurotransmitter receptors, particularly adipocyte adrenergic receptors [230–232]. Such autonomic imbalance and altered adrenergic signaling are likely to create a tumor-promoting neuroenvironment.

Beyond functional dysregulation, obesity severely impairs peripheral nerve integrity. Long-chain fatty acids and inflammatory mediators can disrupt the blood–nerve barrier and trigger neurogenic inflammation. DRG neurons, which are partially exposed to systemic metabolic stress, release vasoactive and chemotactic factors that enhance vascular permeability and immune cell recruitment. While initially protective, chronic obesity-associated inflammation leads to structural nerve damage and neuropathic changes [233]. Animal models of high-fat diet-induced obesity demonstrate increased macrophage accumulation, elevated TNF-α and IL-1β expression, and peripheral neuropathy even in the absence of overt diabetes [234,235].

Obesity further remodels the tumor immune microenvironment through systemic and local inflammation. Obesity-associated adipose tissue inflammation resembles chronically injured tissue, characterized by immune cell infiltration and stromal remodeling, and correlates with poor prognosis in cancers such as breast and tongue carcinoma [236]. Together, obesity-driven autonomic imbalance, peripheral nerve dysfunction, and chronic inflammation may synergistically establish a tumor-permissive niche.

### Lifestyle behaviors: Potential impacts

Although most studies linking lifestyle behaviors to tumor progression do not explicitly address nerve–cancer crosstalk, accumulating evidence suggests that several common behaviors can indirectly modulate tumor biology by engaging peripheral neural signaling pathways. Notably, certain lifestyle factors can directly introduce neuroactive compounds (e.g., nicotine from smoking), alter systemic or local neurotransmitter levels (e.g., GABA elevation following sleep deprivation), or sensitize tumor cells to neural inputs by up-regulating neurotransmitter receptors. Through these mechanisms, lifestyle-associated signals may synergize with, amplify, or potentially antagonize endogenous neural signaling of physiological or pathological origin. Such interactions, although largely unexplored, may substantially influence the innervated niche and warrant further investigation.

#### Smoking

Environmental carcinogens, such as tobacco smoke, are important mutagens and inflammatory inducers [237]. As the most representative addictive component of tobacco smoke, nicotine exerts broad tumor-promoting effects across multiple organs, including tumor initiation, proliferation, invasion, and metastasis, largely through engagement of neurotransmitter receptors rather than direct neural inputs. In lung squamous cell carcinoma, nicotine enhances the transcription and expression of GATA4 and GATA6 by facilitating the binding of Sp1 to the α7 nicotinic acetylcholine receptor (nAChR) promoter, thereby promoting tumorigenesis and progression [238]. In bladder cancer, nicotine activates both nAChR and β-ARs, triggering ERK1/2–STAT3 signaling, NF-κB activation, cyclin D1 up-regulation, and enhanced proliferation [239]. Similarly, in gastric cancer, nicotine induces epithelial–mesenchymal transition (EMT) and markedly enhances the migratory capacity of cancer cells by down-regulating E-cadherin and up-regulates ZEB1 and Snail through α7 nAChR [240].

In PC, nicotine and cigarette smoke activate the Janus kinase 2 (JAK2)/STAT3 signaling pathway through the α7 nAChR, leading to MUC4 up-regulation and downstream activation of HER2, c-Src, and FAK, thereby promoting the migration of cancer cells [241]. In CRC, nicotine stimulates β2-AR to enhance COX2, PGE2, and VEGF expression, supporting tumor growth and angiogenesis [242]. At the metabolic level, nicotine activates nAChRs to induce hypoxia-inducible factor 1α (HIF1α)-dependent glycolytic reprogramming, further accelerating the migration of cancer cells and tumor progression [243].

Collectively, although these studies do not directly interrogate nerve–tumor interactions, they highlight nicotine as a systemic neuroactive agent capable of hijacking canonical neurotransmitter receptors on tumor cells, thereby potentially interacting with endogenous cholinergic and adrenergic signaling within innervated niches.

#### Diet

Dietary factors can also modulate peripheral neural signaling and sensitize tumor cells to neural cues. Chronic alcohol consumption up-regulates the expression and sensitivity of α3, α5, and α7 nAChRs on PDAC cells and ductal epithelial cells, which results in increased production of norepinephrine and epinephrine. These catecholamines subsequently activates β-adrenergic signaling in an autocrine/paracrine manner, elevating cAMP and PKA activity, and driving phosphorylation of ERK, CREB, Akt, and Src, thereby promoting PDAC proliferation and migration. Treatment with GABA effectively mitigates these effects, suggesting that dietary or metabolic modulation of neurotransmitter balance may counteract alcohol-associated tumor promotion [244].

In parallel, a high-fat diet enhances tumor sensitivity to adrenergic signaling in CRC. Elevated palmitic acid directly up-regulates β2-AR expression in an Sp1-dependent manner, amplifying cellular responses to catecholamines. β2-AR activation engages the cAMP–PKA pathway and selectively phosphorylates hormone-sensitive lipase, driving lipolysis, fatty acid oxidation, mitochondrial respiration, and ATP production, thereby promoting tumor growth [245].

These findings suggest that diet-induced metabolic cues can reprogram tumor cells to become more responsive to neural-derived catecholamines.

#### Exercise

Accumulating evidence indicates that exercise influences tumor progression through modulation of the PNS. Acute exercise transiently elevates circulating epinephrine and norepinephrine via sympathetic activation, which directly engage β-ARs on cancer cells. In breast cancer models, catecholamine signaling activates the Hippo tumor-suppressor pathway, leading to YAP phosphorylation, cytoplasmic sequestration, suppression of YAP/TAZ target genes, and reduced tumor cell viability [246].

In tumor-bearing mice, voluntary exercise markedly suppresses tumor growth and metastasis across multiple models. This effect is mediated by exercise-induced activation of the sympathetic–adrenal axis, resulting in epinephrine-driven mobilization of cytotoxic NK cells and muscle-derived IL-6-dependent recruitment of these cells into tumors. Blockade of β-adrenergic signaling abolishes both NK cell infiltration and the antitumor effects of exercise [247]. Additional studies demonstrate that exercise broadly remodels the tumor immune microenvironment by promoting M1 macrophage polarization, reducing neutrophil accumulation, increasing NK cell density, enhancing CD8^+^ T cell infiltration, and suppressing FoxP3^+^ regulatory T cells, collectively fostering an antitumor milieu [248].

In patients with cancer, structured exercise programs improve autonomic balance by reducing sympathetic overactivity and enhancing vagal tone, which may secondarily modulate inflammation and antitumor immunity [249]. Exercise also mitigates chemotherapy-induced peripheral neuropathy (CIPN), highlighting its capacity to preserve peripheral neural integrity and indirectly stabilize the tumor-innervated niche [250–252].

Beyond its direct physiological effects, exercise also exerts beneficial influences on psychological states, including reductions in anxiety, depressive symptoms, and perceived stress, alongside improvements in sleep quality and emotional well-being [253,254]. These psychological benefits may indirectly contribute to tumor control by dampening chronic stress-associated sympathetic activation and supporting antitumor immunity, although direct causal links remain incompletely defined.

Notably, the effects of exercise are context-dependent. Excessive or involuntary exercise may paradoxically promote tumor progression, possibly not due to physical activity per se but as a consequence of stress-induced neuroendocrine activation. Intense exercise-related stress elevates epinephrine and prolactin levels, enhancing mammary carcinogenesis without a compensatory increase in NK cell-mediated immunity [255].

#### Circadian disruption

The circadian rhythm regulates a variety of physiological processes, including the sleep–wake cycle, metabolism, and cell proliferation. Circadian genes exert broad control over cancer-relevant pathways such as cell-cycle regulation, DNA repair, apoptosis, and stemness [218]. Disorders of the circadian rhythm is associated with elevated risk of cancer [256].

Sleep deprivation represents a prominent circadian disturbance that can modulate tumor progression through neuroactive metabolites and intercellular communication. Sleep deprivation has been shown to enhance the proliferation and migration of colon cancer cells by elevating systemic GABA levels. In tumor cells, GABA induces miR-223-3p expression, inhibits the E3 ligase CBLB, reduces c-MYC ubiquitination, and directly promotes tumor growth. In parallel, tumor-derived exosomes enriched in miR-223-3p are taken up by macrophages, activating MAPK signaling and driving M2 polarization. These M2 macrophages secrete IL-17, which further enhances tumor proliferation and migration [257]. Together, these findings illustrate how circadian disruption can rewire nerve–immune–tumor communication through neurotransmitter-linked pathways, despite the absence of direct neural innervation changes.

## Application of Nerve–Cancer Interaction in Tumor Therapy

### Denervation

**Fig. 4.**
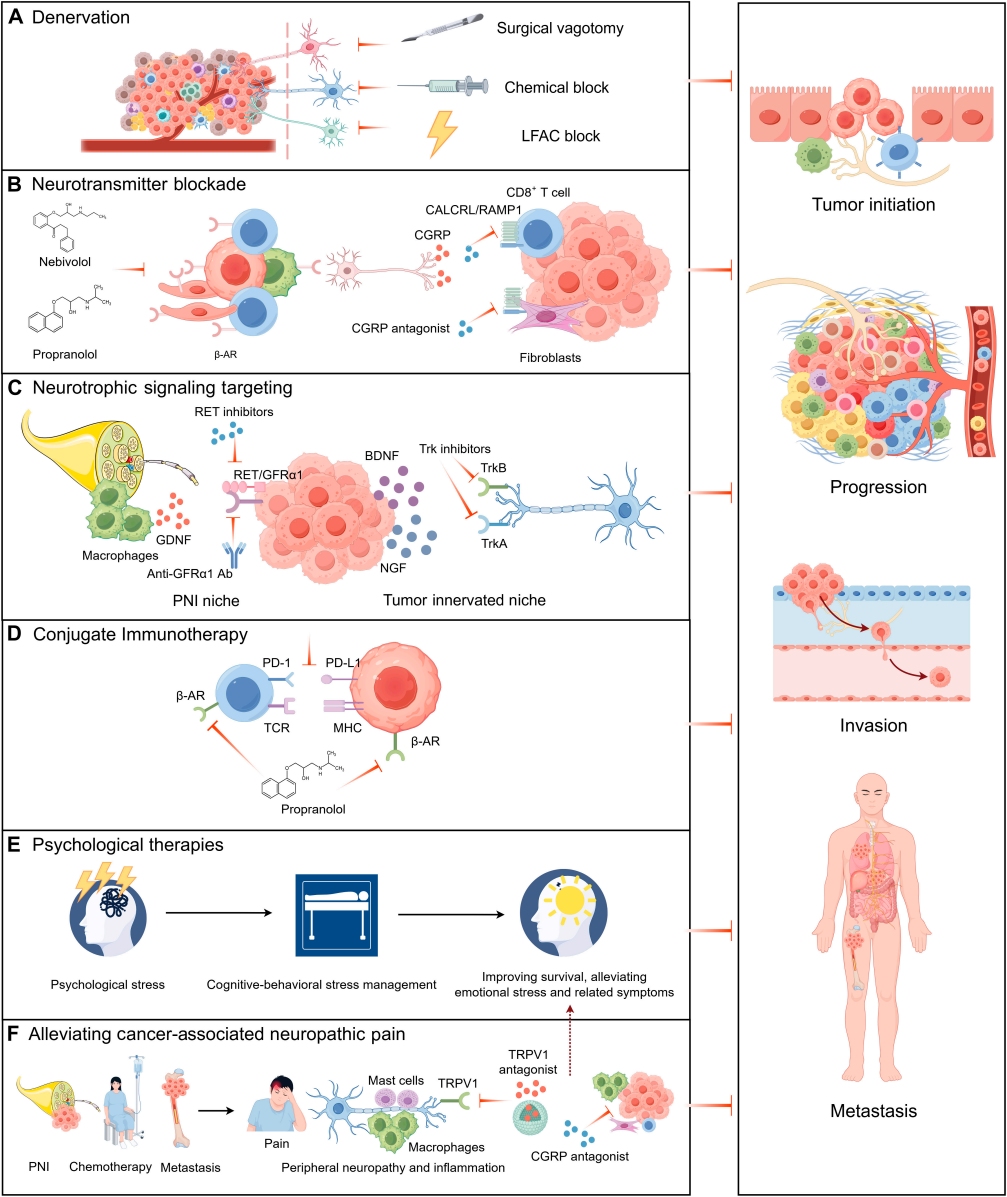
Clinical translation of the nerve–cancer interactions in oncology. This schematic illustrates multifaceted therapeutic strategies targeting neuro-cancer crosstalk. (A) Denervation strategies, including surgical vagotomy, chemical blockade, and bioelectronic interventions such as low-frequency alternating current (LFAC) block. (B and C) Pharmacological targeting of nerve-derived paracrine signaling, comprising the inhibition of neurotransmitters—most notably via nonselective or selective β-blockers and emerging CGRP antagonists—as well as the blockade of neurotrophic pathways, specifically targeting the neurotrophic receptor tyrosine kinase (NTRK) and RET/GFRα1 axes. (D) Combinatorial immunotherapy, highlighting the synergistic potential of combining β-blockers with immune checkpoint inhibitors (ICIs), a strategy currently validated in clinical trials. (E) Psychosocial interventions, such as cognitive–behavioral stress management (CBSM), which have been shown to improve survival in cancer patients and alleviating anxiety, depression, and stress-induced symptoms. (F) Mitigation of cancer-associated neuropathic pain. Emerging evidence suggests that cancer pain is mechanistically coupled to tumor progression rather than being a mere by-product of tissue damage; thus, pain management offers dual benefits—slowing tumor development while enhancing the patient’s emotional well-being and overall quality of life. CALCRL, calcitonin receptor-like receptor; RAMP1, receptor activity modifying protein 1; GDNF, glial cell-derived neurotrophic factor; RET, Ret proto-oncogene; GFRα1, GDNF family receptor α1; Trk, tropomyosin receptor kinase; β-AR, β-adrenergic receptor; PD-1, programmed cell death protein 1; PD-L1, programmed death-ligand 1; TCR, T-cell receptor; MHC, major histocompatibility complex; TRPV1, transient receptor potential vanilloid 1.

As we reviewed in the sections *Neurogenesis and tumor innervation—Neural regulation of the TME* and *PNS–tumor interactions within the TME*, poorer clinical outcomes were closely associated with tumor innervation [48,258]. The classic strategy for disrupting this nerve–cancer interactions involves surgical methods or chemical agents, in both clinical and preclinical studies, to denervate the nerves and subsequently reduce the density of TME nerves [259–261] (Fig. 4A). In tumors located in the stomach, pancreas, prostate, and colon, denervation of the vagus, sympathetic, or sensory nerves leads to a considerable decrease in tumor burden, angiogenesis, cancer stemness, and pain, ultimately resulting in an extended survival period [262]. Vagotomy is a surgical procedure aimed at removing vein network inputs from various organs, including the stomach, liver, pancreas, small intestine, large intestine, and rectum. This procedure involves cutting 1 (unilateral) or 2 (bilateral) vein network trunks above the gastroesophageal junction while preserving inputs to vital organs such as the heart and lungs [263]. It has been discovered that surgical or chemical denervation of the stomach, achieved through vagotomy or local Botox injection, markedly reduces the incidence and progression of PCa [48,258]. According to Zhao et al. [151], carcinogenesis was effectively suppressed through surgical denervation via bilateral or unilateral truncal vagotomy, or by administering an injection of botulinum toxin, which inhibits the release of ACh from axon terminals. For unresectable PC, chemical vagal nerve transection is a safe and straightforward procedure. It can markedly alleviate or prevent tumor-related abdominal pain and may provide survival benefits for those who experience pain prior to the surgery [264]. More importantly, cancer cachexia can be alleviated by multiple forms of vagal blockade—including surgical right vagotomy, chemical block, implanted electrical LFAC (low-frequency alternating current) block, and noninvasive transcutaneous LFAC block—which all reduced vagal hyperactivity and restored liver metabolic function, improving weight, muscle mass, behavior, and survival in mice [265] (Fig. 4A). Across these approaches, disrupting vagal output consistently attenuated cachexia without affecting tumor size, highlighting the vagus nerve as a therapeutic target.

Notably, both sympathetic and parasympathetic denervation substantially reduce the amount of aberrant crypt foci (precancerous lesion) in rats during the early stage of 1,2-dimethylhydrazine (DMH)-induced colon carcinogenesis. However, in the later stage, only parasympathetic denervation led to marked reductions in tumor incidence, tumor volume and weight, proliferative activity, and angiogenesis, whereas sympathetic denervation does not produce comparable antitumor effects. This divergence highlights that different branches of the ANS exert fundamentally distinct influences on CRC progression [266].

In addition, the therapeutic efficacy of denervation is highly dependent on strategy and technical execution. A 2023 study introduced a microsurgical approach that achieves radical and persistent tumor denervation by completely severing all neural connections while restoring blood flow via microvascular anastomosis, thereby creating a critical therapeutic window in which the tumor remains perfused but cannot be reinnervated. Within this window, primary tumors consistently regressed and ultimately disappeared, resulting in high long-term survival; notably, in animals bearing multiple tumors, untreated distant lesions also regressed, indicating a systemic antitumor effect. In contrast, partial denervation, preservation of neurovascular bundles, or stepwise tissue separation failed to reproduce these outcomes, underscoring the necessity of complete and sustained denervation. If validated in future clinical trials, this strategy could represent a chemotherapy- and radiotherapy-independent therapeutic option, particularly for patients who cannot tolerate conventional treatments or those with advanced or multifocal disease [267].

However, some studies report different effect of tumor denervation. In melanoma, sensory nerve fibers can extend deep into cancerous tissue and align closely with intratumoral blood vessels. In this context, both genetic and chemical ablation of sensory nerves lead to a marked acceleration of tumor growth, suggesting that the impact of denervation is highly dependent on tumor type and on the specific sensory neuron subtypes involved [268].

### Neurotransmitter blockade

Targeting neurotransmitter signaling represents the most direct and pharmacologically accessible strategy to disrupt nerve–cancer crosstalk. Most current therapeutic explorations focus on repurposing neurotransmitter or neuropeptide receptor antagonists, inhibitors, or blockers to inhibit tumor growth and progression.

Several randomized clinical trials, prospective cohort study, and retrospective analyses have identified an association between the use of neurotransmitter blockers and improved tumor outcomes, highlighting the potential of existing neuromodulators as effective anticancer treatments (Table 2). Among these, β-AR antagonists are the most extensively studied and clinically investigated class (Fig. 4B, also see Supplementary File 2 for detailed review and discussion).

**Table 2.** Clinical application of neurotransmitter blockers

Cancer type	Neurotransmitter blockers	Type of neurotransmitter blockers	Preclinical/clinical trial phase	Experimental group	Control group	Sample size	Effective or Noneffective	Effect	Reference (PMID/Source)
NSCLC	β-Blockers	Not mentioned	Cohort study	Received β-Blockers	Not received β-Blockers	6,270	Noneffective	The use of β-Blockers did not reduce the risk of mortality.	25204805
NSCLC	β-Blockers	Not mentioned	Retrospective observational study	β-Blockers + chemotherapy	Chemotherapy	107	Effective	The use of β-Blockers during chemotherapy was associated with improved OS.	24289634
Breast cancer	β-Blockers	Propranolol (*n* = 70), atenolol (*n* = 525)	Retrospective observational study	Received β-Blockers (propranolol or atenolol)	Not received β-Blockers	5,333	Effective	The use of propranolol probability decreased breast cancer-specific mortality. The use of atenolol was not associated with breast cancer-specific mortality.	21632503
Triple-negative breast cancer	β-Blockers	Not mentioned	Retrospective observational stud	Received β-Blockers	Not received β-Blockers	800	Effective	The use of β-Blockers was associated with a decreased risk of breast cancer-related recurrence, metastasis, and death.	23912960
Melanoma	β-Blockers	Bisoprolol, atenolol, nebivolol, propranolol, sotalol, carvedilol, timolol, betaxolol, labetolol, nadolol, and metoprolol	Retrospective observational study	Received β-Blockers	Not received β-Blockers	741	Effective	The use of β-Blockers enhanced survival and reduced melanoma mortality.	24182700
Thick (Breslow thickness >1 mm) malignant melanoma	β-Blockers	Not mentioned	Retrospective observational study	Received β-Blockers	Not received β-Blockers	121	Effective	The exposure to β-Blockers for 1 year or more was associated with a reduced risk of progression of thick malignant melanoma.	21518948
Epithelial ovarian cancer	β-Blockers	Not mentioned	Cohort study	Received β-Blockers	Not received β-Blockers	248	Effective	The use of β-Blockers was associated with longer disease-specific survival, longer OS, and a 54% reduction in the chance of death.	22819786
Locally advanced and metastatic melanoma	β-Blockers	Propranolol	Phase I clinical trial	β-Blockers (propranol)	Not application	9	Effective	The combination of propranolol with pembrolizumab was safe, increasing IFN-γ and decreasing interleukin-6.	33127652
Prostate cancer with high-risk or metastatic disease	β-Blockers	Not mentioned	Cohort study	Received β-Blockers	Not received β-Blockers	3,561	Effective	The use of β-Blockers reduced prostate cancer-specific mortality in high-risk or metastatic patients.	23351721
Prostate cancer	β-Blockers	β1-selective blocker, unselective β-blocker	Cohort study	Received β-Blockers	Not received β-Blockers	6,515	Effective	The use of β-Blockers was not associated with prostate cancer-specific mortality, but in the subgroup of men receiving androgen deprivation therapy, the use of β-Blockers reduced prostate cancer-specific mortality.	22821802
Resected high-risk stage III melanoma	β-Blockers	Not mentioned	Phase III trial	Received β-Blockers	Not received β-Blockers	1,019	Noneffective	The use of β-Blockers had no prognostic effect. However, β-Blockers predicted improved efficacy of adjuvant pembrolizumab treatment.	35220182
Malignant melanoma	β-Blockers	Not mentioned	Cohort study	Received β-Blockers	Not received β-Blockers	4,179	Effective	The use of β-Blockers reduced the risk of death.	21933972
Advanced prostate cancer	β-Blockers	Metoprolol	Cohort study	Androgen therapy deprivation + metoprolol	Androgen therapy deprivation	39,198	Noeffective	The use of β-Blockers was not associated with improvement in OS, prostate cancer-specific survival, and skeletal-related events.	33587939
Breast cancer	β-Blockers	Atenolol (*n* = 25), propranolol (*n* = 7), bisoprolol (*n* = 7), timolol (*n* = 4)	Retrospective observational study	Received β-Blockers	Not received β-Blockers	417	Effective	The use of β-Blockers reduced distant metastases, cancer recurrence, and cancer-specific mortality.	21317458
NSCLC	β-Blockers	Metoprolol (*n* = 89), atenolol (*n* = 43), bisoprolol (*n* = 2), propranolol (*n* = 4), sotalol (*n* = 3), nadolol (*n* = 2), carvedilol (*n* = 11), labetalol (*n* = 1)	Cohort study	β-Blockers + radiotherapy	Radiotherapy	722	Effective	The use of β-Blockers was associated with improved distant metastasis-free survival, disease-free survival, and OS.	23300016
Epithelial ovarian cancer (epithelial ovarian, primary peritoneal, or fallopian tube cancers)	β-Blockers	β1-Adrenergic receptor selective agents (*n* = 193), nonselective β antagonists (*n* = 76)	Retrospective observational study	Received β-Blockers	Not received β-Blockers	1,425	Effective	The use of β-Blockers was associated with longer OS.	26301456
NSCLC	β-Blockers	Not mentioned	Retrospective observational study	β-Blockers + immune checkpoint inhibitors	Immune checkpoint inhibitors	109	Noneffective	The use of β-Blockers was associated with improved progression-free survival.	32900613
Advanced/metastatic NSCLC	A2AR antagonist (taminadenant)	Taminadenant	Phase I study	Received taminadenant with spartalizumab	Received taminadenant	25	Not mentioned	Taminadenant with spartalizumab was well tolerated.	35254415
Advanced NSCLC	A2AR antagonist	PBF-509	Phase I/II study	NIR178 (oral A2AR antagonist)	NA	24	NA	NIR178 was well tolerated.	NCT02403193
Advanced malignancies	A2AR/A2BR antagonist	Etrumadenant	Phase I, open-label,	AB928 in combination with chemotherapy or anti-PD-1.	NA	26	NA	Favorable safety profile of AB928 combination therapy.	NCT03719326, NCT03720678, NCT03846310, NCT03629756
Refractory mCRC	A2AR/A2BR antagonist	Etrumandant	Randomized phase II clinical trial	Etrumadenant combined with zimberelimab (Z), and FOLFOX/bevacizumab (bev) regimens	Regorafenib	112	Effective	EZFB demonstrated statistically significant improvement in PFS and OS.	https://doi.org/10.1200/JCO.2024.42.16_suppl.3508
mCRC	A2AR/A2BR antagonist	AB928	Phase 1/1b, open-label study	AB928 + mFOLFOX-6	NA	21	Effective	AB928 with mFOLFOX-6 has been well tolerated.	https://doi.org/10.1158/1538-7445.AM2020-LB-387
mCRC	H1 antihistamine	Levocetirizine	Phase II open-label trial	Levocetirizine with capecitabine and bevacizumab	NA	36	Effective	Median PFS in the trial appeared to be better than other regimens used in the refractory mCRC setting.	31183190
Breast cancer	H1 antihistamine	Ctirizine, clemastine, desloratadine, ebastine, fexofenadine and loratadine	Prospective observational study	Antihistamines	Not received antihistamines	61,627	Effective	Improved survival of desloratadine users, as well as of loratadine users, relative to non-users.	32459128
Lung cancer	H1 antihistamine	Loratadine	Retrospective cohort study	Loratadine	Not received loratadine	4,522	Effective	Significant disparities were detected in the OS and PFS curves when comparing patients with and without loratadine.	38163866
Ovarian cancer	Antihistamines	Loratadine (*N* = 182), desloratadine (*n* = 123), ebastine (*n* = 30), terfenadine (*n* = 13), cyproheptadine (*n* = 5)	Prospective observational study	CAD antihistamines and non-CAD antihistamines	Not received antihistamines	5,075	Effective	Use of CAD antihistamines was associated with a reduction of mortality.	31688928
Breast cancer	H1 receptor antagonists	Desloratadine, loratadine, cetirizine, klemastine, ebastin and fenofexadine	Prospective observational study	Antihistamines	Not received antihistamines	54,406	Effective	Antihistamines have a better overall and breast cancer specific survival compared with non-users.	https://doi.org/10.1200/jco.2015.33.15_suppl.3062
Melanoma	H1 antihistamine	Desloratadin, cetirizine, loratadine, clemastine, ebastine, and fexofenadine.	Prospective observational study	Desloratadine and loratadine	Not received antihistamines	24,562	Effective	Desloratadine and loratadine use associated with improved survival.	32171023
Advanced renal cell carcinoma	H2 receptor antagonists	Cimetidine	Prospective randomized phase III trial	Natural IFN-α plus cimetidine	Natural IFN-α	71	Noneffective	Combined treatment with natural IFN-α plus cimetidine for advanced renal cell carcinoma did not result in a significant improvement in response rates or TTP compared to natural IFN-α therapy alone.	16586071
Colorectal cancer	H2 receptor antagonists	Famotidin	Double blind, placebo controlled, prospective randomized study.	Famotidine	Placebo	23	Effective	Fewer recurrences and a longer survival in the study group, the difference was not significant.	15882879
Lymph node-positive colorectal cancer	H2 receptor antagonists	Cimetidine	Retrospective cohort study	Cimetidine	Not received Cimetidine	38	Effective	Prolonged duration of cimetidine may be superior to shorter courses in delaying recurrence of colorectal cancer and improving survival.	29630589
Breast cancer	β-Blockers	Not mentioned	Prospective observational study	β-Blockers	Not received β-Blockers	14,976	Effective	β-Blocker use (vs non-use) was associated with a marginal and nonstatistically significant increase in the risk of breast cancer-specific death.	35286523
HER2-positive advanced breast cancer	β-Blockers	Nonselectiveβ-blockers (*n* = 51), selectiveβ-blockers (*n* = 43)	Retrospective hospital-based study.	Trastuzumab and any dose of β-Blocker	Trastuzumab	221	Noneffective	Both PFS and OS were worse when β-Blockers were used.	37359444
Colon cancer	β-Blockers	Atenolol (*n* = 1,370), bisoprolol (*n* = 1,591), metoprolol (*n* = 3,926), others (*n* = 1,185)	Clinical, retrospective cohort	Preoperative β-blocker users	Non-users	22,337	Effective	Significant reduction in 90-day mortality, 43% risk reduction in 1-year all-cause mortality, and reduced cancer-specific mortality up to 5 years.	32641361
Head and neck squamous cell carcinoma, NSCLC, melanoma, squamous cell carcinoma of the skin	β-Blockers	Carvedilol, metoprolol, propranolol, atenolol, labetalol, nadolol, nebivolol, and sotalol	Clinical, retrospective	β-Blocker users	Non-β-Blocker users	4,192	Noneffective	For head and neck squamous cell carcinoma: Worse OS and disease-free survival; trend for worse disease-specific survival. For NSCLC, melanoma, and squamous cell carcinoma of the skin: No significant effect on survival outcomes.	37285560
Gastric cancer	β-Blockers	Not mentioned	Clinical, retrospective	β-Blocker users	Non-β-Blocker users	361	Effective for diffuse gastric cancer, noneffective for intestinal gastric cancer	For diffuse gastric cancer: Improved OS and recurrence-free survival; independent prognostic factor. For intestinal gastric cancer: Worse OS; effect diminished after adjusting for cardiovascular risk profiles.	40131627
Colon cancer	β-Blockers	Metoprolol, atenolol, bisoprolol, and other β-blockers	Clinical, retrospective	Atenolol, bisoprolol and other β-blockers	Metoprolol	9,254	Noneffective	No significant difference in 90-day postoperative mortality between β-blocker types.	35347168
Epithelial ovarian cancer	β-Blockers	Not mentioned	Clinical, cohort study	β-Blocker users	Non-users	3,911	Effective	β-Blocker use associated with longer EOC-specific survival; 1.28 months longer survival at 5 years.	39865612
Breast cancer	β-Blockers	Not mentioned	Clinical, Cohort Study	β-Blocker users	Non-users	13,535	Effective in specific subtypes	In triple-negative breast cancer, β-blockers use associated with longer recurrence free interval and distant recurrence free interval; in luminal B HER2^+^, β-blockers use associated with longer recurrence free interval.	40215556
Ductal carcinoma in situ	β-Blockers	Not mentioned	Clinical, retrospective cohort study	β-Blocker users	Non-users	2,535	Effective	Dose-dependent reduced risk of invasive breast cancer progression.	38763971
Urothelial bladder cancer	β-Blockers	Not mentioned	Clinical, register-based cohort study	β-Blocker users	Non-users	16,669	Effective	Lower bladder cancer-specific mortality with nonselective β-blockers; strongest in locally advanced/metastatic disease.	35881046

NSCLC, non-small cell lung cancers; OS, overall survival; mCRC, metastatic colorectal cancer; interferon, IFN; PMID, PubMed Identifier; NCT numbers represent clinical trial registrations at ClinicalTrials.gov

Briefly, a Swedish nationwide cohort of 22,337 colon cancer patients showed reduced short- and long-term mortality with perioperative β-blocker therapy [269]. In epithelial ovarian cancer (*n* = 3,911), β-blocker use was associated with improved cancer-specific survival [270], and in urothelial bladder cancer (*n* = 16,669), β-blocker exposure correlated with reduced cancer-specific mortality [271]. Similarly, population-based studies in triple-negative breast cancer reported prolonged recurrence-free and distant metastasis-free survival among β-blocker users [272].

However, tumor specificity is evident. For example, retrospective analyses in head and neck squamous cell carcinoma have reported an association between β-blocker use and worse relapse-free survival, highlighting potential context-dependent effects [273]. Such heterogeneity likely reflects differences in tumor innervation patterns, receptor expression profiles, and systemic physiological effects of adrenergic blockade across tumor types.

Importantly, β-blockade has entered prospective evaluation, with several trials already showing positive clinical outcomes (Table 2). This progress is being bolstered by a surge of ongoing studies (e.g., NCT05741164 and NCT05651594) evaluating propranolol plus immune checkpoint inhibitors (ICIs) (see *Conjugate immunotherapy* section). Collectively, these data establish β-adrenergic antagonism as the most clinically mature neural-targeting strategy.

Mechanistically, β-adrenergic blockade interferes with stress-induced norepinephrine signaling, thereby suppressing tumor cell proliferation, angiogenesis, inflammation, and metastatic dissemination.

At the circuit level, tumor-associated sympathetic nerves release norepinephrine to activate β2-AR signaling in cancer cells and stromal compartments, promoting tumor growth and establishing feed-forward neurotrophic loops that reinforce tumor innervation and progression [50,88]. Pharmacological β-AR antagonists or genetic deletion of Adrb2 disrupts these circuits, impairs angiogenesis, and suppresses tumor progression across multiple cancer types.

At the cellular level, neurotransmitter blockade can restore sensitivity to programmed cell death and impair metastatic competence. At the metabolic level, adrenergic inhibition disrupts mitochondrial oxidative phosphorylation, reducing tumor energy reserve capacity and inducing combined energy depletion and oxidative stress. Notably, neurotransmitter inhibitors may also exhibit synergistic effects with targeted therapies, such as poly (adenosine diphosphate-ribose) polymerase (PARP) inhibitors, thereby overcoming therapy resistance [274].

Nebiprolol, a selective β1-AR-blocker suppresses oxidative phosphorylation in cancer cells by concurrently inhibiting mitochondrial complex I activity—through loss of NDUFS7 phosphorylation—and enhancing IF1-mediated inhibition of ATP synthase, thereby triggering a profound metabolic and oxidative stress crisis that limits the growth of colon and breast cancer [275]. Nonselective β-blocker propranolol reduces the viability, proliferation, and metastatic potential of CRC spheroids by disrupting hypoxia–adaptation programs and mitochondrial metabolism [276]. Propranolol may also reduce the incidence and invasiveness of oral cancer by inhibiting β-adrenergic signaling and suppressing downstream pro-tumorigenic pathway within the TME. In preclinical models, propranolol treatment was associated with decreased IL-6 and TNF-α levels, suggesting attenuation of inflammatory signaling that may contribute to reduced carcinogenesis and tumor aggressiveness [277]. Furthermore, β-blockers enhance the efficacy of conventional therapies, such as anthracyclines in triple-negative breast cancer, by blocking chemotherapy-induced β2-adrenergic signaling loops that promote metastatic dissemination [278].

Beyond established tumors, β-blockers also inhibit carcinogenesis and early micrometastatic niche formation of ovary cancer. Propranolol suppresses norepinephrine-driven β2-AR signaling to prevent anoikis resistance in fallopian tube epithelial precursor cells, thereby blocking the critical micrometastatic seeding of the ovary that initiates HGSOC [279].

At the microenvironmental level, neurotransmitter blockade also impairs tumor-associated angiogenesis. β1-selective blockade (e.g., nebivolol) suppresses endothelial proliferation and angiogenesis [275], while selective β2-AR antagonists prevent VEGFA-mediated angiogenesis and ultraviolet-B (UV-B)-induced cutaneous squamous cell carcinoma formation [280]. Consistently, endothelial-specific deletion of Adrb2 inhibits prostate tumor angiogenesis by reprogramming endothelial metabolism toward oxidative phosphorylation, further supporting a causal role for adrenergic signaling in vascular remodeling [88]. In parallel, propranolol decreases the expression of pro-tumorigenic signaling molecules, including Akt, NF-κB, and VEGF, thereby impairing the survival of OSCC cells [281].

In addition to adrenergic signaling, blockade of other neurotransmitter and neuropeptide pathways has revealed context-specific metabolic or survival dependencies in cancer. Cholinergic signaling exhibits tumor type-dependent effects: In gastric cancer, parasympathetic ACh activates M3 mAChRs to drive Wnt/YAP-dependent tumorigenesis, and vagotomy or M3 mAChR antagonists suppress tumor initiation and progression [52]; in contrast, activation of M1 mAChRs restrains PDAC growth by inhibiting MAPK/EGFR and PI3K/AKT signaling and reducing cancer stemness [152], underscoring the receptor- and context-specific nature of cholinergic modulation. The divergent outcomes of cholinergic signaling largely reflect receptor subtype-specific signaling programs and tissue-specific neural circuitry, which can differentially regulate epithelial proliferation, stemness, and immune responses.

Synaptic or pseudo-synaptic neurotransmission further creates actionable vulnerabilities. Glutamatergic signaling at nerve–tumor synapses activates tumoral NMDARs to promote calcium signaling, metabolic reprogramming, and tumor growth in PC and SCLC, while genetic or pharmacological disruption of NMDAR signaling significantly improves survival [56,57].

Neuropeptide signaling also contributes to nerve-driven oncogenic programs. Sensory nerve-derived CGRP promotes tumor growth and immune suppression in gastric and medullary thyroid carcinomas, whereas pharmacological blockade of CGRP receptors disrupts nerve–tumor signaling, restores antitumor immunity, and suppresses tumor progression [55,93] (Fig. 4B).

Finally, monoaminergic neurotransmitters modulate tumor survival and therapeutic response. Serotonin signaling sustains PCa cell survival, and antagonism of the 5-HT_7_ receptor (HTR7) induces apoptosis via NF-κB suppression and activation of CASP-3, CASP-9, BAX, and p53 pathways [282]. Dopaminergic signaling similarly influences treatment sensitivity, as DA D2 receptor antagonists enhance the efficacy of PARP inhibitor olaparib in endometrial cancer, with DA pathway activity correlating with tumor sensitivity to treatment [274].

Collectively, these findings demonstrate that targeting neurotransmitter and neuropeptide signaling—through receptor antagonists, inhibitors, or genetic blockade—can disrupt nerve-driven tumor growth, metabolic reprogramming, angiogenesis, and immune suppression, providing a strong mechanistic rationale for neuromodulatory strategies in cancer therapy.

However, not all neurotransmitter interventions yield beneficial oncologic outcomes. For example, acetylcholinesterase inhibitors have been associated with an increased risk of lung cancer in elderly patients [283]. These observations underscore the context-dependent nature of neurotransmitter signaling in cancer and highlight the necessity for tumor type- and pathway-specific precision when applying neuromodulatory therapeutic strategies. These examples highlight that the biological consequences of neurotransmitter signaling are highly receptor-, signaling-, and context-dependent.

Importantly, beyond receptor subtype expression, the signaling output of many neurotransmitter receptors is further shaped by the regulatory logic of G protein-coupled receptor (GPCR) signaling [284]. Neurotransmitter receptors—including adrenergic, muscarinic, serotonergic, and many neuropeptide receptors—can undergo dynamic regulation through mechanisms such as receptor desensitization, β-arrestin-mediated signaling, and biased agonism [284,285]. These processes allow the same receptor to activate distinct downstream pathways depending on ligand properties, stimulation patterns, and cellular context [286,287]. Consequently, chronic neural stimulation within the TME may reprogram receptor responsiveness and alter the balance between proliferative, metabolic, and immunomodulatory signaling outputs. A more detailed discussion of these mechanisms is provided in Supplementary File 3, where we summarize key regulatory principles of GPCR signaling relevant to neurotransmitter-driven tumor biology.

### Neurotrophic signaling targeting

Targeting neurotrophic signaling—particularly NGF, BDNF, and GDNF pathways—represents an upstream strategy to disrupt tumor innervation, PNI, and nerve–tumor feed-forward interactions (Fig. 4C). Unlike neurotransmitter blockade, which primarily interferes with signal transmission, neurotrophic targeting acts at the level of neuritogenesis/axonogenesis and neural remodeling, thereby limiting the structural integration of nerves into the TME.

Neurotrophic signaling plays a central role in establishing feed-forward loops between tumors and nerves. TrkA serves as the principal receptor for NGF, whereas TrkB is the major receptor mediating BDNF signaling [50,288] (Fig. 4C). Activation of these receptors promotes axonal sprouting, tumor cell survival, invasion, and resistance to stress, thereby coupling neural plasticity with malignant progression.

In PC, tumor cells stimulate sympathetic nerves through NGF and BDNF secretion, while norepinephrine released from nerves activates β2-AR signaling in tumor cells, further enhancing proliferation and neurotrophic factor production, thereby reinforcing tumor innervation and progression [50]. Interrupting this cycle through Trk inhibitors can markedly inhibit the progression of PC [50,289] (Fig. 4C). Similarly, in gastric cancer, ACh derived from Dclk1^+^ tuft cells and parasympathetic nerves induces epithelial NGF expression via M3 mAChR–YAP signaling, which in turn promotes cholinergic axon expansion and establishes a self-amplifying neurotrophic loop [52]. Disruption of Trk kinase activity effectively breaks this circuit and suppresses tumor progression [52].

Beyond NGF/TrkA signaling, BDNF–TrkB is frequently up-regulated in multiple solid tumors, including bladder and breast cancers. TrkB activation enhances proliferation, migration, invasion, resistance to anoikis, and angiogenesis, whereas TrkB-neutralizing antibodies or pathway blockade suppresses tumor cell growth and induces apoptosis (Fig. 4C). These findings establish BDNF/TrkB signaling as a promising therapeutic target in solid malignancies [288].

Neurotrophic signaling is also a key driver of Schwann cell-mediated neural remodeling. Tumors such as melanoma mimic nerve injury signals by releasing transforming growth factor-β (TGF-β) and IL-1β, activating the ERK/c-Jun pathway in Schwann cells and reprogramming them into repair-like, neurotrophin-secreting cells. These Schwann cells produce NGF, BDNF, IL-6, and matrix metalloproteinase-9 (MMP-9), remodel the extracellular matrix, and establish an immunosuppressive microenvironment that promotes tumor progression [34]. Importantly, NGF-neutralizing antibodies or local denervation disrupt this Schwann cell–tumor axis and markedly inhibit tumor growth, highlighting NGF blockade as a strategy to target tumor-associated neural remodeling.

In the context of PNI, neurotrophic signaling directly guides tumor cell migration toward nerves. TAMs secrete GDNF, which activates RET/GFRα1 signaling in cancer cells and drives nerve-directed migration, while soluble GFRα1 further amplifies RET activation [40,42]. Blockade of the GDNF–GFRα1–RET axis using GFRα1-targeting small interfering RNA (siRNA), anti-GFRα1 antibody, siRET, and/or the RET inhibitor PYP1 effectively suppresses PC PNI by preventing macrophage-derived GDNF from activating RET–ERK/AKT signaling [40,42] (Fig. 4C). Additionally, cancer cells can enhance this axis through exosomal signaling, as lncXIST-mediated up-regulation of GDNF activates RET–ERK signaling and promotes PNI. Targeting lncXIST/miR-211-5p/GDNF axis in cancer cell exosomes presents a novel therapeutic strategy for PNI and associated neuropathic pain [290]. These findings identify GDNF–RET signaling as a central driver of PNI and support RET and GFRα1 inhibitors or blockade of this pathway as anti-neuroinvasion strategies.

Neurotrophic pathways also integrate with tumor metabolism and stress adaptation. Under serine/glycine deprivation, peripheral nerves supply serine to PC cells, while tumor cells up-regulate NGF via the GCN2–eIF2α–ATF4 pathway, thereby enhancing nerve recruitment and forming a metabolic–neurotrophic feedback loop. Blocking compensatory neuronal innervation with the Trk–NGF inhibitor LOXO-101 further suppresses PDAC tumor growth [289]. Similarly, under endoplasmic reticulum (ER) stress, tumor-derived proBDNF activates neuronal TrkB/p75NTR signaling to promote neurite outgrowth and tumor innervation, whereas blocking the proBDNF–TrkB/p75NTR neurotrophic axis with an anti-proBDNF antibody suppresses ER stress-induced tumor innervation and slows PC progression [291].

At the level of metastatic competence, neurotrophic signaling enhances tumor cell survival and dissemination [292]. NGF signaling enhances invasion and metastasis via MAPK kinase (MEK)–ERK–MMP pathways, which can be effectively blocked by Trk inhibitors such as Larotrectinib [293]. Consistently, inhibition of TrkA/NGFR signaling suppresses proliferation, invasion, PNI, and cancer-associated pain in OSCC [294].

Targeting neurotrophic signaling has also been explored using advanced delivery systems. A novel nerve-homing nanocarrier, Lar@NP-OMVs, uses the neural binding peptide NP41 to deliver the Trk inhibitor larotrectinib directly to nerves within the TME, thereby inhibiting neurotrophic-driven nerve outgrowth. Meanwhile, the bacterial outer membrane vesicle (OMV) scaffold of Lar@NP-OMVs repolarizes M2-like TAMs toward an M1-like, nerve-damaging phenotype, thereby amplifying nerve suppression and enhancing the chemotherapeutic efficacy of gemcitabine in PC models [295]. This approach highlights the therapeutic potential of combining neurotrophic blockade with immune modulation.

Moving beyond preclinical discovery, the clinical validation of neurotrophic signaling has been spearheaded by Trk inhibition, which currently represents the most successful translation in this field. Larotrectinib achieved an objective response rate of 69% across 25 tumor types in pooled phase I/II trials (NCT02576431, NCT02122913, and NCT02637687, see 10.1200/JCO.2022.40.16_suppl.3100), leading to tissue-agnostic U.S. Food and Drug Administration (FDA) approval for neurotrophic receptor tyrosine kinase (NTRK) fusion-positive cancers. In pediatric NTRK fusion tumors, objective response rates reached 94% in infantile fibrosarcoma [296]. Similarly, entrectinib demonstrated a 57% response rate with durable intracranial activity in NTRK fusion-positive tumors [297]. Together, these data provide definitive proof-of-principle that targeting neurotrophic signaling yields durable clinical benefits in molecularly defined populations.

Collectively, neurotrophic signaling orchestrates tumor innervation, neural invasion, metabolic adaptation, and metastatic dissemination through multi-layered feed-forward interactions between cancer cells, nerves, and stromal components. Targeting these pathways—via Trk inhibitors, RET blockade, or NGF/BDNF neutralization—provides a mechanistically grounded strategy to disrupt the structural and functional integration of nerves within tumors.

### Conjugate immunotherapy

Cancer progression is sustained not only by tumor-intrinsic immune evasion but also by neural circuits that suppress antitumor immunity. Therefore, therapeutic strategies that simultaneously target neural signaling and immune checkpoint pathways represent a rational combinatorial approach to overcome resistance to immunotherapy. In this section, we focus exclusively on interventions that integrate neuromodulation with immunotherapy.

#### Neurotransmitter signaling blockade combined with immunotherapy

##### β-Adrenergic blockade and ICIs

As discussed in the sections *Long-distance neural regulation—Tumor hijacking of inter-organ nerve circuits* and *Psychological states*, stress-induced β-adrenergic signaling suppresses cytotoxic immune responses. Preclinical studies in melanoma, CRC, and breast cancer demonstrate that combining the β-blocker propranolol with anti-CTLA-4 therapy dramatically and synergistically potentiates its antitumor efficacy [298]. In cases of melanoma, propranolol was found to increase both the quantity and cytotoxicity of CD8^+^ T cells and NK cells, thereby potentiating immunotherapy responses [299].

Clinical observations support this neural–immune synergy. Retrospective analyses in non-SCLC (NSCLC) patients receiving ICIs demonstrated that concomitant β-blocker use was associated with improved progression-free survival [300] (Fig. 4D). Importantly, β-adrenergic blockade has now entered prospective evaluation. A phase I trial combining propranolol with pembrolizumab in advanced melanoma reported a 78% objective response rate without dose-limiting toxicity [301]. Subgroup analysis of the EORTC 1325/KEYNOTE-054 phase III trial further suggested improved recurrence-free survival among β-blocker users treated with adjuvant pembrolizumab [302].

Multiple phase II trials are currently underway to further validate this strategy, including NCT05741164 evaluating propranolol plus pembrolizumab in ICI-refractory triple-negative breast cancer, and NCT05651594 investigating the addition of propranolol to chemotherapy and pembrolizumab in advanced esophageal or gastroesophageal junction adenocarcinoma.

Collectively, these findings position β-adrenergic blockade as one of the most clinically advanced and translational neural–immune combinatorial strategies in oncology.

##### CGRP–sensory nerve targeting and immune checkpoint therapy

Tumors exploit sensory nerve–immune communication to induce systemic immune suppression. Under immune stress, tumor-derived SLIT2 activates nociceptors, increasing CGRP secretion in tumor-draining lymph nodes and suppressing antitumor immunity. Pharmacological blockade of the CGRP receptor restores immune function and markedly enhances the efficacy of immunotherapy while simultaneously alleviating cancer pain [76]. This represents a prototypical example of dual-action therapy in which sensory nerve blockade directly augments immunotherapy responsiveness.

Although CGRP receptor antagonists are currently approved for migraine prophylaxis, emerging translational evidence suggests potential oncologic relevance. Biomarker analysis from the MONSTAR-SCREEN-2 study (*n* = 1,475 advanced solid tumors) demonstrated that low RAMP1 expression was associated with improved progression-free survival under immune checkpoint blockade (see 10.1200/JCO.2025.43.16_suppl.2629), suggesting that CGRP pathway activity may influence immunotherapy responsiveness. However, dedicated interventional oncology trials targeting CGRP signaling have not yet been completed, and clinical validation remains an important future direction (see Supplementary File 2 for further discussion).

##### VIP signaling and PD-1 blockade

VIP-mediated signaling suppresses T cell activation within the PDAC TME. Combination therapy using VIP receptor antagonists and PD-1 inhibitors demonstrates a strong synergistic effect, resulting in long-term anticancer immune memory and effectively preventing tumor growth [303].

##### Cholinergic signaling and immune checkpoint regulation: Potential combination

Cholinergic signaling modulates immune checkpoint expression, as ACh-mediated activation of the M3 mAChR promotes PD-L1 and PD-L2 expression in CRC, indicating that cholinergic signaling inhibition could down-regulate these immunosuppressive ligands and potentially enhance the efficacy of immunotherapy [304].

#### Neurotrophic signaling targeting combined with immunotherapy

Neurotrophic signaling has recently emerged as another neural pathway capable of shaping antitumor immunity. Mechanistic studies show that tumor-derived NGF activates TrkA signaling to promote immune evasion. In melanoma models, NGF signaling suppresses IFN-γ responsiveness in tumor cells and impairs TCR signaling in effector T cells, thereby limiting cytotoxic immune activity. Genetic or pharmacological blockade of NGF–TrkA signaling, including treatment with the Trk inhibitor larotrectinib, restores sensitivity to immune checkpoint blockade and promotes durable memory T cell responses [305].

Clinical observations further support this neural–immune interaction. A pan-cancer analysis of 3,888 patients demonstrated that NTRK mutations are associated with improved responses to ICIs, including higher objective response rates and prolonged survival [306]. In NSCLC cohorts treated with ICIs, reduced TrkA signaling—through loss-of-function mutations or pharmacologic inhibition—correlates with increased T cell infiltration and enhanced immunotherapy efficacy [307].

Prospective trials directly combining TRK inhibitors with immune checkpoint blockade remain limited. Ongoing precision oncology studies such as the TAPISTRY trial (NCT04589845) are evaluating targeted therapies in molecularly selected tumors and may provide future evidence for such strategies. At present, most clinical insights derive from retrospective analyses or sequential treatment reports rather than dedicated combination trials [308,309].

#### Bioelectronic neuromodulation combined with immunotherapy

Bioelectronic neuromodulation has emerged as a neural–immune synergy strategy. Sciatic nerve stimulation (ST36) activates ProkR2^+^ sensory neurons and triggers DA release via the vago–adrenal axis, enhancing NK cell cytotoxicity. Neurostimulation also up-regulates tumor PD-L1 through IFN-γ signaling, increasing tumor sensitivity to checkpoint blockade. In triple-negative breast cancer models, the combination of sciatic nerve stimulation and anti-PD-1 therapy produces markedly greater tumor control than either treatment alone, overcoming immunoresistance [310].

#### Neuroimmune nanotherapeutic platforms: β-AR nanovaccine strategy

A tumor membrane-coated nanovaccine co-delivering CpG and the β-AR inhibitor propranolol enhances DC maturation, promotes CD8^+^ T cell priming, and alleviates the immunosuppressive TME. By integrating vaccination with β-adrenergic blockade, this biomimetic platform achieves superior prophylactic and therapeutic efficacy compared to vaccine alone, highlighting the potential of combining neural signaling inhibition with cancer immunotherapy [311].

Collectively, these studies demonstrate that neural signaling is not merely a passive component of the TME but an active regulator of immune responsiveness. Neurotransmitter blockade disrupts functional neural suppression, neurotrophic targeting limits structural tumor innervation, and neural–immune combinatorial strategies directly enhance checkpoint inhibitor efficacy. By integrating neuromodulation with immunotherapy, these approaches shift the therapeutic paradigm from tumor-centric targeting to circuit-level intervention, restoring systemic immune competence and overcoming resistance to conventional immunotherapy.

### Psychological therapies

Psychological stress represents a systemic modulator of tumor progression through neuroendocrine–immune mechanisms. Large-scale epidemiological studies have linked chronic psychological stress to increased cancer incidence, reduced survival, and elevated cancer mortality [312,313]. In breast cancer, both acute and chronic stress have been associated with an increased risk of recurrence [314].

The mechanisms by which psychological stress promotes tumor progression—through activation of the SAM axis, the HPA axis, and particularly through direct SNS innervation of the TME—have been discussed in detail in the section *Psychological states*.

Beyond biological mechanisms, psychosocial interventions demonstrate clinically meaningful benefits. Psychological therapies, particularly cognitive–behavioral stress management (CBSM), improve survival and reduce mortality risk in breast cancer patients while alleviating depression, anxiety, and stress-related symptoms [315–317] (Fig. 4E). Randomized controlled trials further show that group cognitive behavioral therapy enhances quality of life and emotional resilience in breast cancer patients. Given that animal models confirm stress promotes malignancy via direct SNS fibers rather than solely through systemic factors, psychological interventions might similarly leverage these neural tracks to suppress tumor progression. Whether positive emotions can dampen this intratumoral innervation remains a compelling frontier for clinical investigation.

### Alleviating cancer-associated neuropathic pain

In addition to tumor-directed interventions, alleviating cancer-associated neuropathic pain represents a critical therapeutic dimension within neural–tumor crosstalk, as recently emphasized by Liu et al. [318], who identified “harnessing sensory neuronal pathways for neuropathic pain control” as one of 9 emerging translational strategies for reprogramming neural–tumor interactions. Cancer-related neuropathic pain is highly prevalent and markedly reduces quality of life, particularly in malignancies characterized by dense neural infiltration or extensive bone metastasis, such as PC, PCa, and head and neck tumors [319–321].

As discussed in Pain, accumulating evidence demonstrates that cancer-associated pain is mechanistically coupled to tumor progression rather than being a passive by-product of tissue damage. For example, melanoma cells recruit and sensitize TRPV1^+^ nociceptors, leading to CGRP release that suppresses CD8^+^ T cell function through RAMP1 signaling and promotes T cell exhaustion [205]. Similarly, in PDAC, CAF-derived NGF activates TRPV1^+^ sensory neurons, which release CGRP to suppress NK cell recruitment via the CGRP–RAMP1 axis, forming a bidirectional nociceptor–stroma–immune loop that links pain severity with tumor aggressiveness [209]. Disruption of TRPV1^+^ neurons or blockade of CGRP signaling simultaneously attenuates tumor growth and alleviates pain, underscoring that nociceptive signaling constitutes a functional component of tumor–nerve–immune crosstalk rather than a secondary phenomenon.

Clinically, cancer-related pain is also closely associated with psychological stress and circadian disruption (including sleep fragmentation and insomnia), both of which are recognized risk factors for tumor progression [213,214] (see the sections: *Psychological states* and *Lifestyle behaviors: Potential impacts*). These observations suggest that sensory nerve dysregulation may integrate with systemic neuroendocrine alterations to further influence tumor behavior.

From a pathophysiological perspective, neuropathic pain in cancer arises from multiple, partially overlapping sources, including perineural inflammation, PNI, metastatic bone microenvironment remodeling, and CIPN (Fig. 4F). Perineural inflammation involves mast cell–neuron crosstalk, with mast cell enrichment in intrapancreatic nerves correlating with neuropathic abdominal pain in PC [322–326]. Inhibition of mast cell degranulation therefore represents a rational analgesic strategy. PNI-associated pain is further amplified by tumor-induced myelin disruption and macrophage infiltration, in which blockade of the CXCL2–CXCR2 axis reduces macrophage recruitment and mechanical allodynia [327–329]. Notably, macrophage-to-neuron-like transformation mediated by the TGF-β1/Smad3 pathway contributes to nociceptive signaling, and pharmacological inhibition of Smad3 exerts robust analgesic effects in preclinical models [327,330,331].

In bone metastasis, acidic microenvironments activate TRPV1^+^ sensory neurons and promote CGRP and SP release, thereby driving pain signaling [332–334]. Targeted interventions—including TRPV1 antagonists (e.g., SB366791), proton pump inhibition (bafilomycin A1), CGRP antagonists (CGRP8-37), and osteoclast-targeting agents such as sodium Danshensu—have demonstrated marked analgesic efficacy in preclinical models [335–338] (Fig. 4F). Additionally, CIPN represents a major dose-limiting toxicity of chemotherapy [339]. Agents such as duloxetine and mirogabalin remain first-line symptomatic treatments [340,341], while modulation of PD-1/PD-L1 signaling has been shown to attenuate neuropathic pain in preclinical settings [342,343].

Beyond conventional receptor antagonists and signaling inhibitors, emerging nanotechnology-based and bioelectronic strategies further extend this therapeutic landscape (Fig. 4F). Ferritin nanoparticles, dual-targeting iron oxide nanoparticles, Mg/Al layered double hydroxide nanoshells, TRPV1-targeted nanoplatforms, and NGF/TrkA-blocking nanosystems have demonstrated dual effects in suppressing tumor growth and alleviating nociceptive abnormalities [318]. Optogenetic modulation of nociceptors and dopaminergic circuits also reveals bidirectional interactions between pain signaling and tumor progression, suggesting that neuromodulation may simultaneously regulate immune responses and tumor behavior [318,344,345].

Collectively, these findings support the concept that neuropathic pain is not only a symptomatic consequence of cancer but also an integral component of nerve–tumor crosstalk, and that sensory nerve-targeted interventions may confer dual benefits—symptom relief and modulation of tumor-associated neural and immune remodeling, thereby potentially restraining tumor progression. However, rigorous clinical validation remains necessary to translate these mechanistic insights into standardized oncologic pain management protocols.

### Integrated therapeutic perspectives and clinical challenges

Collectively, strategies targeting nerve–tumor interactions—including denervation, neurotransmitter blockade, neurotrophic signaling targeting, neural–immune combination therapy, psychological interventions, and neuropathic pain-oriented sensory pathway modulation—represent a multi-level framework spanning structural, functional, immune, and systemic regulation. This conceptual organization aligns with recent frameworks proposing multidimensional reprogramming of neural–tumor crosstalk, including tumor suppression, immune enhancement, metastasis control, neuromodulation, and pain management [318]. However, multifaceted translational challenges remain.

Importantly, neural-targeting strategies are already entering clinical evaluation, underscoring their immediate translational relevance. Among them, β-adrenergic blockade represents the most clinically advanced approach, supported by large retrospective cohorts and early-phase combination trials with ICIs. A phase I study combining propranolol with pembrolizumab in advanced melanoma reported a 78% objective response rate without dose-limiting toxicity [301], and ongoing phase II trials (e.g., NCT05741164 and NCT05651594) are evaluating this strategy in triple-negative breast cancer and gastroesophageal malignancies. In parallel, Trk inhibitors targeting neurotrophic signaling have achieved tissue-agnostic regulatory approval in NTRK fusion-positive tumors, providing proof-of-principle that neural pathway targeting can yield durable clinical responses. Emerging biomarker analyses further suggest that sensory neuropeptide pathways, such as CGRP signaling, may influence immunotherapy responsiveness, although dedicated oncology trials remain forthcoming. Together, these developments indicate that neural–tumor targeting is transitioning from mechanistic insight to early clinical implementation.

Denervation approaches, such as vagotomy, suppress tumor growth by interrupting neural transmission but are irreversible and may impair essential physiological functions. Neurotransmitter inhibitors, including β-blockers, show promising associations with improved outcomes; however, most evidence derives from retrospective or small prospective studies, and large randomized clinical trials are still lacking. Neurotrophic targeting (e.g., NGF/Trk inhibition) depends on receptor expression patterns and tumor heterogeneity, leading to variable responses among patients. Neural–immune combination strategies have demonstrated strong preclinical efficacy, yet robust validation in large-scale clinical trials remains limited—particularly for immunologically cold tumors such as PC and PCa. Similarly, while sensory nerve-targeted pain interventions show substantial preclinical promise, clinical integration into oncology care pathways remains fragmented and requires standardized translational validation.

Psychological therapies, although noninvasive and systemically beneficial, face barriers related to accessibility, cost, and variability in patient responsiveness. In many regions, mental health resources remain limited, restricting the broad implementation of stress-management interventions. Finally, alleviating cancer-associated neuropathic pain serves as a critical adjunct to the aforementioned interventions.

In conclusion, while targeting nerve–cancer interactions offers a promising paradigm shift in oncology, successful clinical translation will require mechanistic refinement, patient stratification, and rigorous randomized clinical validation. Future research should aim to integrate neural, immune, and psychosocial dimensions into precision oncology frameworks.

## Conclusions, Limitations, and Perspectives

### Summary

Cancer neuroscience has emerged as a rapidly expanding interdisciplinary field, highlighting the TME as the primary arena in which tumor initiation, invasion, and metastasis unfold. This field has refined our understanding of TME heterogeneity by introducing key concepts such as PNI and the innervated niche, thereby redefining tumors as neurobiologically active ecosystems.

In this review, we traced the historical development of PNS–cancer interactions within cancer neuroscience. Two major research trajectories have driven the field: PNI and tumor neurogenesis/innervation. PNI research initially focused on the invasive propensity of cancer cells but has progressively expanded to encompass the cellular ecology of the PNI niche, particularly interactions among tumor cells, Schwann cells, and macrophages, and other stromal components. In contrast, studies of tumor innervation have centered on neural function, integrating principles of neuroscience with tumor biology and accelerating the maturation of cancer neuroscience as an emerging research field and distinct discipline. Reflecting this conceptual shift, the most recent update of the Hallmarks of Cancer framework incorporated nerve–cancer interactions for the first time, spanning both “Innervation” as an enabling phenotypic characteristic and “Neurons and their axonal projections” as hallmark-conveying cells of the TME [13].

**Fig. 5.**
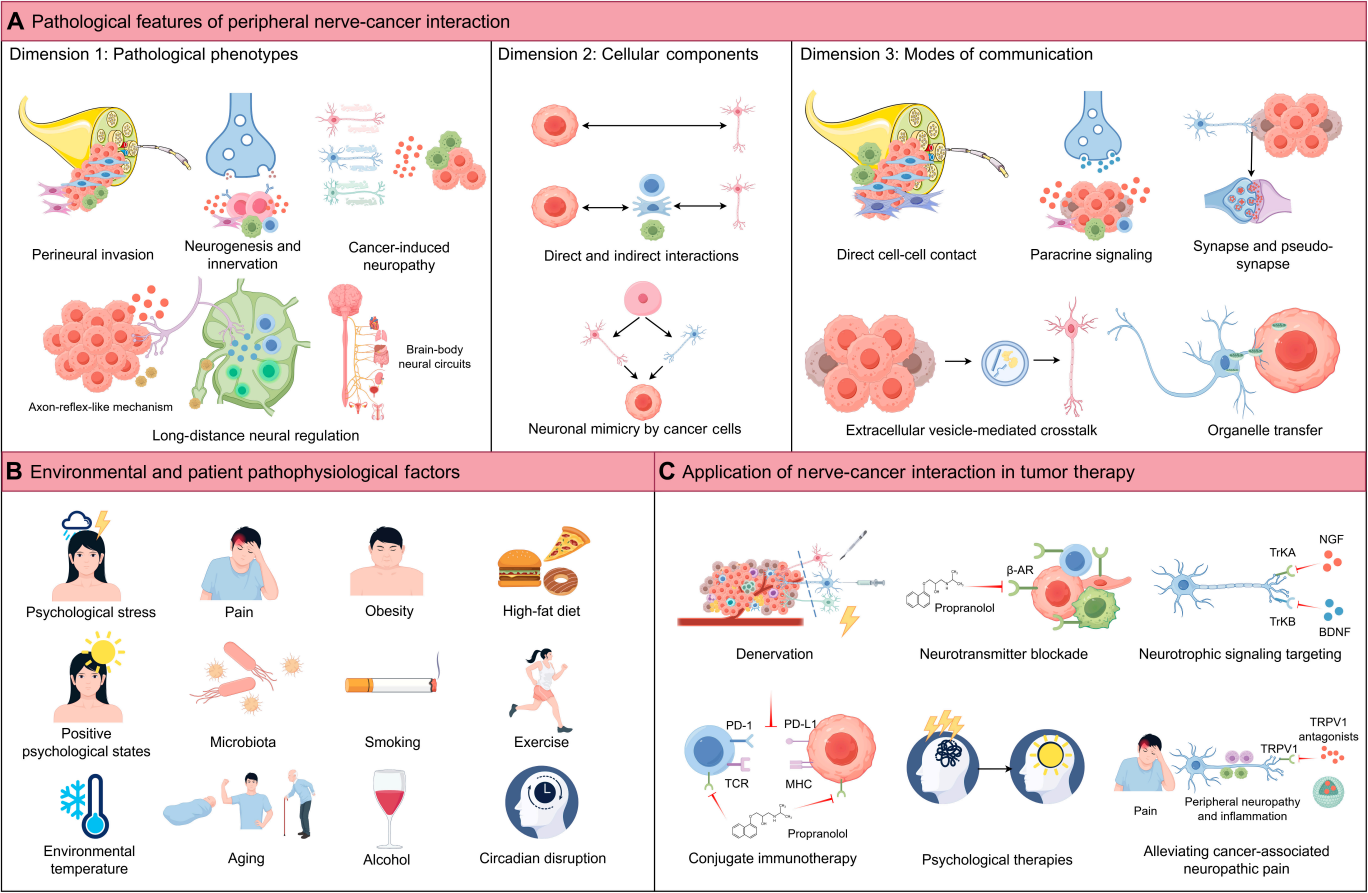
Conceptual framework of peripheral nerve–cancer interactions in the TME. (A) Pathological features of nerve–cancer interactions. In this review, we propose a 3-dimensional framework to systematically characterize interactions within the innervated niche among peripheral nervous system (PNS) components, tumor cells, and other TME constituents. The framework consists of 3 dimensions: (1) pathological phenotypes, (2) cellular components, and (3) modes of communication, which together provide an integrated organizational logic for understanding nerve–cancer crosstalk. (B) Modulators of nerve–cancer interactions. Environmental and patient-associated pathophysiological factors—including psychological states (both negative and positive), environmental stressors, pain, microbiota, aging, and obesity, as well as lifestyle behaviors such as smoking, diet, exercise, and circadian disruption—can significantly modulate nerve–cancer interactions within the TME and thereby influence cancer initiation and progression. (C) Therapeutic targeting of nerve–cancer interactions. Understanding nerve-cancer crosstalk provides opportunities for therapeutic intervention. Current and emerging strategies include denervation, neurotransmitter blockade, neurotrophic signaling targeting, neural–immune combinatorial therapy, psychological interventions, and approaches aimed at alleviating cancer-associated neuropathic pain.

Building on this foundation, we proposed and structured a 3-dimensional framework to systematically capture interactions within the innervated niche among PNS components, tumor cells, and other TME constituents (Fig. 5A):

1. Pathological phenotypes,
2. Cellular components,
3. Modes of communication.

Together, these dimensions provide an integrated organizational logic for studying nerve–cancer interactions.

Within dimension 1 (see the section *Dimension 1: Pathological phenotypes of nerve–cancer interactions in the TME*), we comprehensively summarized 4 major pathological phenotypes of PNS–cancer interaction. Beyond the classical and well-established phenotypes of PNI and tumor innervation, we incorporated cancer-induced neuropathy, a phenotype in which tumors remodel neural structure and function through inflammatory and metabolic mechanisms without direct neural invasion. We also highlighted long-distance neural regulation, whereby tumors hijack neural signaling and circuits across organs, extending PNS–cancer crosstalk beyond the local TME into systemic neural networks. These latter phenotypes await further characterization. We further propose that identifying tumor- and organ-specific phenotypic biases will be essential for precision targeting of nerve–cancer interactions.

In dimension 2 (cellular components, see the section *Dimension 2: Cellular components and mechanistic complexity in nerve–cancer crosstalk*), we emphasized the necessity of defining both the cellular sources and cellular targets of neural signals. Within the complex TME, it is critical to distinguish between direct nerve–tumor interactions and indirect signaling mediated by immune, glial, or stromal intermediates, as well as to clarify their directionality. Although neuronal mimicry by tumor cells is not universally regarded as a canonical nerve–cancer interaction, we argue that the ability of cancer cells to adopt neural communication modes warrants investigation using neuroscientific concepts and methodologies, and thus should be considered within the scope of cancer neuroscience.

Dimension 3 (modes of communication, see the section *Dimension 3: Modes of communication in nerve–cancer crosstalk*) addresses how neural and tumor cells interact mechanistically. Recent advances have demonstrated that nerves can form bona fide synapses and pseudo-synapses with tumor cells and can transfer mitochondria via TNTs—mechanisms that are themselves at the frontier of neuroscience. Integrating these discoveries, we propose a conceptual dichotomy between volume transmission (paracrine signaling and EV-mediated communication, characterized by diffuseness, low concentration, and sustained kinetics) and wiring transmission (synaptic and TNT-mediated communication, characterized by direct, point-to-point signaling with rapid onset and rapid termination, enabling high temporal precision).

We then reviewed PNS–cancer interactions across distinct peripheral neural subsystems (SNS, PSNS, ENS, and sensory system), using the 3-dimensional framework to organize phenotypes, distinguish between direct and indirect interactions, and delineate communication mechanisms across tumor types. To facilitate interpretation, we provided concise physiological overviews of each neural system. Notably, direct evidence of ENS-mediated tumor innervation remains limited (see the section *Nerve–cancer interactions in the ENS*); therefore, we additionally discussed ENS-associated PNI and cancer-induced neuropathy, as well as the unique roles of EGCs. Additionally, although many studies examine ENS neurotransmitters without definitive neuronal attribution, these findings remain highly informative for future investigations of ENS-driven tumor innervation and signaling.

Beyond the local TME, we analyzed environmental and patient-related pathophysiological factors (Fig. 5B), including psychological states, environmental stressors, pain, microbiota, aging, obesity, and lifestyle behaviors (e.g., smoking, diet, physical activity, and circadian disruption). Incorporating these factors situates nerve–cancer interactions within real-world physiological and environmental contexts, which will be indispensable for human clinical translational studies.

We further discussed therapeutic strategies targeting nerve–cancer interactions, encompassing denervation, modulation of neural signaling, synergistic integration with immunotherapy, psychological interventions, and neuropathic pain alleviation (Fig. 5C).

### Limitations, future directions, and challenges

Several critical gaps remain. Despite rapid progress in cancer neuroscience, several conceptual and methodological challenges continue to limit mechanistic interpretation and clinical translation. Distinct pathological phenotypes—including PNI, tumor innervation, cancer-induced neuropathy, and long-distance neural regulation—are often investigated as isolated phenomena, whereas growing evidence suggests that they may represent interconnected stages within a continuum of neural remodeling during tumor progression. However, most current studies still analyze these phenotypes independently, and a systematic framework integrating their temporal and functional relationships remains lacking. Addressing this gap will require integrated investigations into phenotypic crosstalk and functional neuroplasticity within the TME. For instance, Schwann cells, a primary driver of PNI, have recently been shown to directly modulate neural function by promoting sensory neuron excitability, suggesting that their role extends beyond structural support to active functional remodeling [346].

Furthermore, neural regulation of cancer should be explicitly mapped across distinct spatial scales, recognizing that the PNS functions not only as a terminal source of neurotransmitters but also as a sophisticated information processor. At the local level, research needs to decipher PNS circuits that operate through complex relays, axonal branching, and organ-specific reflexes—often functioning independently of central input. At present, however, experimental evidence for multi-organ neural circuits regulating tumor progression remains limited, and the generalizability of these findings across tumor types and physiological states has yet to be systematically established. Beyond these local interactions, the dimension of long-range brain–body neural circuits warrants exploration as a higher-order regulatory tier. This perspective encompasses bidirectional communication between the TME (or other target organs/tissues) and the CNS, where peripheral sensory inputs are integrated centrally to modulate systemic states that may, in turn, influence malignancy through autonomous outputs. Transitioning toward this multi-level understanding demands the incorporation of advanced neurobiological frameworks and multi-synaptic circuit-tracing technologies.

Future studies should move beyond single-nerve analyses to investigate multineural interactions within the TME, as multiple functionally distinct neuronal populations can concurrently innervate the same tumor niche. A major limitation of current studies is the coarse classification of neural inputs, which are often grouped simply as sympathetic, parasympathetic, or sensory pathways. However, emerging evidence indicates that substantial functional heterogeneity exists within these categories, including distinct neuronal subtypes with potentially opposing effects on tumor biology. The resulting phenotypes of tumor cells as well as surrounding epithelial, immune, and stromal cell populations are therefore likely shaped by the integrated output of these convergent neural signals—an aspect that may be particularly critical in the ENS, where dense and heterogeneous neural circuits coexist. Achieving this will require single-cell and spatially resolved approaches, including spatial transcriptomics and proteomics integrated with single-cell profiling of innervating ganglia. Technical challenges remain, particularly in segmenting linear neural fibers; emerging tools such as AxonFinder provide promising solutions for automated segmentation of tumor-innervating neuronal fibers [347].

Precise attribution of neurotransmitter sources, target cells, receptor subtypes, and dosage effects remains a major challenge in the field, as identical neurotransmitters or receptors can elicit divergent or even opposing phenotypes depending on cellular context, an issue particularly evident in ENS–cancer crosstalk (see the section *Nerve–cancer interactions in the ENS*). In many cases, neurotransmitters or neuropeptides detected within tumors may originate from multiple sources—including neurons, tumor cells, stromal cells, or infiltrating immune cells—making it difficult to attribute functional effects specifically to neural activity rather than non-neuronal neurotransmitter production. Finally, environmental and patient-level factors—including environment modulators, psychological states, metabolic states, aging, and lifestyle behaviors—must be systematically incorporated into future studies to advance clinical translation.

In this context, a deeper understanding of the receptor-encoding logic of neurotransmitter signaling—including GPCR desensitization, biased agonism, and signaling compartmentalization—may help explain the heterogeneous tumor responses to neural signals observed across tumor types and disease stages. Importantly, receptor expression alone does not necessarily predict signaling outcomes, as GPCR activity is dynamically shaped by receptor regulation, ligand-dependent signaling bias, and spatial organization of signaling complexes. Such mechanistic insight will be essential for the rational design of precision neuromodulatory therapies, enabling more selective targeting of receptor subtypes or signaling branches to modulate nerve–cancer interactions in a context-dependent manner.

Another important limitation lies in the heavy reliance on animal models, as neural innervation patterns, immune composition, and tumor evolution differ substantially between murine models and human cancers. Addressing these challenges will also require integration of advanced experimental platforms, including single-cell and spatial multi-omics, microfluidic and organoid models, nanotechnology-based sensors, and interdisciplinary collaboration, which will greatly expand our capacity to interrogate nerve–cancer interactions [348]. Microfluidic chip models provide physiologically relevant platforms to study innervation, neural invasion, and metastasis, exemplified by their application in modeling glioblastoma dissemination along cerebrospinal fluid pathways [349]. Organoid systems further recapitulate tumor architecture and neural interactions more faithfully than conventional 2-dimensional cell cultures and have been successfully applied to investigate neural signaling dependencies, such as studying the therapeutic effects of neurotransmitter pathway inhibitors on tumors [350,351]. Collectively, these approaches establish a robust experimental foundation for mechanistic studies and therapeutic development targeting nerve–cancer interactions.

Emerging neural technologies also enable dynamic monitoring of neurotransmitter activity within tumors, offering new avenues for diagnosis and treatment. Nanosensors such as graphene quantum dots allow sensitive detection of neurotransmitters including epinephrine and DA in cancer contexts [352,353], while optical nanoparticle probes and positron emission tomography (PET) imaging have been used to visualize DA release in human neuroblastoma cells in real time [354]. In addition, noninvasive 3.0-T magnetic resonance imaging (MRI)-based techniques permit selective detection and quantification of GABA, a neurotransmitter implicated in angiogenesis, immune evasion, invasion, and tumor proliferation [355]. Monitoring intratumoral GABA levels has prognostic value and may inform long-term therapeutic strategies, underscoring the translational potential of neurotransmitter-focused imaging in cancer neuroscience [356]. Despite promising experimental findings, clinical translation remains uneven across neuromodulatory strategies, with relatively few randomized controlled trials currently available to validate many of these approaches in cancer patients.
