# Supplementary material for: Peripheral Nerve–Cancer Interactions in the Tumor Microenvironment: A Three-Dimensional Framework Integrating Mechanisms, Modulators, and Therapeutic Strategies
Source: Research (Wash D C). 2026 Apr 1;9:1221. doi: 10.34133/research.1221 (PMC13040228; doi:10.34133/research.1221)
Supplement: Supplementary 1 — Supplementary Files 1 to 3 [file research.1221.f1.zip › Supplementary File 1.docx]

**Potential Direct and Indirect Interactions: ENS Signaling Molecules and Their Impact on Tumor and the TME**

Although direct studies of functional ENS tumor innervation remain limited (described above), extensive research on ENS signaling mediators (encompassing classical neurotransmitters, neuropeptides, and neurotrophic factors)—acting either directly on cancer cells or indirectly via other TME cell types—provides critical insights into potential ENS-cancer interactions. In the following section, we review the potential tumor-modulatory roles of these ENS signaling mediators, organized by their physiological functional categories. It should be noted upfront that, although these molecules are likely produced at least in part by the ENS, many of the studies discussed do not explicitly characterize the cellular source of the signaling molecule in question, or instead involve molecules derived from non-ENS components of the gut — such as neuroendocrine cells, tumor cells, or immune cells. Nevertheless, the phenotypic and mechanistic insights from these studies remain highly informative for defining future directions in ENS–cancer interaction research. When interpreting these findings, it is essential to distinguish neuronal sources (ENS vs. extrinsic innervation) from non-neuronal sources (neuroendocrine cells, tumor cells, immune cells, etc.), as many signaling molecules can be produced by multiple cell types. Importantly, in most cases discussed below, the available evidence does not allow unambiguous attribution of the observed tumor-modulatory effects specifically to ENS-derived signaling. Rather, these studies delineate signaling pathways and cellular responses that the ENS may potentially engage, directly or indirectly, within the gastrointestinal tumor microenvironment.

The following subsections are organized according to the physiological roles of ENS signaling mediators: **1) excitatory motor and secretomotor signaling, 2) inhibitory motor signaling, 3) diffuse neuromodulatory and sensory-associated signaling, 4) the endogenous opioid system, and 5) neurotrophic factors**. For each molecule, we first briefly describe its ENS origin and physiological function before discussing its known or putative roles in gastrointestinal tumorigenesis, with **potential tumor-modulatory effects color-coded in red (pro-tumorigenic) and blue (anti-tumorigenic)**. Readers seeking more comprehensive coverage of these signaling molecules interacting with gastrointestinal tumors are referred to dedicated reviews on this topic[1-5].

**1. Excitatory motor and secretomotor signaling**

The ENS initiates and maintains gut motility and secretion through excitatory signaling pathways that employ both fast ionotropic and slow metabotropic mechanisms.

Acetylcholine (ACh), a major and key excitatory neurotransmitter in the ENS, is primarily released by local enteric cholinergic neurons (excitatory motor neurons, secretomotor neurons, IPANs, interneurons) rather than extrinsic projections[1, 6, 7]. While vagal efferents modulate ENS circuits, they project mainly to enteric plexuses and do not directly innervate intestinal epithelial cells and most other non‑neuronal cells. Therefore, Ach in the gut is generally considered to originate primarily from intrinsic enteric cholinergic neurons[8]. ACh stimulates CRC progression via muscarinic receptor type 3 (mAChR3) through both direct (with cancer cells) and indirect interactions (with the TME): 1) mAChR3 antagonists attenuate proliferation[9], 2) ACh drives CRC cell proliferation and invasion via an MMP-7/HB-EGF/EGFR/ERK feed-forward loop, complemented by the synergistic activation of MMP-1/10 and p38 MAPK signaling[10-12], 3) mAChR3-deficient mice develop fewer/smaller tumors[13], and 4) in vivo blockade of mAChR3 restricts tumor growth and improves anti-cancer immunity by suppressing AKT/ERK-dependent angiogenesis and immunosuppression[14]. However, CRC cells themselves synthesize ACh and overexpress mAChR3, complicating neuronal versus autocrine distinctions[9]. Some studies have also provided supporting evidence that the ACh/α7-nAChR axis may promote CRC tumor cell proliferation[3, 15].

Beyond ACh, Substance P (SP) represents another critical excitatory neuropeptide derived from ENS excitatory motor neurons. It also functions as a key sensory neurotransmitter expressed in IPANs, while extrinsic sensory fibers from DRG also express SP, especially in pathological conditions[1, 7]. SP significantly accelerates CRC progression by enhancing cell proliferation via NK1R-mediated MAPK activation[16] and inducing immune cell proliferation[16]. Importantly, SP is also present in CRC cells themselves, complicating the distinction between neuronal and autocrine effects. Clinical evidence correlates SP and NK1R expression in cancer cells with increased metastasis and poor patient prognosis[17]. Pharmacological intervention using NK1R antagonists has been shown to inhibit tumor growth through the suppression of Wnt signaling[18-20].

Serotonin (5-HT) primarily serves excitatory functions in the ENS, with inhibitory roles in a minority of circuits. As noted above, intestinal 5-HT is predominantly derived from enterochromaffin (EC) cells — either secreted directly or reaching the gut via circulation — with only a minor fraction originating from myenteric interneurons[21]. Beyond the direct pro-tumor effects of ENS neuron-derived serotonin[22], extensive research on non-neuronal and systemic 5-HT sources provides critical insights into how this monoamine shapes CRC progression by directly act on tumor cells or modulating the TME. These findings underscore the profound complexity and context-dependency of 5-HT signaling. For instance, its impact may be concentration-dependent: while high doses promote mitogenesis, lower concentrations can paradoxically inhibit tumor growth by restricting intratumoral blood flow[4]. 5-HT has been shown to prevent DNA damage, thereby protecting against early-stage colorectal tumorigenesis[23]. However, 5‑HT promotes colorectal cancer invasion and metastasis by activating the 5‑HT1D receptor, which engages Axin1 and triggers β‑catenin/LEF/TCF4/MMP‑7 signaling to enhance tumor aggressiveness[24]. The immunomodulatory role of 5-HT is equally nuanced. Tumor‑derived 5‑HT activates HTR3A on macrophages to amplify NLRP3 inflammasome activation, thereby increasing IL‑1β production and driving colorectal cancer progression[25]. Elevated 5-HT drives chronic colonic inflammation by activating T‑ and B‑cell immune responses, thereby creating a microenvironment permissive for colorectal tumorigenesis[26]. Systemic depletion of peripheral serotonin has been reported to suppress tumor growth and improve survival by relieving circulating 5-HT-mediated inhibition of CD8⁺ T cells, thereby restoring their production of IFN-γ and granzyme B[27]. In contrast, within the TME, CD8⁺ T cells themselves serve as a localized 5-HT source, where the serotonin transporter (SERT) acts as a negative-feedback brake on their activation. Consequently, SERT inhibition via SSRIs can lead to local 5-HT accumulation, which—diverging from systemic effects—actually amplifies CD8⁺ T-cell antitumor activity through the 5-HTR–MAPK–TCR signaling axis[28]. This mechanism is consistent with a nationwide retrospective cohort study, showing SSRI can significantly reduce CRC risk among individuals with family history, especially for advanced-stage CRC[29]. Beyond receptor-mediated signaling, 5-HT can act through transglutaminase-dependent serotonylation to 1) activates the RhoA–ROCK–YAP pathway to drive cancer cell proliferation and tumorigenesis[30], and 2) reprogram CAFs (via serotonylation of small GTPases) into an inflammatory phenotype, which subsequently promotes CRC proliferation, invasion, and M2-like macrophage polarization[31]. To what extent ENS-derived neuronal 5-HT intersects with these diverse immune and stromal pathways remains a compelling, yet largely unexplored, frontier. Notably, the majority of mechanistic insights summarized above are derived from non-neuronal or systemic sources of serotonin, highlighting a substantial gap in directly linking ENS-originating 5-HT signaling to these tumor- and immune-modulatory effects in vivo.

**2. Inhibitory motor signaling**

Coordinated relaxation and descending inhibition are mediated by multiple inhibitory messengers acting through rapid diffusible signals, slow paracrine-like mechanisms, and modulatory pathways. In contrast to the roles of excitatory signals, inhibitory neurotransmitters and neuropeptides exhibit a more multifaceted impact on CRC.

Vasoactive intestinal peptide (VIP), predominantly from ENS inhibitory motor neurons, secretomotor neurons, and vasodilator neurons[1, 7, 32], promotes CRC cell proliferation via cAMP-Rap1/Ras-B-Raf-ERK signaling and stimulates primary tumor growth in vivo[33, 34]. VIP can also influence cancer through indirect interactions with TME cellular components. In human CRC samples, VPAC1 receptor is not only overexpressed in cancer cells with poor differentiation association but also enriched in tumor-associated macrophages and blood vessels[35]. VPAC1 antagonists inhibit the growth of colonic cancer cell lines in vitro[35]. VIP exerts immunosuppressive effects through VPAC2-mediated macrophage polarization toward a pro-tumor M2 phenotype, observed in both mouse models and clinical specimens, with VIP antagonists promoting anti-tumor M1 polarization[36]. Interestingly, VIP also exhibits anti-metastatic activity by attenuating invasiveness and angiogenesis[37-39].

Similarly, PACAP, a VIP-family neuropeptide in ENS inhibitory motor neurons and interneurons[1, 7, 32], stimulates proliferation and inhibit apoptosis via PAC1 receptor in vitro[40]. However, a protective role is suggested by the observation that PACAP-deficient mice develop CRC more rapidly[41] and that PACAP peptide levels are decreased in human colon cancer tissues[42].

Additionally, nitric oxide (NO), the principal ENS inhibitory neurotransmitter produced by nNOS⁺ neurons, is a key regulator of gastrointestinal motility and mucosal homeostasis[6]. Although no direct study has examined the carcinogenic role of nNOS⁺ enteric neurons specifically, accumulating evidence indicates that NO signaling critically modulates intestinal inflammation, stem/progenitor cell niches, and gastrointestinal tumorigenesis[43-45]. High endogenous NO production is a distinctive feature of colon cancer stem cells (CSCs), and iNOS-derived NO is essential for regulating their tumor-initiating properties; blockade of iNOS significantly reduces CSC tumorigenic capacity both in vitro and in vivo[46]. In gastric adenocarcinoma, iNOS expression correlates with tumor differentiation, clinical staging, lymph node metastasis, and poor 5-year survival, identifying iNOS as an independent prognostic factor[47]. Notably, alterations in the number or function of nNOS⁺ enteric neurons correlate with changes in motility and disease susceptibility[48], supporting the notion that ENS‑derived nitrergic signaling may contribute to shaping the TME.

γ-Aminobutyric acid (GABA) also functions as an inhibitory neurotransmitter in the enteric nervous system, although it is not a major transmitter compared with nitrergic or VIPergic pathways[49, 50]. The extent and mechanisms of GABAergic signaling in the ENS remain incompletely understood. Intrinsic GABAergic neurons are found mainly among enteric interneurons, and experimental data indicate that a small population of interneurons can release GABA within specific ENS circuits to modulate motility and secretory responses[51, 52]. It should be noted that most studies on GABA–tumor interactions involve non-ENS sources. GABA expression is elevated in colorectal cancer compared with normal colonic tissue and correlates with poor patient survival. Tumor‑derived GABA activates GABA_B‑mediated β‑catenin signaling to promote cancer cell proliferation and suppress intratumoral CD8⁺ T‑cell infiltration, and targeting GABA_B or GABA synthesis overcomes resistance to anti‑PD‑1 therapy in vivo[53]. Mouse and human B/plasma cell–derived GABA drives monocytes to differentiate into IL‑10–producing anti‑inflammatory macrophages that suppress CD8⁺ T‑cell cytotoxicity, and targeting B cells or their GABA‑synthesizing activity enhances anti‑tumor immunity[54]. Conversely, GABA also exhibits tumor-suppressive effects in certain contexts. Gut microbe Lactobacillus plantarum-derived GABA can suppress proliferation, migration, and invasion of 5‑FU‑resistant HT‑29 CRC cells by activating GABA_B receptors, which trigger apoptosis through inhibition of cAMP and cIAP2 signaling[55]. GABA suppresses norepinephrine‑induced migration of SW480 colon carcinoma cells by activating GABA_B receptors and lowering intracellular cAMP levels[56]. The GABA‑receptor agonist pentobarbital suppresses colon cancer cell proliferation and induces apoptosis in vitro, and markedly inhibits primary tumor growth and liver metastasis in vivo, in part through reducing intracellular cAMP, downregulating MMP‑2/MMP‑9 production, and decreasing tumor microvessel density[57]. GABA and GABA_B agonist baclofen also inhibits gastric carcinogenesis[58].

ATP serves as a complex purinergic neurotransmitter in the enteric nervous system. Inhibitory motor neurons of the myenteric plexus co‑release ATP together with nitric oxide (NO) and vasoactive intestinal peptide (VIP), and ATP can also be released from other classes of enteric neurons[59, 60]. Although ATP is co-released with NO and VIP by inhibitory motor neurons, its tumor-modulatory effects are mediated by distinct receptor subtypes (purinergic P2X7, P2Y2/6/12 receptors) on non-neuronal cells, and thus operate largely independently of its inhibitory neuromuscular role. In CRC, ATP activates P2X7 receptors to promote invasion and migration via STAT3 signaling, enhance proliferation and epithelial-mesenchymal transition (EMT) through PI3K/Akt/GSK-3β pathways, and upregulate multidrug resistance-associated protein 2 (MRP2) expression, conferring chemotherapy resistance[61-63]. P2Y receptors, particularly P2Y2, P2Y6, and P2Y12, are predominantly associated with colorectal cancer progression, while CD73 overexpression suppresses effector immune cell function[64]. In gastric cancer, ATP synthase F1β subunit (ATP5B) promotes metastasis and growth by activating FAK/AKT/MMP2 pathways and increasing extracellular ATP levels through P2X7 receptors[65]. P2Y2 receptor activation drives gastric cancer proliferation and metastasis via AKT/GSK-3β/VEGF signaling and facilitates EMT[66, 67]. These findings establish the ATP-purinergic receptor axis as a promising therapeutic target in gastrointestinal malignancies.

**3. Diffuse neuromodulatory and sensory-associated signaling**

Beyond direct motor control, the ENS employs diverse modulatory signals that integrate sensory input, autonomic commands, hormonal cues, and local microenvironmental changes to fine-tune gut function. Several of the signaling molecules discussed below do not conform neatly to classical excitatory or inhibitory categories, but instead act as context-dependent neuromodulators whose physiological and tumor-related effects vary across cellular targets and disease states.

Amongst **sensory integration signals**, calcitonin gene-related peptide (CGRP), presenting in IPANs, vasodilator neurons, secretomotor neurons, as well as in extrinsic sensory fibers from DRG — where it is more abundantly expressed than in the ENS[7, 32, 68, 69] — has been reported to suppress CRC cell motility and invasiveness[35].

Regarding **autonomic integration and global modulation**, neuropeptide Y (NPY) is predominantly derived from extrinsic sympathetic nerves (co-released with norepinephrine), with a minor fraction from ENS inhibitory motor neurons and secretomotor neurons[1, 7, 32]. While NPY suppresses CRC cell motility and invasiveness in vitro[39], NPY-deficient mice show reduced tumor cell proliferation, inflammation, and polyp size/number in vivo[70].

Somatostatin (SST), expressed in ENS secretomotor neurons and interneurons[1, 7, 71], inhibits CRC cell proliferation via SSTR3/SSTR5 receptors[72], with SST analogs suppressing Wnt/β-catenin signaling and inducing apoptosis[73-75]. To note, SSTR1⁺ neuroendocrine‑like cells within the TME also secrete SST, which restrains the proliferation and maintain the quiescence of colorectal cancer stem cells[76]. SST receptor expression negatively correlates with invasion/metastasis in human CRC and associates with better survival[77].

Galanin (GAL), derived from ENS secretomotor and vasodilator neurons and involved in neuroprotection, secretory inhibition, vasodilation, and injury response[1, 32, 78], exhibits a complex role in CRC. Clinically, the density of GAL-containing neurons in the myenteric plexus significantly increases near the tumor margin compared to distal regions[79], and elevated GAL levels in tissue or serum correlate with recurrence and poor prognosis[1]. Conversely, experimental evidence suggests potent anti-tumor properties: GAL administration reduces tumor lesion frequency in rat colon models, and its inclusion in triple therapy (with octreotide and serotonin) effectively shrinks both rat and human CRC xenografts[80].

Dopamine (DA) is a monoamine neurotransmitter released by a small subset of ENS neurons, where it inhibits motility and modulates local blood flow[81, 82]. In gastrointestinal tumors, DA mainly exhibits tumor-suppressive effects. Human colorectal cancer tissues exhibit reduced endogenous dopamine levels, decreased dopamine receptor expression, and diminished cAMP signaling[83]. DRD5 agonist SKF83959 suppresses CRC SW480 cell proliferation possibly by autophagic cell death[84]. In the TME, dopamine signaling through the DRD5 receptor promotes the differentiation of CD8⁺ T cells into CD103⁺ tissue‑resident memory T cells and enhances their antitumor activity[85]. In another mouse model, dopamine enhances the antitumor efficacy of 5‑fluorouracil by inhibiting VEGF‑driven angiogenesis through suppression of VEGFR‑2/MAPK/FAK signaling in tumor endothelial cells[86]. A similar pattern is observed in gastric cancer: gastric cancer tissues lack endogenous dopamine and the DA‑synthesizing enzyme tyrosine hydroxylase (compared with normal/benign gastric tissue), and exogenous dopamine suppresses gastric tumor growth by inhibiting VEGFR‑2 phosphorylation on DRD2–expressing tumor endothelial cells, thereby blocking angiogenesis[87].

Turning to neuropeptides involved in secretory and motor fine-tuning, gastrin-releasing peptide (GRP), primarily from ENS interneurons and secretomotor neurons with a small population from inhibitory motor neurons[1, 7, 71, 88], shows tumor-suppressive effects by inhibiting CRC cell invasiveness in vitro[89]. GRP/GRPR is also expressed in cancer cells, which is associated with well-differentiated tumor and correlated with better survival in human CRC[89-91].

Neuromedin U (NMU) is primarily produced by neurons in the submucosal plexus, particularly secretomotor/vasodilator neurons, where it acts as a co-transmitter to stimulate smooth muscle contraction and ion secretion[92-95]. NMU has emerged as a significant player in gastrointestinal tumorigenesis and metastasis. In CRC, NMU expression is significantly elevated in tumor tissues compared to adjacent normal tissue, and high NMUR1 expression correlates with shorter overall survival[96]. NMU acts through both autocrine and paracrine mechanisms: it induces an invasive phenotype in CRC cells via NMUR2 receptor activation, increasing cell motility, invasiveness, and prometastatic integrin expression[97]. Furthermore, NMU secreted by CRC cells promotes a tumor-supporting microenvironment by activating macrophages and endothelial cells through NMUR1, inducing pro-metastatic phenotypes and altered secretomes that enhance cancer cell migration[96]. NMU also contributes to radiation resistance in CRC via YAP/TAZ signaling activation, representing a potential mechanism of treatment failure[98]. Collectively, these findings position NMU as a potential biomarker of poor prognosis and a promising therapeutic target in CRC.

Peptide histidine isoleucine (PHI), a neuropeptide co-synthesized with VIP from a common precursor molecule, is abundantly present throughout the gastrointestinal tract, with the highest concentrations occurring in the colon[99, 100]. Regarding its potential role in gastrointestinal tumorigenesis, an experimental study[101] in rats demonstrated that PHI administration significantly enhanced azoxymethane-induced colon carcinogenesis. Rats receiving higher-dose PHI showed a significantly increased incidence of colon tumors compared to controls, and PHI also caused a significant increase in the labeling index of colon epithelial cells. These findings indicate that PHI may promote colon carcinogenesis through increasing proliferation of colon epithelial cells. Additionally, PHI has been shown to inhibit gastrin gene expression and gastrin release while stimulating somatostatin release in antral tissue, suggesting complex regulatory effects on gastric mucosal homeostasis that may have implications for gastric pathophysiology[102]. However, direct clinical studies examining PHI expression or function in human gastrointestinal tumors remain limited, and further investigation is warranted. It should also be noted that, despite being co-synthesized with VIP, the independent functional role of PHI within ENS neurons remains poorly characterized, and whether its potential tumor-modulatory effects are distinct from those of VIP has not been established. Given that available evidence derives solely from a single rat study, these findings warrant cautious interpretation.

**4. The endogenous opioid system**

The ENS also expresses a **complete endogenous opioid system**, comprising enkephalins (Met-ENK and Leu-ENK), dynorphins, and α-neoendorphin, which are distributed across various ENS neuronal subtypes and primarily function through neuromodulatory mechanisms to regulate visceral pain, motility, and secretion[103-105].

The endogenous opioid system, particularly Met-ENK, has been implicated in gastrointestinal tumorigenesis with complex and context-dependent effects. Methionine-enkephalin (Met-ENK), also termed opioid growth factor (OGF), demonstrates dual roles in colorectal cancer (CRC). On one hand, Met-ENK exerts anti-tumor effects by remodeling the tumor immune microenvironment — increasing M1-type macrophages, CD8⁺ and CD4⁺ T cell infiltration, downregulating immune checkpoints (PD-1, PD-L1, LAG3), and suppressing myeloid-derived suppressor cells (MDSCs)[106]. Met-ENK also directly inhibits cancer cell proliferation through the OGF receptor (OGFr), suppressing anchorage-independent growth in human colon cancer cells (HT-29) by targeting the cell cycle[107]. In gastric cancer, Met-ENK similarly inhibits tumor growth by skewing tumor-associated macrophages from M2 to M1 phenotype and blocking the PI3K/AKT/mTOR pathway[108]. More broadly, stimulation of both μ- and κ-opioid receptors exerts anti-inflammatory and anti-tumor activity in colitis-associated CRC models, decreasing tumor number and reducing β-catenin expression[109].

On the other hand, Met-ENK can paradoxically promote tumor progression. Met-ENK secreted by CRC cells is also reported to suppress tumor-infiltrating T lymphocytes via JNK-mediated apoptosis, facilitating immune escape, and its expression correlates with advanced Dukes' staging, nodal metastasis, and liver metastasis[110]. These opposing effects on T cells likely reflect differences in cellular source: whereas exogenous or neuron-derived Met-ENK may broadly enhance immune surveillance, Met-ENK secreted by CRC cells themselves may act locally within the TME to suppress tumor-infiltrating lymphocytes. However, differences in local concentration and receptor subtype engagement may also contribute, and further investigation is needed to clarify the underlying determinants.

Furthermore, CD10 (neutral endopeptidase) expressed by CRC cells degrades hepatic Met-ENK, abrogating its anti-tumor effect and thereby enhancing liver metastasis of CD10-positive CRC cells[111]. Collectively, these findings indicate that the endogenous opioid system plays multifaceted roles in gastrointestinal tumorigenesis — functioning as growth regulators, immune modulators, and potential therapeutic targets[112].

**5. Neurotrophic factors**

In addition to neurotransmitters and neuropeptides, **neurotrophic factors** may also play important roles in ENS–cancer interactions. Within the colonic ENS, NGF, BDNF, and NT-3 are secreted by enteric neurons, enteric glia, epithelial cells, and immune cells, and signal through p75 and Trk receptors (TrkA/B/C) to activate multiple pathways (e.g., MAPK/ERK, PI3K/Akt, and PLC-γ), thereby regulating cell proliferation, survival, and migration[1, 113].

The role of NGF in the ENS–cancer axis is supported by relatively direct functional evidence. Hayakawa et al. demonstrated that cholinergic stimulation induces NGF expression in gastric epithelium, and that NGF overexpression in turn promotes enteric nerve expansion and drives gastric carcinogenesis. In the context of CRC, NGF mRNA was found to be significantly upregulated in AOM–DSS-induced colorectal tumor tissue, suggesting that NGF may participate in inflammation-driven CRC development[114]. Importantly, this elevated NGF expression does not appear to be merely an epiphenomenon: in Vil1-Cre; R26-NGF mice, epithelial-specific overexpression of NGF was sufficient to induce dysplastic tumors in the rectum in the absence of any exogenous chemical or inflammatory stimulus. Furthermore, the tumor-promoting effect of NGF was functionally validated in the AOM–DSS chemical induction model, together indicating that NGF can drive CRC progression in both genetic and inflammation-driven contexts[114]. At the level of metastasis, high NGF expression is associated with increased metastatic incidence: NGF binding to TrkA induces TrkA phosphorylation, activating the MAPK/ERK pathway, which upregulates NGAL expression and enhances the activity of MMP2 and MMP9, thereby promoting CRC metastasis[115].

BDNF shares a similar downstream signaling logic with NGF but acts through the TrkB receptor. BDNF expression is elevated in CRC tissue and is associated with tumor growth, metastasis, and resistance to apoptosis[116]. BDNF/TrkB agonism promotes CRC cell proliferation and anti-apoptotic activity, particularly in advanced-stage tumors[117, 118]. Moreover, TrkB upregulation correlates with increased lymphatic vessel density and enhanced metastatic capacity[118], positioning the BDNF/TrkB signaling axis as a potential therapeutic target in CRC[119, 120].

Despite this evidence pointing to clear pro-tumorigenic functions of neurotrophic factors, precisely delineating their cellular sources and targets — encompassing enteric neurons, enteric glia, epithelial cells, cancer cells, and immune cells — remains a critical challenge in nerve–cancer interaction research. Such delineation is essential not only for determining the specific contribution of the ENS to tumor progression, but also for dissecting the underlying mechanisms through which neurotrophic factor signaling operates within the tumor microenvironment.

Collectively, the studies reviewed in this section highlight that, while many ENS-associated neurotransmitters, neuropeptides, and neurotrophic factors exert profound effects on tumor cells and the TME, direct functional evidence linking these effects specifically to ENS-derived neuronal activity remains limited. Future studies integrating genetic, anatomical, and functional approaches to resolve cell-type– and circuit-specific signaling will be essential to establish causal ENS–tumor interactions.

References

1. Godlewski J, Kmiec Z: Colorectal Cancer Invasion and Atrophy of the Enteric Nervous System: Potential Feedback and Impact on Cancer Progression. Int J Mol Sci. 2020; 21:9

2. Zhang Y, Chen J, Zhou Y, Jiang Y, Hu J, Liu X et al: Neuroimmune axis in gastrointestinal cancers: From mechanisms to therapeutic breakthrough. Cell Rep. 2025; 44:8

3. Zeng Z, Cai S, Ye C, Li T, Tian Y, Liu E et al: Neural influences in colorectal cancer progression and therapeutic strategies. Int J Colorectal Dis. 2025; 40:1

4. Schonkeren SL, Thijssen MS, Vaes N, Boesmans W, Melotte V: The Emerging Role of Nerves and Glia in Colorectal Cancer. Cancers (Basel). 2021; 13:1

5. Sun H, Wang T, Jiang X, Li M, He X, Ma Y et al: Integrating neuroscience and oncology: neuroimmune crosstalk in the initiation and progression of digestive system tumors. Mol Cancer. 2025; 24:1

6. Mazzoni M, Caremoli F, Cabanillas L, de Los Santos J, Million M, Larauche M et al: Quantitative analysis of enteric neurons containing choline acetyltransferase and nitric oxide synthase immunoreactivities in the submucosal and myenteric plexuses of the porcine colon. Cell Tissue Res. 2021; 383:2

7. Hansen MB: The enteric nervous system I: organisation and classification. Pharmacol Toxicol. 2003; 92:3

8. Bonaz B: Enteric neuropathy and the vagus nerve: Therapeutic implications. Neurogastroenterol Motil. 2025; 37:8

9. Cheng K, Samimi R, Xie G, Shant J, Drachenberg C, Wade M et al: Acetylcholine release by human colon cancer cells mediates autocrine stimulation of cell proliferation. Am J Physiol Gastrointest Liver Physiol. 2008; 295:3

10. Belo A, Cheng K, Chahdi A, Shant J, Xie G, Khurana S et al: Muscarinic receptor agonists stimulate human colon cancer cell migration and invasion. Am J Physiol Gastrointest Liver Physiol. 2011; 300:5

11. Said AH, Hu S, Abutaleb A, Watkins T, Cheng K, Chahdi A et al: Interacting post-muscarinic receptor signaling pathways potentiate matrix metalloproteinase-1 expression and invasion of human colon cancer cells. Biochem J. 2017; 474:5

12. Xie G, Cheng K, Shant J, Raufman JP: Acetylcholine-induced activation of M3 muscarinic receptors stimulates robust matrix metalloproteinase gene expression in human colon cancer cells. Am J Physiol Gastrointest Liver Physiol. 2009; 296:4

13. Raufman JP, Samimi R, Shah N, Khurana S, Shant J, Drachenberg C et al: Genetic ablation of M3 muscarinic receptors attenuates murine colon epithelial cell proliferation and neoplasia. Cancer Res. 2008; 68:10

14. Kuol N, Davidson M, Karakkat J, Filippone RT, Veale M, Luwor R et al: Blocking Muscarinic Receptor 3 Attenuates Tumor Growth and Decreases Immunosuppressive and Cholinergic Markers in an Orthotopic Mouse Model of Colorectal Cancer. Int J Mol Sci. 2022; 24:1

15. Hajiasgharzadeh K, Somi MH, Sadigh-Eteghad S, Mokhtarzadeh A, Shanehbandi D, Mansoori B et al: The dual role of alpha7 nicotinic acetylcholine receptor in inflammation-associated gastrointestinal cancers. Heliyon. 2020; 6:3

16. Mashaghi A, Marmalidou A, Tehrani M, Grace PM, Pothoulakis C, Dana R: Neuropeptide substance P and the immune response. Cell Mol Life Sci. 2016; 73:22

17. Chen XY, Ru GQ, Ma YY, Xie J, Chen WY, Wang HJ et al: High expression of substance P and its receptor neurokinin-1 receptor in colorectal cancer is associated with tumor progression and prognosis. Onco Targets Ther. 2016; 9

18. Rosso M, Robles-Frías MJ, Coveñas R, Salinas-Martín MV, Muñoz M: The NK-1 receptor is expressed in human primary gastric and colon adenocarcinomas and is involved in the antitumor action of L-733,060 and the mitogenic action of substance P on human gastrointestinal cancer cell lines. Tumour Biol. 2008; 29:4

19. Garnier A, Vykoukal J, Hubertus J, Alt E, von Schweinitz D, Kappler R et al: Targeting the neurokinin-1 receptor inhibits growth of human colon cancer cells. Int J Oncol. 2015; 47:1

20. Niu XL, Hou JF, Li JX: The NK1 receptor antagonist NKP608 inhibits proliferation of human colorectal cancer cells via Wnt signaling pathway. Biol Res. 2018; 51:1

21. Gershon MD: Evolution gone wrong: Enteric serotonergic neurons and colorectal cancer. Neuron. 2022; 110:14

22. Zhu P, Lu T, Chen Z, Liu B, Fan D, Li C et al: 5-hydroxytryptamine produced by enteric serotonergic neurons initiates colorectal cancer stem cell self-renewal and tumorigenesis. Neuron. 2022; 110:14

23. Sakita JY, Bader M, Santos ES, Garcia SB, Minto SB, Alenina N et al: Serotonin synthesis protects the mouse colonic crypt from DNA damage and colorectal tumorigenesis. J Pathol. 2019; 249:1

24. Sui H, Xu H, Ji Q, Liu X, Zhou L, Song H et al: 5-hydroxytryptamine receptor (5-HT1DR) promotes colorectal cancer metastasis by regulating Axin1/β-catenin/MMP-7 signaling pathway. Oncotarget. 2015; 6:28

25. Li T, Fu B, Zhang X, Zhou Y, Yang M, Cao M et al: Overproduction of Gastrointestinal 5-HT Promotes Colitis-Associated Colorectal Cancer Progression via Enhancing NLRP3 Inflammasome Activation. Cancer Immunol Res. 2021; 9:9

26. Chan YL, Lai WC, Chen JS, Tseng JT, Chuang PC, Jou J et al: TIAM2S Mediates Serotonin Homeostasis and Provokes a Pro-Inflammatory Immune Microenvironment Permissive for Colorectal Tumorigenesis. Cancers (Basel). 2020; 12:7

27. Schneider MA, Heeb L, Beffinger MM, Pantelyushin S, Linecker M, Roth L et al: Attenuation of peripheral serotonin inhibits tumor growth and enhances immune checkpoint blockade therapy in murine tumor models. Sci Transl Med. 2021; 13:611

28. Li B, Elsten-Brown J, Li M, Zhu E, Li Z, Chen Y et al: Serotonin transporter inhibits antitumor immunity through regulating the intratumoral serotonin axis. Cell. 2025; 188:14

29. Zhang N, Sundquist J, Sundquist K, Ji J: Use of Selective Serotonin Reuptake Inhibitors Is Associated with a Lower Risk of Colorectal Cancer among People with Family History. Cancers (Basel). 2022; 14:23

30. Yu H, Qu T, Yang J, Dai Q: Serotonin acts through YAP to promote cell proliferation: mechanism and implication in colorectal cancer progression. Cell Commun Signal. 2023; 21:1

31. Ling T, Dai Z, Wang H, Kien TT, Cui R, Yu T et al: Serotonylation in tumor-associated fibroblasts contributes to the tumor-promoting roles of serotonin in colorectal cancer. Cancer Lett. 2024; 600

32. Timmermans JP, Adriaensen D, Cornelissen W, Scheuermann DW: Structural organization and neuropeptide distribution in the mammalian enteric nervous system, with special attention to those components involved in mucosal reflexes. Comp Biochem Physiol A Physiol. 1997; 118:2

33. Alleaume C, Eychène A, Caigneaux E, Muller JM, Philippe M: Vasoactive intestinal peptide stimulates proliferation in HT29 human colonic adenocarcinoma cells: concomitant activation of Ras/Rap1-B-Raf-ERK signalling pathway. Neuropeptides. 2003; 37:2

34. Iishi H, Tatsuta M, Baba M, Okuda S, Taniguchi H: Enhancement by vasoactive intestinal peptide of experimental carcinogenesis induced by azoxymethane in rat colon. Cancer Res. 1987; 47:18

35. Iwasaki M, Akiba Y, Kaunitz JD: Recent advances in vasoactive intestinal peptide physiology and pathophysiology: focus on the gastrointestinal system. F1000Res. 2019; 8

36. Kittikulsuth W, Nakano D, Kitada K, Uyama T, Ueda N, Asano E et al: Vasoactive intestinal peptide blockade suppresses tumor growth by regulating macrophage polarization and function in CT26 tumor-bearing mice. Sci Rep. 2023; 13:1

37. Ogasawara M, Murata J, Kamitani Y, Hayashi K, Saiki I: Inhibition by vasoactive intestinal polypeptide (VIP) of angiogenesis induced by murine Colon 26-L5 carcinoma cells metastasized in liver. Clin Exp Metastasis. 1999; 17:4

38. Ogasawara M, Murata J, Ayukawa K, Saiki I: Inhibitory effect of vasoactive intestinal polypeptide (VIP) on experimental liver metastasis by murine colon 26-L5 carcinoma cells. Oncol Res. 1998; 10:7

39. Ogasawara M, Murata J, Ayukawa K, Saimi I: Differential effect of intestinal neuropeptides on invasion and migration of colon carcinoma cells in vitro. Cancer Lett. 1997; 116:1

40. Le SV, Yamaguchi DJ, McArdle CA, Tachiki K, Pisegna JR, Germano P: PAC1 and PACAP expression, signaling, and effect on the growth of HCT8, human colonic tumor cells. Regul Pept. 2002; 109:1-3

41. Nemetz N, Abad C, Lawson G, Nobuta H, Chhith S, Duong L et al: Induction of colitis and rapid development of colorectal tumors in mice deficient in the neuropeptide PACAP. Int J Cancer. 2008; 122:8

42. Szanto Z, Sarszegi Z, Reglodi D, Nemeth J, Szabadfi K, Kiss P et al: PACAP immunoreactivity in human malignant tumor samples and cardiac diseases. J Mol Neurosci. 2012; 48:3

43. de Oliveira GA, Cheng RYS, Ridnour LA, Basudhar D, Somasundaram V, McVicar DW et al: Inducible Nitric Oxide Synthase in the Carcinogenesis of Gastrointestinal Cancers. Antioxid Redox Signal. 2017; 26:18

44. Peñarando J, López-Sánchez LM, Mena R, Guil-Luna S, Conde F, Hernández V et al: A role for endothelial nitric oxide synthase in intestinal stem cell proliferation and mesenchymal colorectal cancer. BMC Biol. 2018; 16:1

45. Savidge TC: S-nitrosothiol signals in the enteric nervous system: lessons learnt from big brother. Front Neurosci. 2011; 5

46. Puglisi MA, Cenciarelli C, Tesori V, Cappellari M, Martini M, Di Francesco AM et al: High nitric oxide production, secondary to inducible nitric oxide synthase expression, is essential for regulation of the tumour-initiating properties of colon cancer stem cells. J Pathol. 2015; 236:4

47. Li LG, Xu HM: Inducible nitric oxide synthase, nitrotyrosine and apoptosis in gastric adenocarcinomas and their correlation with a poor survival. World J Gastroenterol. 2005; 11:17

48. Cairns BR, Jevans B, Chanpong A, Moulding D, McCann CJ: Automated computational analysis reveals structural changes in the enteric nervous system of nNOS deficient mice. Sci Rep. 2021; 11:1

49. Auteri M, Zizzo MG, Serio R: The GABAergic System and the Gastrointestinal Physiopathology. Curr Pharm Des. 2015; 21:34

50. Auteri M, Zizzo MG, Serio R: GABA and GABA receptors in the gastrointestinal tract: from motility to inflammation. Pharmacol Res. 2015; 93

51. Hyland NP, Cryan JF: A Gut Feeling about GABA: Focus on GABA(B) Receptors. Front Pharmacol. 2010; 1

52. Krantis A, Nichols K, Staines W: Neurochemical characterization and distribution of enteric GABAergic neurons and nerve fibres in the human colon. J Auton Nerv Syst. 1998; 68:1-2

53. Huang D, Wang Y, Thompson JW, Yin T, Alexander PB, Qin D et al: Cancer-cell-derived GABA promotes β-catenin-mediated tumour growth and immunosuppression. Nat Cell Biol. 2022; 24:2

54. Zhang B, Vogelzang A, Miyajima M, Sugiura Y, Wu Y, Chamoto K et al: B cell-derived GABA elicits IL-10(+) macrophages to limit anti-tumour immunity. Nature. 2021; 599:7885

55. An J, Seok H, Ha EM: GABA-producing Lactobacillus plantarum inhibits metastatic properties and induces apoptosis of 5-FU-resistant colorectal cancer cells via GABA(B) receptor signaling. J Microbiol. 2021; 59:2

56. Joseph J, Niggemann B, Zaenker KS, Entschladen F: The neurotransmitter gamma-aminobutyric acid is an inhibitory regulator for the migration of SW 480 colon carcinoma cells. Cancer Res. 2002; 62:22

57. Thaker PH, Yokoi K, Jennings NB, Li Y, Rebhun RB, Rousseau DL, Jr. et al: Inhibition of experimental colon cancer metastasis by the GABA-receptor agonist nembutal. Cancer Biol Ther. 2005; 4:7

58. Tatsuta M, Iishi H, Baba M, Nakaizumi A, Ichii M, Taniguchi H: Inhibition by gamma-amino-n-butyric acid and baclofen of gastric carcinogenesis induced by N-methyl-N'-nitro-N-nitrosoguanidine in Wistar rats. Cancer Res. 1990; 50:16

59. Ren J, Bertrand PP: Purinergic receptors and synaptic transmission in enteric neurons. Purinergic Signal. 2008; 4:3

60. Burnstock G: Purinergic signalling: from discovery to current developments. Exp Physiol. 2014; 99:1

61. Zhang WJ, Hu CG, Luo HL, Zhu ZM: Activation of P2×7 Receptor Promotes the Invasion and Migration of Colon Cancer Cells via the STAT3 Signaling. Front Cell Dev Biol. 2020; 8

62. Zhang WJ, Luo C, Huang C, Pu FQ, Zhu JF, Zhu ZM: PI3K/Akt/GSK-3β signal pathway is involved in P2X7 receptor-induced proliferation and EMT of colorectal cancer cells. Eur J Pharmacol. 2021; 899

63. Vinette V, Placet M, Arguin G, Gendron FP: Multidrug Resistance-Associated Protein 2 Expression Is Upregulated by Adenosine 5'-Triphosphate in Colorectal Cancer Cells and Enhances Their Survival to Chemotherapeutic Drugs. PLoS One. 2015; 10:8

64. Roliano GG, Azambuja JH, Brunetto VT, Butterfield HE, Kalil AN, Braganhol E: Colorectal Cancer and Purinergic Signalling: An Overview. Cancers (Basel). 2022; 14:19

65. Wang X, Chang X, He C, Fan Z, Yu Z, Yu B et al: ATP5B promotes the metastasis and growth of gastric cancer by activating the FAK/AKT/MMP2 pathway. Faseb j. 2021; 35:4

66. Dong CR, Hu DX, Liu SC, Luo HL, Zhang WJ: AKT/GSK-3beta/VEGF signaling is involved in P2RY2 activation-induced the proliferation and metastasis of gastric cancer. Carcinogenesis. 2023; 44:1

67. Reyna-Jeldes M, De la Fuente-Ortega E, Cerda D, Velázquez-Miranda E, Pinto K, Vázquez-Cuevas FG et al: Purinergic P2Y2 and P2X4 Receptors Are Involved in the Epithelial-Mesenchymal Transition and Metastatic Potential of Gastric Cancer Derived Cell Lines. Pharmaceutics. 2021; 13:8

68. Russell FA, King R, Smillie SJ, Kodji X, Brain SD: Calcitonin gene-related peptide: physiology and pathophysiology. Physiol Rev. 2014; 94:4

69. Sternini C, Anderson K: Calcitonin gene-related peptide-containing neurons supplying the rat digestive system: differential distribution and expression pattern. Somatosens Mot Res. 1992; 9:1

70. Jeppsson S, Srinivasan S, Chandrasekharan B: Neuropeptide Y (NPY) promotes inflammation-induced tumorigenesis by enhancing epithelial cell proliferation. Am J Physiol Gastrointest Liver Physiol. 2017; 312:2

71. Xue J, Askwith C, Javed NH, Cooke HJ: Autonomic nervous system and secretion across the intestinal mucosal surface. Auton Neurosci. 2007; 133:1

72. Colucci R, Blandizzi C, Ghisu N, Florio T, Del Tacca M: Somatostatin inhibits colon cancer cell growth through cyclooxygenase-2 downregulation. Br J Pharmacol. 2008; 155:2

73. Wang S, Bao Z, Liang QM, Long JW, Xiao ZS, Jiang ZJ et al: Octreotide stimulates somatostatin receptor-induced apoptosis of SW480 colon cancer cells by activation of glycogen synthase kinase-3β, A Wnt/β-catenin pathway modulator. Hepatogastroenterology. 2013; 60:127

74. Chen JS, Liang QM, Li HS, Yang J, Wang S, Long JW: Octreotide inhibits growth of colonic cancer SW480 cells by modulating the Wnt/P-catenin pathway. Pharmazie. 2009; 64:2

75. Hohla F, Buchholz S, Schally AV, Krishan A, Rick FG, Szalontay L et al: Targeted cytotoxic somatostatin analog AN-162 inhibits growth of human colon carcinomas and increases sensitivity of doxorubicin resistant murine leukemia cells. Cancer Lett. 2010; 294:1

76. Modarai SR, Opdenaker LM, Viswanathan V, Fields JZ, Boman BM: Somatostatin signaling via SSTR1 contributes to the quiescence of colon cancer stem cells. BMC Cancer. 2016; 16:1

77. Evangelou I, Petraki C, Msaouel P, Scorilas A, Sdrolia E, Padazi G et al: Immunohistochemical expression of somatostatin receptor subtypes 2 and 5 in colorectal cancer. Eur J Clin Invest. 2012; 42:7

78. Furness JB, Costa M, Rökaeus A, McDonald TJ, Brooks B: Galanin-immunoreactive neurons in the guinea-pig small intestine: their projections and relationships to other enteric neurons. Cell Tissue Res. 1987; 250:3

79. Godlewski J, Pidsudko Z: Characteristic of galaninergic components of the enteric nervous system in the cancer invasion of human large intestine. Ann Anat. 2012; 194:4

80. Lang R, Gundlach AL, Kofler B: The galanin peptide family: receptor pharmacology, pleiotropic biological actions, and implications in health and disease. Pharmacol Ther. 2007; 115:2

81. Li ZS, Pham TD, Tamir H, Chen JJ, Gershon MD: Enteric dopaminergic neurons: definition, developmental lineage, and effects of extrinsic denervation. J Neurosci. 2004; 24:6

82. Li ZS, Schmauss C, Cuenca A, Ratcliffe E, Gershon MD: Physiological modulation of intestinal motility by enteric dopaminergic neurons and the D2 receptor: analysis of dopamine receptor expression, location, development, and function in wild-type and knock-out mice. J Neurosci. 2006; 26:10

83. Basu S, Dasgupta PS: Decreased dopamine receptor expression and its second-messenger cAMP in malignant human colon tissue. Dig Dis Sci. 1999; 44:5

84. Leng ZG, Lin SJ, Wu ZR, Guo YH, Cai L, Shang HB et al: Activation of DRD5 (dopamine receptor D5) inhibits tumor growth by autophagic cell death. Autophagy. 2017; 13:8

85. Chen Y, Yan SM, Pu Z, Feng J, Tan L, Li Y et al: Dopamine Signaling Promotes Tissue-Resident Memory Differentiation of CD8+ T Cells and Antitumor Immunity. Cancer Res. 2022; 82:17

86. Sarkar C, Chakroborty D, Chowdhury UR, Dasgupta PS, Basu S: Dopamine increases the efficacy of anticancer drugs in breast and colon cancer preclinical models. Clin Cancer Res. 2008; 14:8

87. Chakroborty D, Sarkar C, Mitra RB, Banerjee S, Dasgupta PS, Basu S: Depleted dopamine in gastric cancer tissues: dopamine treatment retards growth of gastric cancer by inhibiting angiogenesis. Clin Cancer Res. 2004; 10:13

88. Pfannkuche H, Firzlaff U, Sann H, Reiche D, Schemann M: Neurochemical coding and projection patterns of gastrin-releasing peptide-immunoreactive myenteric neurone subpopulations in the guinea-pig gastric fundus. J Chem Neuroanat. 2000; 19:2

89. Tell R, Rivera CA, Eskra J, Taglia LN, Blunier A, Wang QT et al: Gastrin-releasing peptide signaling alters colon cancer invasiveness via heterochromatin protein 1Hsβ. Am J Pathol. 2011; 178:2

90. Carroll RE, Matkowskyj KA, Chakrabarti S, McDonald TJ, Benya RV: Aberrant expression of gastrin-releasing peptide and its receptor by well-differentiated colon cancers in humans. Am J Physiol. 1999; 276:3

91. Rivera CA, Ahlberg NC, Taglia L, Kumar M, Blunier A, Benya RV: Expression of GRP and its receptor is associated with improved survival in patients with colon cancer. Clin Exp Metastasis. 2009; 26:7

92. Timmermans JP, Scheuermann DW, Stach W, Adriaensen D, De Groodt-Lasseel MH: Distinct distribution of CGRP-, enkephalin-, galanin-, neuromedin U-, neuropeptide Y-, somatostatin-, substance P-, VIP- and serotonin-containing neurons in the two submucosal ganglionic neural networks of the porcine small intestine. Cell Tissue Res. 1990; 260:2

93. Furness JB, Pompolo S, Murphy R, Giraud A: Projections of neurons with neuromedin U-like immunoreactivity in the small intestine of the guinea-pig. Cell Tissue Res. 1989; 257:2

94. Howard AD, Wang R, Pong SS, Mellin TN, Strack A, Guan XM et al: Identification of receptors for neuromedin U and its role in feeding. Nature. 2000; 406:6791

95. Brighton PJ, Wise A, Dass NB, Willars GB: Paradoxical behavior of neuromedin U in isolated smooth muscle cells and intact tissue. J Pharmacol Exp Ther. 2008; 325:1

96. Przygodzka P, Soboska K, Sochacka E, Pacholczyk M, Braun M, Kassassir H et al: Neuromedin U secreted by colorectal cancer cells promotes a tumour-supporting microenvironment. Cell Commun Signal. 2022; 20:1

97. Przygodzka P, Sochacka E, Soboska K, Pacholczyk M, Papiewska-Pająk I, Przygodzki T et al: Neuromedin U induces an invasive phenotype in CRC cells expressing the NMUR2 receptor. J Exp Clin Cancer Res. 2021; 40:1

98. Sim MK, Park JE, Kim SR, Lee J, Kim EJ, Kim Y et al: Neuromedin U contributes to radiation resistance in colorectal cancer via YAP/TAZ signaling activation. Oncol Rep. 2023; 50:4

99. Yiangou Y, Christofides ND, Blank MA, Yanaihara N, Tatemoto K, Bishop AE et al: Molecular forms of peptide histidine isoleucine-like immunoreactivity in the gastrointestinal tract. Nonequimolar levels of peptide histidine isoleucine and vasoactive intestinal peptide in the stomach explained by the presence of a big peptide histidine isoleucine-like molecule. Gastroenterology. 1985; 89:3

100. Tatemoto K: PHI--a new brain-gut peptide. Peptides. 1984; 5:2

101. Iishi H, Tatsuta M, Baba M, Iseki K, Uehara H, Nakaizumi A: Enhancement by peptide histidine isoleucine of experimental carcinogenesis in the colon of rats induced by azoxymethane. Cancer Lett. 1995; 94:1

102. Wolfe MM, Chang R, Mailliard ME, Karnik PS: The effects of peptide histidine isoleucine on antral gastrin and somatostatin. Mol Cell Endocrinol. 1992; 84:1-2

103. Sternini C, Patierno S, Selmer IS, Kirchgessner A: The opioid system in the gastrointestinal tract. Neurogastroenterol Motil. 2004; 16 Suppl 2

104. Steele PA, Costa M: Opioid-like immunoreactive neurons in secretomotor pathways of the guinea-pig ileum. Neuroscience. 1990; 38:3

105. Corbett AD, McKnight AT, Kosterlitz HW: Tissue content of opioid peptides in the myenteric plexus-longitudinal muscle of guinea-pig small intestine. J Neurochem. 1988; 51:1

106. Wang X, Li S, Yan S, Shan Y, Wang X, Jingbo Z et al: Methionine enkephalin inhibits colorectal cancer by remodeling the immune status of the tumor microenvironment. Int Immunopharmacol. 2022; 111

107. Zagon IS, McLaughlin PJ: Opioid growth factor (OGF) inhibits anchorage-independent growth in human cancer cells. Int J Oncol. 2004; 24:6

108. Wang X, Jiao X, Meng Y, Chen H, Griffin N, Gao X et al: Methionine enkephalin (MENK) inhibits human gastric cancer through regulating tumor associated macrophages (TAMs) and PI3K/AKT/mTOR signaling pathway inside cancer cells. Int Immunopharmacol. 2018; 65

109. Zielińska M, Szymaszkiewicz A, Jacenik D, Schodel L, Sałaga M, Zatorski H et al: Cyclic derivative of morphiceptin Dmt-cyclo-(D-Lys-Phe-D-Pro-Asp)-NH2(P-317), a mixed agonist of MOP and KOP opioid receptors, exerts anti-inflammatory and anti-tumor activity in colitis and colitis-associated colorectal cancer in mice. Eur J Pharmacol. 2020; 885

110. Ohmori H, Fujii K, Sasahira T, Luo Y, Isobe M, Tatsumoto N et al: Methionine-enkephalin secreted by human colorectal cancer cells suppresses T lymphocytes. Cancer Sci. 2009; 100:3

111. Kuniyasu H, Luo Y, Fujii K, Sasahira T, Moriwaka Y, Tatsumoto N et al: CD10 enhances metastasis of colorectal cancer by abrogating the anti-tumoural effect of methionine-enkephalin in the liver. Gut. 2010; 59:3

112. Fichna J, Janecka A: Opioid peptides in cancer. Cancer Metastasis Rev. 2004; 23:3-4

113. Liu S: Neurotrophic factors in enteric physiology and pathophysiology. Neurogastroenterol Motil. 2018; 30:10

114. Hayakawa Y, Sakitani K, Konishi M, Asfaha S, Niikura R, Tomita H et al: Nerve Growth Factor Promotes Gastric Tumorigenesis through Aberrant Cholinergic Signaling. Cancer Cell. 2017; 31:1

115. Lei Y, He X, Huang H, He Y, Lan J, Yang J et al: Nerve growth factor orchestrates NGAL and matrix metalloproteinases activity to promote colorectal cancer metastasis. Clin Transl Oncol. 2022; 24:1

116. Huang SM, Lin C, Lin HY, Chiu CM, Fang CW, Liao KF et al: Brain-derived neurotrophic factor regulates cell motility in human colon cancer. Endocr Relat Cancer. 2015; 22:3

117. Brunetto de Farias C, Rosemberg DB, Heinen TE, Koehler-Santos P, Abujamra AL, Kapczinski F et al: BDNF/TrkB content and interaction with gastrin-releasing peptide receptor blockade in colorectal cancer. Oncology. 2010; 79:5-6

118. Yu Y, Zhang S, Wang X, Yang Z, Ou G: Overexpression of TrkB promotes the progression of colon cancer. Apmis. 2010; 118:3

119. Mazouffre C, Geyl S, Perraud A, Blondy S, Jauberteau MO, Mathonnet M et al: Dual inhibition of BDNF/TrkB and autophagy: a promising therapeutic approach for colorectal cancer. J Cell Mol Med. 2017; 21:10

120. Blondy S, Christou N, David V, Verdier M, Jauberteau MO, Mathonnet M et al: Neurotrophins and their involvement in digestive cancers. Cell Death Dis. 2019; 10:2
