# Supplementary material for: Peripheral Nerve–Cancer Interactions in the Tumor Microenvironment: A Three-Dimensional Framework Integrating Mechanisms, Modulators, and Therapeutic Strategies
Source: Research (Wash D C). 2026 Apr 1;9:1221. doi: 10.34133/research.1221 (PMC13040228; doi:10.34133/research.1221)
Supplement: Supplementary 1 — Supplementary Files 1 to 3 [file research.1221.f1.zip › Supplementary File 2.docx]

**Review and Discussion of Clinical Trials on Beta Blockers and CGRP Inhibitors in Tumor Therapy**

**Beta Blockers**

Numerous national cohort studies and retrospective analyses have investigated the efficacy and survival impact of beta-blockers across various cancer types. A 10-year retrospective cohort study conducted in Sweden, which included 22,337 patients, revealed that those who received preoperative beta-blocking therapy exhibited significantly lower short- and long-term mortality rates following elective colon cancer surgery[1]. Additionally, a study involving 3,911 patients indicated that the use of beta-blockers was associated with improved cancer-specific survival in patients with epithelial ovarian cancer, potentially prolonging overall survival time[2]. Furthermore, another study encompassing 16,669 patients demonstrated that the use of beta-blockers correlated with a reduced cancer-specific mortality rate in patients with urothelial bladder cancer, with the protective effect being most pronounced in those with locally advanced or metastatic disease[3]. In a study involving 13,535 patients with triple-negative breast cancer, the use of beta-blockers was significantly associated with longer recurrence-free survival and distant metastasis-free survival, while the risk of breast cancer-specific death exhibited a decreasing trend. Similarly, among patients with Luminal B HER2+ breast cancer, beta-blocker usage was also significantly correlated with an extended recurrence-free survival period [4]. Conversely, a retrospective study that included 4,192 patients with head and neck squamous cell carcinoma, non-small-cell lung cancer, melanoma, and squamous cell carcinoma of the skin revealed that, in patients with head and neck squamous cell carcinoma, the use of beta-blockers was linked to poorer survival outcomes and diminished treatment responses. Notably, this adverse effect was not observed in patients with non-small-cell lung cancer, melanoma, or squamous cell carcinoma of the skin, indicating that the efficacy of beta-blockers may be dependent on the type of cancer[5]. A study involving 14,976 patients further demonstrated that the use of beta-blockers exhibited a time-dependent effect in breast cancer patients: initial use may be associated with an increased risk of breast cancer-specific death, while long-term use may correlate with a reduced risk of breast cancer-specific death[6]. For patients with ductal carcinoma in situ, a retrospective study including 2,535 female patients found that the use of beta-blockers may be linked to a reduced risk of progression to invasive breast cancer in a dose-dependent manner; however, this effect did not reach statistical significance[7]. Concerning the relationship between the types of beta-blockers and their therapeutic effects, a national retrospective study involving 9,254 patients indicated that different types of beta-blockers (such as metoprolol, atenolol, and bisoprolol) exhibited no significant differences in the postoperative mortality rate 90 days after colon cancer surgery[8]. In the treatment of infantile hemangioma, a retrospective study involving 445 patients found that propranolol and atenolol exhibited similar incidences of rebound growth; however, the atenolol group required a shorter treatment duration following rebound growth[9]. A prospective randomized clinical trial with 377 children diagnosed with infantile hemangioma further demonstrated no significant differences between atenolol and propranolol regarding initial response success rate, quality of life scores, complete ulcer healing time, and recurrence rates. Notably, the incidence of adverse events associated with atenolol was lower[10]. In conclusion, the effects of beta-blockers vary significantly across different cancer types and clinical contexts. Their efficacy may be influenced by multiple factors, including cancer type, duration of use, and specific drug. Future research should further explore individualized application strategies for these medications.

The efficacy differences of beta-blockers in different tumors show contradictory results. First, tumor type specificity plays a crucial role: the TME and signaling pathways of different tumors may significantly influence the efficacy of beta-blockers. For instance, the outcomes of beta-blocker treatment vary between head and neck squamous cell carcinoma and melanoma[5]**.** For example, the relative contributions of perineural invasion versus innervation/neurogenesis vary substantially across cancer types, likely reflecting differences in organ innervation density, tumor biology, and microenvironmental characteristics. Recognition of these distinct patterns is crucial for developing tailored therapeutic approaches: denervation strategies or neurotransmitter receptor blockade may be most effective in cancers with prominent innervation (such as pancreatic and breast cancers), while interventions targeting perineural invasion mechanisms may be more appropriate for cancers like esophageal squamous cell carcinoma with high perineural invasion rates. Second, individual differences among patients, including genetic background, comorbidities, and treatment history, may also affect the efficacy of beta-blockers. Third, the mechanisms of drug action are relevant; beta-blockers may exert their effects through various pathways, such as inhibiting the epinephrine signaling pathway, regulating the immune microenvironment, or suppressing angiogenesis, though the significance of these mechanisms may differ across tumor types. Finally, differences in research design, including variations between retrospective and prospective studies in data collection, sample size, and follow-up duration, can lead to inconsistent results. Future research should focus on stratified analyses based on tumor type, stage, and molecular characteristics to identify populations that may benefit from beta-blocker treatment

**CGRP Inhibitors**

CGRP and its receptors are pivotal in the activation of sensory nerves and the transmission of pain signals. However, there are currently very few registered clinical trials investigating CGRP inhibitors for cancer treatment. Additionally, robust preclinical evidence supporting their advancement into clinical trials for tumor management is lacking. In the context of nerve–cancer interactions, alternative pathways, such as beta-blockers, have garnered more attention. Notably, some preclinical studies suggest that targeting CGRP may inhibit tumor progression or modulate immune responses in specific models. In 2025, Zhang et al. demonstrated that, under immune stress, tumor cells secrete SLIT2 to activate pain neurons within the TME. These activated pain neurons subsequently stimulate pain neurons in tumor-draining lymph nodes via neural signal transduction, which increases the secretion of CGRP, thereby inducing an immunosuppressive state. The use of CGRP receptor inhibitors, commonly employed in migraine treatment, to block the communication between tumors and tumor-draining lymph nodes mediated by sensory nerves can significantly enhance the efficacy of immunotherapy and alleviate cancer pain, achieving a dual effect of tumor suppression and analgesia[11].In the future, clinical trials will be conducted to evaluate the potential role of CGRP inhibitors in specific types of cancer; however, this endeavor will necessitate increased support from basic research.

**References**

1. Ahl R, Matthiessen P, Sjölin G, Cao Y, Wallin G, Ljungqvist O et al: Effects of beta-blocker therapy on mortality after elective colon cancer surgery: a Swedish nationwide cohort study. BMJ Open. 2020; 10:7

2. Löfling LL, Støer NC, Sloan EK, Nafisi S, Fortner RT, Botteri E: Beta-blockers and epithelial ovarian cancer survival: A Norwegian population-based cohort study. Int J Cancer. 2025; 157:1

3. Udumyan R, Botteri E, Jerlstrom T, Montgomery S, Smedby KE, Fall K: Beta-blocker use and urothelial bladder cancer survival: a Swedish register-based cohort study. Acta Oncol. 2022; 61:8

4. Scott OW, Tin Tin S, Botteri E: Beta blocker use and breast cancer survival by subtypes: A population-based cohort study. Breast. 2025; 81

5. Chen HY, Zhao W, Na'ara S, Gleber-Netto FO, Xie T, Ali S et al: Beta-Blocker Use Is Associated With Worse Relapse-Free Survival in Patients With Head and Neck Cancer. JCO Precis Oncol. 2023; 7

6. Scott OW, Tin Tin S, Elwood JM, Cavadino A, Habel LA, Kuper-Hommel M et al: Post-diagnostic beta blocker use and breast cancer-specific mortality: a population-based cohort study. Breast Cancer Res Treat. 2022; 193:1

7. Strell C, Smith DR, Valachis A, Woldeyesus H, Wadsten C, Micke P et al: Use of beta-blockers in patients with ductal carcinoma in situ and risk of invasive breast cancer recurrence: a Swedish retrospective cohort study. Breast Cancer Res Treat. 2024; 207:2

8. Ekestubbe L, Bass GA, Forssten MP, Sjölin G, Cao Y, Matthiessen P et al: Pharmacological differences between beta-blockers and postoperative mortality following colon cancer surgery. Sci Rep. 2022; 12:1

9. Baruch S, Ben Amitai D, Friedland R: Rebound Growth of Infantile Hemangiomas after Propranolol versus Atenolol Treatment: A Retrospective Study. Dermatology. 2024; 240:5-6

10. Ji Y, Chen S, Yang K, Zhang X, Zhou J, Li L et al: Efficacy and Safety of Propranolol vs Atenolol in Infants With Problematic Infantile Hemangiomas: A Randomized Clinical Trial. JAMA Otolaryngol Head Neck Surg. 2021; 147:7

11. Zhang Y, Guo Y, Liu Z, Sun Y, Yang X, Chen M et al: Cancer cells co-opt an inter-organ neuroimmune circuit to escape immune surveillance. Cell. 2025; 188:24
