# Supplementary material for: Peripheral Nerve–Cancer Interactions in the Tumor Microenvironment: A Three-Dimensional Framework Integrating Mechanisms, Modulators, and Therapeutic Strategies
Source: Research (Wash D C). 2026 Apr 1;9:1221. doi: 10.34133/research.1221 (PMC13040228; doi:10.34133/research.1221)
Supplement: Supplementary 1 — Supplementary Files 1 to 3 [file research.1221.f1.zip › Supplementary File 3.docx]

**GPCR signaling logic in neurotransmitter-driven tumor biology**

Many neurotransmitter receptors implicated in nerve-cancer interactions belong to the G protein-coupled receptor (GPCR) superfamily, including adrenergic, muscarinic, serotonergic, and several neuropeptide receptors. Increasing evidence indicates that GPCR signaling outputs are not fixed but are dynamically regulated through multiple mechanisms, including receptor desensitization, internalization, biased agonism, and spatial signaling compartmentalization[1-3]. Importantly, these receptor-level regulatory mechanisms interact with additional contextual variables—including ligand source, local concentration, stimulation dynamics, and tissue compartment—to shape the net biological outcome of neurotransmitter signaling within the tumor microenvironment. Consequently, identical neurotransmitters can produce divergent or even opposing phenotypes depending on receptor subtype engagement, cellular targets, ligand availability, and neural circuit context.

Upon sustained neurotransmitter exposure, GPCRs frequently undergo phosphorylation by G protein-coupled receptor kinases (GRKs), followed by recruitment of β-arrestins[4]. β-arrestin recruitment promotes receptor desensitization and receptor internalization while also scaffolding alternative signaling complexes. Importantly, downstream pathways such as ERK/MAPK can be activated through both G-protein-dependent and β-arrestin-dependent mechanisms, which differ in signaling kinetics and subcellular localization, thereby generating distinct functional outputs. In tumor contexts, chronic neural stimulation—for example prolonged adrenergic signaling during stress—may therefore reshape receptor responsiveness and alter the balance between proliferative, metabolic, inflammatory, and immune regulatory signaling outputs [5, 6]. **These regulatory dynamics help explain why adrenergic signaling frequently promotes tumor progression across multiple biological layers—including angiogenesis, immune modulation, and metabolic reprogramming—while pharmacological β-blockade can interrupt these tumor-promoting circuits in many cancer types** [5-10]**. However, clinical responses to β-adrenergic blockade are not uniform across cancers. For example, retrospective observational studies in head and neck squamous cell carcinoma have reported associations between β-blocker exposure and poorer relapse-free survival** [11]**. Given the observational nature of these data and potential confounding factors, these findings should be interpreted cautiously. Such variability may reflect differences in neural innervation patterns, tumor-specific β-adrenergic receptor expression across cancer, stromal, and immune compartments, and systemic physiological effects of adrenergic blockade that may influence tumor progression differently across clinical contexts.**

GPCR signaling can also be diversified through ligand-dependent signaling bias, whereby different ligands acting on the same receptor preferentially activate distinct downstream signaling pathways[12]. This concept is mechanistically distinct from receptor subtype specificity. For example, cholinergic signaling promotes tumorigenesis through muscarinic M3 receptor activation in gastric cancer while M1 receptor signaling suppresses pancreatic ductal adenocarcinoma growth[13, 14]. This contrast illustrates receptor subtype–specific signaling rather than biased agonism, yet it similarly demonstrates how distinct receptor signaling programs within a shared neurotransmitter system can generate opposing biological outcomes.

A comparable signaling diversity is also observed in other monoaminergic pathways. Serotonin signaling supports prostate cancer cell survival through specific receptor-mediated pathways[15], while receptor antagonism can induce apoptosis through NF-κB suppression and activation of pro-apoptotic signaling cascades[15]. **Beyond receptor diversity, serotonergic signaling outcomes can also diverge depending on ligand source and tissue compartment. Elevated systemic serotonin has been shown to suppress CD8⁺ T-cell effector function and thereby facilitate tumor progression in colorectal cancer models**[16, 17]. In contrast, within the tumor microenvironment CD8⁺ T cells can produce serotonin locally, thereby creating a localized serotonergic niche. In this context, the serotonin transporter (SERT) regulates extracellular serotonin availability through reuptake rather than synthesis, thereby modulating the magnitude and duration of serotonergic signaling. Under these conditions, SERT inhibition by selective serotonin reuptake inhibitors (SSRIs) can increase intratumoral serotonin availability and enhance CD8⁺ T-cell antitumor activity through the 5-HTR–MAPK–TCR signaling axis[18]**.** **These findings highlight that serotonergic signaling outcomes depend not only on receptor subtype but also on ligand origin, local concentration gradients, and systemic versus intratumoral signaling compartments.**

**Neuropeptide signaling further illustrates how ligand source and receptor engagement can produce bidirectional tumor effects. Met-enkephalin (Met-ENK) has been reported to exert anti-tumor activity by remodeling the tumor immune microenvironment, increasing M1 macrophages and CD8⁺ T-cell infiltration, downregulating immune checkpoint pathways, and directly suppressing tumor cell proliferation through the opioid growth factor receptor (OGFr)**[19-21]. Notably, OGFr signaling primarily mediates cytostatic effects by regulating cyclin-dependent kinase inhibitors and cell-cycle progression rather than directly inducing apoptosis. However, Met-ENK produced by tumor cells has also been reported to suppress tumor-infiltrating lymphocytes and correlate with advanced disease progression[22]. **These opposing outcomes are consistent with a model in which distinct cellular sources (exogenous or neuron-derived versus tumor-derived), local peptide concentration, and differential engagement of OGFr versus classical opioid receptors collectively determine whether Met-ENK enhances immune surveillance or promotes immune escape within the tumor microenvironment**[19-22]**.**

In addition to ligand bias, GPCR signaling can occur from spatially distinct cellular compartments, including signaling endosomes, and may interact with receptor tyrosine kinase pathways such as EGFR or integrin-associated signaling networks [10.1038/nature12000]. Together, these multilayered regulatory mechanisms—encompassing receptor subtype diversity, ligand-dependent signaling bias, ligand source and concentration, and spatial organization of signaling complexes—provide a framework for interpreting the heterogeneous biological consequences of neural signaling across tumor types and microenvironmental contexts.

References

1. Lefkowitz RJ, Shenoy SK: Transduction of receptor signals by beta-arrestins. Science. 2005; 308:5721

2. Irannejad R, Pessino V, Mika D, Huang B, Wedegaertner PB, Conti M et al: Functional selectivity of GPCR-directed drug action through location bias. Nat Chem Biol. 2017; 13:7

3. Wootten D, Christopoulos A, Marti-Solano M, Babu MM, Sexton PM: Mechanisms of signalling and biased agonism in G protein-coupled receptors. Nat Rev Mol Cell Biol. 2018; 19:10

4. Shenoy SK, Lefkowitz RJ: β-Arrestin-mediated receptor trafficking and signal transduction. Trends Pharmacol Sci. 2011; 32:9

5. Renz BW, Takahashi R, Tanaka T, Macchini M, Hayakawa Y, Dantes Z et al: β2 Adrenergic-Neurotrophin Feedforward Loop Promotes Pancreatic Cancer. Cancer Cell. 2018; 33:1

6. Zahalka AH, Arnal-Estapé A, Maryanovich M, Nakahara F, Cruz CD, Finley LWS et al: Adrenergic nerves activate an angio-metabolic switch in prostate cancer. Science. 2017; 358:6361

7. Nuevo-Tapioles C, Santacatterina F, Stamatakis K, Núñez de Arenas C, Gómez de Cedrón M, Formentini L et al: Coordinate β-adrenergic inhibition of mitochondrial activity and angiogenesis arrest tumor growth. Nat Commun. 2020; 11:1

8. Barathova M, Grossmannova K, Belvoncikova P, Kubasova V, Simko V, Skubla R et al: Impairment of Hypoxia-Induced CA IX by Beta-Blocker Propranolol-Impact on Progression and Metastatic Potential of Colorectal Cancer Cells. Int J Mol Sci. 2020; 21:22

9. Cecilio HP, Valente VB, Pereira KM, Kayahara GM, Furuse C, Biasoli É R et al: Beta-adrenergic blocker inhibits oral carcinogenesis and reduces tumor invasion. Cancer Chemother Pharmacol. 2020; 86:5

10. Chang A, Botteri E, Gillis RD, Löfling L, Le CP, Ziegler AI et al: Beta-blockade enhances anthracycline control of metastasis in triple-negative breast cancer. Sci Transl Med. 2023; 15:693

11. Chen HY, Zhao W, Na'ara S, Gleber-Netto FO, Xie T, Ali S et al: Beta-Blocker Use Is Associated With Worse Relapse-Free Survival in Patients With Head and Neck Cancer. JCO Precis Oncol. 2023; 7

12. Kenakin T: Biased Receptor Signaling in Drug Discovery. Pharmacol Rev. 2019; 71:2

13. Hayakawa Y, Sakitani K, Konishi M, Asfaha S, Niikura R, Tomita H et al: Nerve Growth Factor Promotes Gastric Tumorigenesis through Aberrant Cholinergic Signaling. Cancer Cell. 2017; 31:1

14. Renz BW, Tanaka T, Sunagawa M, Takahashi R, Jiang Z, Macchini M et al: Cholinergic Signaling via Muscarinic Receptors Directly and Indirectly Suppresses Pancreatic Tumorigenesis and Cancer Stemness. Cancer Discov. 2018; 8:11

15. Cinar I, Sirin B, Halici Z, Palabiyik-Yucelik SS, Akpinar E, Cadirci E: 5-HT7 receptors as a new target for prostate cancer physiopathology and treatment: an experimental study on PC-3 cells and FFPE tissues. Naunyn Schmiedebergs Arch Pharmacol. 2021; 394:6

16. Chan YL, Lai WC, Chen JS, Tseng JT, Chuang PC, Jou J et al: TIAM2S Mediates Serotonin Homeostasis and Provokes a Pro-Inflammatory Immune Microenvironment Permissive for Colorectal Tumorigenesis. Cancers (Basel). 2020; 12:7

17. Schneider MA, Heeb L, Beffinger MM, Pantelyushin S, Linecker M, Roth L et al: Attenuation of peripheral serotonin inhibits tumor growth and enhances immune checkpoint blockade therapy in murine tumor models. Sci Transl Med. 2021; 13:611

18. Li B, Elsten-Brown J, Li M, Zhu E, Li Z, Chen Y et al: Serotonin transporter inhibits antitumor immunity through regulating the intratumoral serotonin axis. Cell. 2025; 188:14

19. Wang X, Li S, Yan S, Shan Y, Wang X, Jingbo Z et al: Methionine enkephalin inhibits colorectal cancer by remodeling the immune status of the tumor microenvironment. Int Immunopharmacol. 2022; 111

20. Zagon IS, McLaughlin PJ: Opioid growth factor (OGF) inhibits anchorage-independent growth in human cancer cells. Int J Oncol. 2004; 24:6

21. Wang X, Jiao X, Meng Y, Chen H, Griffin N, Gao X et al: Methionine enkephalin (MENK) inhibits human gastric cancer through regulating tumor associated macrophages (TAMs) and PI3K/AKT/mTOR signaling pathway inside cancer cells. Int Immunopharmacol. 2018; 65

22. Ohmori H, Fujii K, Sasahira T, Luo Y, Isobe M, Tatsumoto N et al: Methionine-enkephalin secreted by human colorectal cancer cells suppresses T lymphocytes. Cancer Sci. 2009; 100:3
